# Human Pluripotent Stem Cell-Derived Neurons Are Functionally Mature In Vitro and Integrate into the Mouse Striatum Following Transplantation

**DOI:** 10.1007/s12035-020-01907-4

**Published:** 2020-04-30

**Authors:** Andrea Comella-Bolla, Javier G. Orlandi, Andrés Miguez, Marco Straccia, María García-Bravo, Georgina Bombau, Mireia Galofré, Phil Sanders, Jordi Carrere, José Carlos Segovia, Joan Blasi, Nicholas D. Allen, Jordi Alberch, Jordi Soriano, Josep M. Canals

**Affiliations:** 1 Laboratory of Stem Cells and Regenerative Medicine, Department of Biomedicine, Faculty of Medicine and Health Science, University of Barcelona, Barcelona, Spain; 2 Production and validation center of advanced therapies (Creatio), Faculty of Medicine and Health Science, University of Barcelona, Barcelona, Spain; 3 Institute of Neurosciences, University of Barcelona, Barcelona, Spain; 4 Networked Biomedical Research Centre for Neurodegenerative Disorders, Barcelona, Spain; 5 August Pi i Sunyer Biomedical Research Institute (IDIBAPS), Barcelona, Spain; 6 Pathophysiology of Neurodegenerative Disease. Laboratory, Department of Biomedicine, Faculty of Medicine and Health Science, University of Barcelona, Barcelona, Spain; 7 Departament de Física de la Matèria Condensada, University of Barcelona, Barcelona, Spain; 8 Department of Physics and Astronomy, University of Calgary, Calgary, Canada; 9 Differentiation and Cytometry Unit, Division of Hematopoietic Innovative Therapies, Centro de Investigaciones Energéticas Medioambientales y Tecnológicas and Centro de Investigación Biomédica en Red de Enfermedades Raras (CIEMAT/CIBERER), Madrid, Spain; 10 Advanced Therapies Unit, Instituto de Investigación Sanitaria Fundación Jiménez Díaz (IIS-FJD, UAM), 28040 Madrid, Spain; 11 Laboratory of Cellular and Molecular Neurobiology, Department Pathology and Experimental Therapeutics, Faculty of Medicine and Health Science, Biomedical Research Institute of Bellvitge (IDIBELL), University of Barcelona, Barcelona, Spain; 12 Cardiff Repair Group, School of Biosciences and medicine, Cardiff University, Cardiff, Wales UK; 13 Institute of Complex Systems (UBICS), University of Barcelona, Barcelona, Spain

**Keywords:** Telencephalon, Neuronal differentiation, Striatum, Calcium imaging, Spike-inference analysis, Transplantation

## Abstract

Human pluripotent stem cells (hPSCs) are a powerful tool for modelling human development. In recent years, hPSCs have become central in cell-based therapies for neurodegenerative diseases given their potential to replace affected neurons. However, directing hPSCs into specific neuronal types is complex and requires an accurate protocol that mimics endogenous neuronal development. Here we describe step-by-step a fast feeder-free neuronal differentiation protocol to direct hPSCs to mature forebrain neurons in 37 days in vitro (DIV). The protocol is based upon a combination of specific morphogens, trophic and growth factors, ions, neurotransmitters and extracellular matrix elements. A human-induced PSC line (Ctr-Q33) and a human embryonic stem cell line (GEN-Q18) were used to reinforce the potential of the protocol. Neuronal activity was analysed by single-cell calcium imaging. At 8 DIV, we obtained a homogeneous population of hPSC-derived neuroectodermal progenitors which self-arranged in bi-dimensional neural tube-like structures. At 16 DIV, we generated hPSC-derived neural progenitor cells (NPCs) with mostly a subpallial identity along with a subpopulation of pallial NPCs. Terminal in vitro neuronal differentiation was confirmed by the expression of microtubule associated protein 2b (Map 2b) by almost 100% of hPSC-derived neurons and the expression of specific-striatal neuronal markers including GABA, CTIP2 and DARPP-32. HPSC-derived neurons showed mature and functional phenotypes as they expressed synaptic markers, voltage-gated ion channels and neurotransmitter receptors. Neurons displayed diverse spontaneous activity patterns that were classified into three major groups, namely “high”, “intermediate” and “low” firing neurons. Finally, transplantation experiments showed that the NPCs survived and differentiated within mouse striatum for at least 3 months. NPCs integrated host environmental cues and differentiated into striatal medium-sized spiny neurons (MSNs), which successfully integrated into the endogenous circuitry without teratoma formation. Altogether, these findings demonstrate the potential of this robust human neuronal differentiation protocol, which will bring new opportunities for the study of human neurodevelopment and neurodegeneration, and will open new avenues in cell-based therapies, pharmacological studies and alternative in vitro toxicology.

**Electronic supplementary material:**

The online version of this article (10.1007/s12035-020-01907-4) contains supplementary material, which is available to authorized users.

## Introduction

Neurogenesis is a complex and highly regulated process directed by evolutionary conserved signalling molecules [1]. Despite intensive research, many aspects of neurogenesis and neurodevelopment remain to be elucidated. Furthermore, recent evidence indicates that neurodegenerative disorders such as Huntington’s disease (HD) display alterations early during neurogenesis that may contribute to neurodegeneration later in life [2–4]. Thus, methods to facilitate the understanding of neurogenesis and the contribution of neurodevelopmental alterations to neurodegenerative diseases are required.

In recent years, human embryonic stem cells (hESCs) and human-induced pluripotent stem cells (hiPSCs), collectively known as human pluripotent stem cells (hPSCs), have emerged as versatile and powerful tools for research and applied medicine given their ability to differentiate into any cell type of the human body including neurons [5]. Thus, hPSCs provide a powerful tool to gain insight into the mechanisms underlying human neurogenesis and enhance understanding of how neurodevelopment is altered in neurodegenerative diseases. Furthermore, hPSC-based therapies for the treatment of neurodegenerative diseases are possible by the differentiation of hPSCs to the affected neuronal type for subsequent transplantation into patients.

Differentiating hPSCs to a specific neuronal type is a complex endeavour that requires a robust effective in vitro differentiation protocol that mimics in vivo neurodevelopment through the addition of the necessary morphogens, growth factors, small molecules and other components at the relevant concentration and time [6–8]. In vitro neuronal differentiation can be broadly divided into three main phases, namely neural induction, neural progenitor patterning and terminal neuronal differentiation and maturation.

Neural induction of hPSCs requires the inhibition of Wnt and bone morphogenetic protein (BMP) signalling which would otherwise drive the hPSCs to a non-neural fate [9]. Indeed, it was observed that the absence of BMP signalling induces the expression of neural-specific factors [10]. Inhibition of the transforming growth factor beta (TGFβ) and canonical Wnt pathways are also required for neural induction as these pathways are implicated in maintaining pluripotency [11, 12].

During neurodevelopment, neural progenitor cells (NPCs) are patterned along the dorsoventral (DV) and anteroposterior (AP) axes of the neural tube to generate the progenitors that will produce the numerous different neuronal types of the forebrain, midbrain and hindbrain [1]. Following specification of a forebrain identity, gradients of TGFβ and BMP signalling specify a dorsal NPC identity which gives rise to cortical neurons [13]. Conversely, Sonic hedgehog (SHH) signalling induces a ventral identity which produces the ganglionic eminence NPCs that give rise to striatal neurons among others [14, 15].

Following the differentiation of the patterned NPCs to neurons, terminal neuronal differentiation and maturation are required for neuronal survival, plasticity and brain circuit integrity and function. For neurons to correctly differentiate and mature, they must progressively gain excitability, and acquire passive and active neuronal properties able to generate and transmit action potential [16]. Neuronal maturation depends on the synergistic action of developmental transcription factors (TFs), the activation or inhibition of intracellular signalling pathways, cell-to-cell interactions, and extracellular factors such as neurotransmitters (NTs), neurotrophic factors or ionic concentrations [17].

The generation of functional mature neurons differentiated from hPSCs is essential for the study of neuronal differentiation and also neuronal activity at both the individual neuron and network level. We have previously reported that the application of a cocktail of soluble molecules to hPSC-derived NPCs generates electrophysiologically active neurons that display relative negative membrane potentials and elicit spontaneous trains of action potentials [18].

A successful neuronal differentiation protocol must be robust and generate a similar outcome with different hPSC lines. Ideally, a neuronal differentiation protocol should generate functionally mature neurons in a relatively short amount of time to accelerate investigation. Furthermore, the differentiation of the cells as they progress through the protocol must be well-characterised at the gene expression level to demonstrate that the protocol mirrors the gene expression dynamics that occur in vivo. Also, the neurons that are generated should be characterised at the functional level to demonstrate that their activity is comparable with that of the equivalent in vivo neurons. Finally, in addition to in vitro analyses, from a cell therapy perspective, it must be possible to transplant the hPSC-derived NPCs into the relevant brain area where they should survive, differentiate into neurons and integrate into the host brain circuitry.

In this work, we provide a step-by-step characterisation at the gene expression and functional levels of the differentiation of hiPSC and hESC lines to functionally mature ventral forebrain neurons, including striatal medium spiny neurons, in a relatively short period of time. We observed the production of patterned NPCs after 16 days in vitro (DIV) which subsequently differentiated to spontaneously active and synaptically mature neurons in a further 21 days (37 DIV). Gene expression analysis using our high-throughput quantitative PCR (qPCR) platform to assess forebrain neuronal differentiation revealed gene expression dynamics similar to those observed during neurodevelopment [19]. Spontaneous neuronal activity was demonstrated using a single-cell calcium (Ca^2+^) imaging assay in combination with a novel semi-automatic MATLAB-based software toolbox (NETCAL) that we developed previously [20]. We also transplanted hPSC-derived NPCs into neonatal mouse striatum where they differentiated to neurons, survived for a prolonged period, projected axons to the relevant host brain target areas and established synaptic connections.

Overall, we present a fast and robust method for the differentiation of hPSC to neurons which will help advance the study of human neurodevelopment, the treatment of neuronal disorders, and will facilitate the design of high-throughput drug screening and in vitro neurodevelopmental toxicology procedures and stem cell-based therapies.

## Material and Methods

### Animals

B6CBA mice were obtained from Jackson Laboratory (Bar Harbor, ME, USA). The animals were housed with access to food and water ad libitum in a colony room kept at 19–22 °C and 40–60% humidity, under a 12:12-h light/dark cycle. Experiments were carried out according to the European regulation (2010/63/UE) for the care and use of laboratory animals.

### Human Stem Cell Lines

We compared the control (Ctr) Ctr-Q33, a hiPSC line, with 33 CAG repeats (generous gift from C. N. Svendsen, Cedar Sinai, Los Angeles, CA; USA: CS83iCTR-33nxx) and GENEA (GEN)-Q18, an ESC line, with 15/18 CAGs (GENEA019; derived from GENEA Biocells; Australia; hPSCreg Name: GENEAe020-A). All cells were regularly tested for mycoplasma contamination and karyotype normality. Human cells are growth in a 37 °C incubator with a humidified atmosphere and 5% CO2. G-band karyotypes were made in an external centre, Ambar-Anàlisis Mèdicas (Barcelona, Spain).

### GEN-Q18 hESC Expansion

GEN-Q18 hESC was cultured in feeder-free matrigel coated 6-well plates in 2.5 ml of Genea M2 Medium (MED-02–250 ml; 17G13CCMO23; Genea Biocells), a serum-free fully-defined medium containing bovine serum albumin (BSA), bFGF, Activin A and growth factors. Medium was changed every 2 days and daily in the 2 days before passaging due to the higher cell confluency.

After a minimum of 4 passages in Genea M2 medium, GEN-Q18 hESC was adapted and expanded in mTESR1 medium as we describe below.

### Human PSC Maintenance in mTeSR1 Media

All hPSCs were cultured on matrigel (354230; Corning Inc., NY, USA) feeder-free coated 6-well plates in 2 ml of serum-free defined mTeSR1 medium which contains recombinant human bFGF and recombinant human TGFβ (complete mTeSR1: mTeSR1 basal medium (05850) and mTeSR1 supplemented 5x (05852); StemCell Technologies Inc., MA, USA). Medium was daily changes (every 24 h ± 2).

### Human Pluripotent Stem Cells Passaging Protocol with ROCK Inhibitor

HPSC cultures were passaged when cell confluence reached 70–80% (twice per week). Cells were gently washed twice with 2 ml of Dulbecco’s (D)-phosphate-buffered saline (PBS) without calcium and magnesium (D-PBS^−/−^, 14,190–250; Thermo Fisher Scientific Inc., MA, USA) and incubated for 20–25 min with dispase in DMEM/F12 (07923; Stem cell Technologies, Cambridge, UK) with 10 μM of ROCK inhibitor (Y-27632; EVOTEC Ltd. Milton, UK) at 37 °C. Then, dispase was removed and cells were harvested in DMEM/F12 (Stem cell Technologies) media by pipetting up and down using a 5-ml pipette to avoid single-cell desegregation. Cells were centrifuged at 1000 rpm for 3 min and resuspended with warm and fresh Complete-mTeSR1 medium with 10 μm of ROCK inhibitor (Abcam Inc.) at the desired dilution. Then, cell suspension was seeded on tempered matrigel coated 6-well plates. Differentiated colonies were removed manually using a stereotypic microscope and a 10-ml sterile tip prior passaging.

### Human PSC Neuronal Differentiation Protocol

**Fig. 1:**
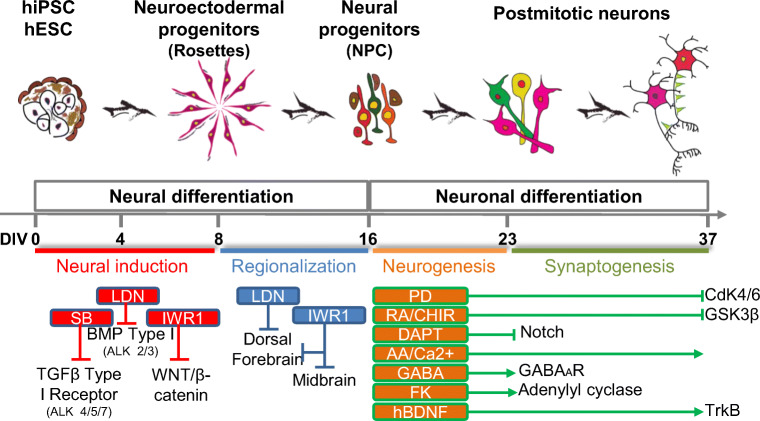
Sketch of the protocol for the in vitro differentiation of hPSCs into mature forebrain neurons in 37 DIV. The first phase consists of the neural induction of pluripotent stem cell colonies with dual-SMAD and Wnt inhibition. During the second phase, NE progenitors are regionalized to ventral telencephalic neural progenitors by inhibition of both dorsal Wnt and BMP morphogens. Finally, during the third phase, neuronal differentiation and maturation are promoted by applying a cocktail of small molecules that stimulate cell cycle exit and neuronal maturation

A schematic illustration of the differentiation protocol is shown in Fig. 1. At 60–70% of cell confluence, hPSC cultures were washed 3 times with 2 ml of D-PBS with calcium and magnesium (PBS^+/+^, 14040-091; Thermo Fisher Scientific Inc., MA, USA). The medium was then replaced by neural induction SLI medium from 0 DIV until 8 DIV (SLI culture media: Advanced DMEM/F-12(1X) (12634-010; Thermo Fisher Scientific Inc.) with 2 mM of GlutaMax™ 100x (35050-038, Thermo Fisher Scientific Inc.), 1% penicillin/streptomycin (P/S; 15140122; Thermo Fisher Scientific Inc., MA, USA); 10 μM SB 431542 (SB; 447536; EVOTEC Ltd. Milton, UK.); 1 μM LDN 193189 (396388; EVOTEC Ltd. Milton, UK); 1.5 μM IWR1 (476979; EVOTEC Ltd. Milton, UK), and 2% B27-supplemented without RA (50X) (12587-010; Thermo Fisher Scientific Inc.)).

At 4 DIV, confluent cultures were treated with 10 μM of ROCK inhibitor (Abcam Inc.) for 1 h, washed twice with 2 ml of PBS without calcium and magnesium (Thermo Fisher Scientific Inc.), incubated with accutase (StemPro A11105; Thermo Fisher Scientific Inc.) for 7 min for cell dissociation and passaged onto matrigel-coated plates, with a split ratio of 1:2. On 8 DIV, cultures were passaged 1:2 and cultured in LI medium until 15 DIV (LI culture media: Advanced DMEM/F-12(1X) (Thermo Fisher Scientific Inc.) with 2 mM of GlutaMax ™ 100x (Thermo Fisher Scientific Inc.), 1% P/S (Thermo Fisher Scientific Inc.), 200 nM LDN 193189, 1.5 μM IWR1 and 2% B27-supplemented without RA (50X) (Thermo Fisher Scientific Inc.)).

Cultures received daily medium changes of 2 ml except from DIV 5 to DIV 7 (both included), when 4 ml of fresh medium were added. At 16 DIV, NPCs were washed 3 times in PBS^−/−^ (Thermo Fisher Scientific Inc.), dissociated with accutase for 5 min and plated with a population of 8 × 10^4^ cells in 12-mm-diameter glass coverslips (treated and coated). Prior to use, coverslips were cleaned with 70% nitric acid, washed 5 times with deionized water and one time with absolute ethanol (1009832500; CAS 64-17-5; MERCK Millipore, Darmstadt, Germany) and oven sterilized at 180 °C o.n. CSs were coated first with 100 μg/ml poly-D-lysine (P0899-100 mg; Sigma-Aldrich) in borate buffer (15663; Fluka-Sigma-Aldrich Chemie GmbH; Steinheim, Germany) and with matrigel. Cells were differentiated for additional 21 DIVs in SCM1/2 media (SCM1 from 16 DIV until 23 DIV and SCM2 from 23 DIV until 37 DIV changed every 2–3 days) (SCM1 culture media (changed at 16 DIV, 18 DIV and 21 DIV): Advanced DMEM/F-12 (1X) (Thermo Fisher Scientific Inc.) with 2 mM of GlutaMax™ 100x (Thermo Fisher Scientific Inc.); 1% P/S (Thermo Fisher Scientific Inc.); 2% B27-supplemented (50X) (17504-044; Thermo Fisher Scientific Inc.); 2 μM PD 0332991 (482855; EVOTEC Ltd. Milton, UK); 10 μM DAPT (396736; EVOTEC Ltd. Milton, UK); 0.6 mM CaCl2 (to give 1.8 mM final complete medium; MERCK, CAT, Spain), 200 μM ascorbic acid (AA; A4544-100G; Sigma-Aldrich, Madrid, Spain), 10 ng/ml hBDNF (450-02; Peprotech Inc., NJ, USA); 10 μM Forskolin (FK,000087; EVOTEC Ltd.), 3 μM CHIR 99021 (CH; 401952; EVOTEC Ltd. Milton, UK) and 300 μM GABA (0.44; Tocris Cookson Inc., MO, USA)). (SCM2 culture media: (1:1) of Advanced DMEM/F-12 (1X) (Thermo Fisher Scientific Inc.) with 2 mM of GlutaMax™ 100x (Thermo Fisher Scientific Inc.): Neurobasal A (10888-022; Thermo Fisher Scientific Inc.); 1% P/S (Thermo Fisher Scientific Inc.), 2% B27-supplemented (50X) (Thermo Fisher Scientific Inc.); 2 μM PD (EVOTEC Ltd.); 3 μM CH (EVOTEC Ltd.); 0.3 mM CaCl2 (to give 1.8 mM final complete medium MERCK); 200 μM AA (Sigma) and 10 ng/ml hBDNF (Peprotech Inc.)).

### NPC Transplantation in Neonatal Mouse Striatum

To track the cells after transplants, Ctr-Q33 hiPSC line was transduced with green fluorescent protein (GFP) lentivirus under the control of the constitutive elongation factor alpha (EF1-α) promoter during WiCell maintenance. Lentivirus was incubated for 2 h at 37 °C. Cultures were grown and maintained in WiCell procedure until cell sorting by FACS (fluorescence-activated cell sorting). After three passages, transfected cells were sorted by the expression of GFP. Sorted cells were grown and maintained in WiCell procedure and adapted and expanded to mTesR1 medium after karyotyping test.

Ctr3-Q33-GFP NPCs at 16 DIV were harvested and resuspended in D-PBS^−/−^ (Thermo Fisher Scientific Inc.). P (postnatal day) 2 neonatal mice were anaesthetised by hypothermia and placed on ice until cessation of movement. Unilateral striatal injections were performed using a stereotaxic apparatus (Davis Kopf Instruments, Tujunga, CA, USA) and a 10-μl Hamilton syringe with a 33-gauge needle (Hamilton, Reno, NV), setting the following coordinates (millimetres): AP, + 2.3, L, + 1.4 from lambda and dorsoventral, − 1.8 from dura. Every animal received 15,000 cells diluted in 1 μl of D-PBS^−/−^ (Thermo Fisher Scientific Inc.). Upon completion of stereotaxic surgery, pups were warmed, monitored for 1 h to ensure recovery and then returned to the housing facility.

### Immunocytochemistry

HPSC-derived cultures were fixed for 20 min at room temperature (r.t.) with 200 μl/ 24-well of 4% paraformaldehyde solution (P/0840/53; Fisher Scientific UK Limited, Leicestershire, UK) and washed three times with 1x PBS and stored at 4 °C in 500 μl/ 24-well with 0.03% sodium-azide (71289; Fluka-Sigma-Aldrich) until use. For immunolabelling, samples were blocked and permeabilized for 45 min at r.t. with PTB solution (PBS 1x with 0.3% Triton X-100 (T8532; Sigma-Aldrich Quimica SL., Madrid, Spain), 0.03% sodium-azide, 1% BSA (A9647-1006; Sigma-Aldrich, Madrid, Spain) and 5% normal goat serum (NGS; S-1000; Vector Laboratories Ltd., UK) and/or 5% donkey serum (NDS; 017-000-121; Jackson Immuno Research Laboratories Inc.; PA, USA)), before being incubated overnight (o.n.) at 4 °C with the appropriate primary antibodies (Antibody references and dilutions are defined in Table 1). After o.n. incubation, primary antibodies were removed, and samples were washed 3 times for 10 min with PBS 1x upon the tilt table. Then, cells were incubated for 1–2 h at RT in darkness with fluorophore-conjugated secondary antibodies (References and dilutions are detailed in Table 1). After three washes of 10 min with PBS 1x, cells were counterstained with nuclear staining with DAPI 300 nM (D1306; Thermo Fisher Scientific). Coverslips were mounted in Fluoromount-G media (0100-01; Southern Biotech, AL, USA) and imaged using an Olympus IX71 inverted microscope equipped with an Orca Flash 4.0 camera (C11440-22CU, Hamamatsu Photonics France sucursal España, Barcelona, Spain) or the Leica SP5 TCS two-photon laser scanning confocal microscope (Leica Microsystems, Wetzlar, Germany).

**Table 1:** List of primary and secondary antibodies used in the work

Primary antibodies					
Target	Supplier	Reference	Host	Dilution	Used
5-HT	Sigma	S5545	R *	1/5000	ICC **
ChAT	Chemicon	AB144P	G *	1/100	ICC
CTIP2 (25B6)	Abcam	ab18465	Rat	1/300	ICC
DARPP-32 (H62)	Santa Cruz Biotechnology	Sc-11365	R	1/100	ICC
DARPP-32 (19A3)	Cell signalling	2306	R	1/ 500	IHC **
DLX_ pan	CHD1	PA5967	R	1/150	ICC
EBF_ pan	CHD1	PA590	R	1/200	ICC
GABA	Sigma	A2052	R	1/500	ICC
GFP	Abcam	Ab6556	R	1/500	TEM
Human Nuclei (HNA)	Millipore	MAB4383	M *	1/100	IHC
MAP2b	BD Transduction Laboratories	610460	M	1/500	ICC
MAP2(2a + 2b)(AP-20)	Sigma	M1406	M	1/500	IHC
Nestin	Millipore	MAB5326	M	1/200	ICC
OCT3–4 (C-10)	Santa Cruz Biotechnology	sc-5279	M	1/100	ICC
PAX6	Hybrido Bank	Pax6	M	1/200	ICC
PLZO (2A9)	Calbiochem	OP128	M	1/100	ICC
PSD-95	Abcam	Ab41455	R	1/150	ICC
SNAP25	Abcam	Ab41455	R	1/150	ICC
Synaptophysin	Synaptic Systems	101011	M	1/1000	ICC
TBR1	Abcam	Ab31940	R	1/500	ICC
TH	Abcam	AB152	R	1/300	ICC
ZO-1	Invitrogen	40-2200	R	1/100	ICC
Β-III tubulin	Sigma	T220	R	1/500	ICC
SNAP25 (SMI 81)	BioLegend	836306	M	1/1000	WB **
Syntaxin (HPC-1)	Gift of Dr. C. Barnstable		M	1/2000	WB
Synaptotagmin (41.1)	Synaptic Systems	105011	M	1/1000	WB
Actin (C4)	MP Biomedicals	0869100	M	1/20000	WB
Fluorophore-conjugated secondary antibodies					
Target	Supplier	Reference	Host	Dilution	Used
AF488 donkey α-M	Jackson Immuno Research (JIR)	715-545-150	D *	1/500	ICC
AF488 donkey α-R	JIR	711-545-152	D	1/500	ICC
Cy2 donkey α-G	JIR	705-225-003	D	1/100	ICC
Cy3 donkey α-R	JIR	711-165-152	D	1/500	ICC
Cy3 α-rat	JIR	712-165-150	Rat	1/500	ICC
Phalloidin-TRITC	Sigma	P1951	–	1/500	ICC
GFP-FITC	Abcam	Ab6662	G	1/200	IHC
HRP-conjugated secondary antibodies					
HRP α- M	Dako	P0161		1/5000	WB

*Host: *M* mouse; *R* rabbit; *G* goat; *D* donkey

**Used: *ICC* immunocytochemistry; *IHC* immunohistochemistry; *WB* western blot

### Immunohistochemistry

Animals were deeply anaesthetised with pentobarbital and intracardially perfused with PBS 1x and a 4% paraformaldehyde solution (P/0840/53; Fisher Scientific UK Limited, Leicestershire, UK) in 0.1 M sodium phosphate (CAS 7601-54-9; Sigma-Aldrich, Madrid, Spain). Brains were removed and post-fixed o.n. in the same solution, washed three times with PBS 1x, cryoprotected with 30% sucrose (CAS 57-50-1; Sigma-Aldrich, Madrid, Spain) in PBS 1x and frozen in dry-ice cooled methylbutane (CAS 78-78-4; Sigma-Aldrich, Madrid, Spain). Serial coronal sections (20 μm) of the brain were obtained using a cryostat (Microm 560 M, Thermo Fisher). Tissue was first incubated with a blocking solution containing PBS 1x, 0.3% Triton X-100 (Sigma-Aldrich Quimica SL.) and 5% normal horse serum (31874; Thermo Scientific, IL, USA) for 2 h at RT. Brain sections were then incubated o.n. at 4 °C with different primary antibodies diluted in the blocking solution (see Table 1). After three washes with PBS 1x, tissue was incubated for 1 h and a half at RT with specific fluorophore-conjugated secondary antibodies. Images were acquired with a Leica SP5 TCS two-photon laser scanning confocal microscope (Leica Microsystems).

### Immunogold Labelling and Transmission Electron Microscopy

For transmission electron microscopy (TEM) studies, samples were fixed with a solution of 2% PFA/0.5% glutaraldehyde in 0.1-M phosphate buffer, post-fixed with 1% osmium tetroxide, dehydrated and embedded in epoxy resin. Semi-thin sections (1 μm) were stained with methylene blue. Ultra-thin sections (70 nm) were immunolabelled with primary antibody, followed by incubation with a secondary antibody conjugated with 10 nm electron-dense colloidal gold (Aurion, Electron Microscopy Sciences). GFP antibody (Abcam) was used for detecting human cells. Images were acquired with a JEOL 1010 transmission electron microscope equipped with a CCD Orius camera (Gatan).

### Unbiased Cell Counts

#### Neural Progenitor Cell Counts by CellProfiler Software

HPSC-derived NPC populations at 16 DIV were quantified using an open-access CellProfiler software (BROAD institute, MA, USA). Nineteen random images, corresponding to 3% of a 24-well plate (1.92 cm^2^) culture, were taken with the epifluorescence Leica AF600 microscope (Leica Microsystems). Images were loaded in a customized pipeline with an initial nuclei detection by DAPI immunofluorescence and then the second channel, green, and the third channel, red, immunolabelled detected-nuclei counts.

#### Neuronal Cell-Type Counts by CAST Program

HPSC-derived neuronal cell types at 23 DIV and 37 DIV were manually counted using a nonbiased computer-assisted stereological toolbox (CAST) program (Olympus America Inc., NY, USA). We counted the 3% of a 12-mm-diameter coverslip (1.2 cm^2^) culture area.

#### Graft Size and Neuronal Counts of Transplanted Cells

Graft size was calculated by delineating the outer perimeter of GFP^+^ cells in eight transplanted mice. The volume occupied by the graft core was estimated through extrapolation of the area quantified in sections spaced 120 μm apart, by using an Olympus optical microscope and CAST stereology software. For determining the neuronal fate of transplanted cells, immunofluorescence images were acquired with a TCS SP5 confocal microscope (Leica) and manually counted using the ImageJ and CAST software. Immunolabelled cells in the regions of interest were quantified using high intensity projection of Z stacked images on five evenly-spaced coronal sections from six transplanted mice at each time point.

### Calcium Imaging

Sixteen DIV NPCs seeded upon 12-mm-diameter glass coverslips were differentiated into neurons and monitored for their spontaneous dynamics at 37 DIV through multi-neuron fluorescence calcium imaging. This technique enabled the simultaneous, large population tracking of neuronal firing by the binding of Ca^2+^ ions to a fluorescent indicator. Recordings were performed in artificial cerebrospinal fluid solution (aCSF) (pH 7.4) composed by 128 mM NaCl, 4 mM KCl, 1 mM CaCl_2_, 1 mM MgCl_2_, 45 mM sucrose, 10 mM glucose and 0.01 M HEPES. Fluo4-acetoxymethyl ester (AM) (494/506 nm) (F14201; Thermo Fisher Scientific Inc.) fluorescent Ca^2+^ sensitive indicator was used in all measurements. Prior to imaging, cultures were placed in a 35-mm-diameter glass bottom chamber (P35G-0-14-C, MatTek Corporation, MA, USA) that contained 2 μM of Fluo4-AM in a total volume of 2 ml of aCSF, and gently incubated on an orbital shaker in darkness for 15 min at RT. Cultures were then washed twice with fresh aCSF and stabilized for 5 min at 37 °C with 4 ml of fresh aCSF. The recording chamber was mounted on a Zeiss inverted microscope equipped with an Orca Flash 2.8 CMOS camera (Hamamatsu Photonics) and a light source for fluorescence. Image sequences of neuronal activity were acquired for 10 min at RT, with a size of 960 × 720 pixels, and a speed of 20 frames per second (FPS). Microscope settings combined a × 5 objective with a × 0.63 optical zoom, providing a spatial resolution of 4.40 μm/pixel and a field of view (FOV) of 4.2 × 3.2 mm^2^ that contained 3000–4000 cells. Representative bright field and fluorescence images for a particular experiment are provided in Fig. S9.

#### Neuronal Activity Analysis

Ca^2+^ imaging recordings were analysed with NETCAL [20], a custom-built software package run in MATLAB. For each experiment, about 1000–3000 neurons (soma regions) were automatically selected on the FOV of the culture based on the brightness of the neuronal soma. Each soma denoted a region of interest (ROI) spanning a square area 7 pixels wide (about 645 μm^2^). The fluorescence intensity of each ROI as a function of time, F*(t), was obtained by averaging the colour intensity across all pixels within the ROI at every frame. To reduce noise, fluorescence traces were smoothed out by applying a “moving average” filter 5 frames (250 ms) wide. Traces were next corrected for small drifts by subtracting a smoothed spline of the original signal. The spline was constructed by diving the original signal in blocks 25 s wide, and by connecting in a spline manner the average value within each block. The drift correction effectively removed large time-scale fluctuations while preserving neuronal firing traits and the original baseline fluorescence level (F_0_). Fluorescence signals were finally normalized as *F*(*t*) = 100(F*−*F*_0_)/*F*_0_ ≡ ∆*F*/*F*_0_ (%). Examples of characteristic fluorescence traces are provided in Fig. S9.

Neurons were separated in two groups (active or inactive) based on their fluorescence profiles by means of supervised machine learning algorithms. Briefly, several features of the fluorescence traces were first extracted by identifying their intervals of high activity. These intervals were set as the time spanned by the signal above a given threshold. Next, about 100 representative traces for each group were manually selected, and the rest were automatically assigned to the corresponding group through a boosting algorithm [21]. Finally, for each active neuron, spike trains were inferred through the “peeling algorithm” [22, 23]. For single-spike detection, we used a single-exponential model with a detection threshold of 0.8% ∆*F*/*F*_0_ and a decay time constant of tau = 1 s.

To describe the activity properties of neurons, we defined a set of nine neuronal activity features detailed in Fig. S10. These features include number of spikes (NS); firing rate (FR, spikes along time, Hz); inter-spike interval (ISI, s); standard deviation (SD) of ISI (s); number of bursts (B; defined as concatenated spikes with intervals smaller than 1 s); number of spikes within a burst; ISI inside a burst; inter-burst interval (IBI, s); and burst length (s).

#### Data Segregation by PCA and K-Means Methods—NETCAL

The nine neuronal features obtained from the activity analysis were transformed into a new basis by principal component analysis (PCA) [24]. The PCA data was then split into 8 significant groups through a k-means clustering algorithm [25]. The compound set of features of each of the groups was then further analysed.

### RNA Isolation and Retrotranscription

Total RNA was isolated along the neuronal differentiation using TRI Reagent (TR118; Molecular Research Center; Ohio, USA) following the manufacturers’ protocol. Ten microliters of total RNA at a concentration of 100–200 ng/μl (1 μg in total) for each sample were reverse-transcribed with random primers using the high-capacity RNA-to-cDNA Kit (4387406, Thermo Fisher Scientific). Ten microliters of retro-transcription cocktail (2 μl of 10× retrotranscription (RT) buffer, 2 μl of Random primers, 1 μl of dNTP mix; 1 μl MultiScribe reverse transcriptase) were added to each sample (20 μl total volume). After gentle mixing, the samples were incubated for 10 min at room temperature followed by 2 h at 37 °C, 10 min on ice and 10 min at 75 °C.

### High-Throughput Quantitative PCR-Openarray and Data Analysis

Open array analysis was performed as described elsewhere [19]. Complementary cDNAs from 0 DIV to 37 DIV hPSC-samples were loaded onto the custom openarrays and run as recommended by the manufacturer on the QuantStudio 12 K Flex Real-Time PCR system (Thermo Fisher Scientific) by Servei Veterinari de Genètica Molecular (Faculty of Veterinary, Universitat Autònoma de Barcelona, Cerdanyola del Vallès, Spain). We ran two openarrays, the so-called developmental openarray with 112 TaqMan probes (Thermo Fisher Scientific), and a second openarray called Ephys with 168 TaqMan probes [19]. Both openarrays specifically detected all isoforms of each gene that were selected from the literature, and included 6 housekeeping genes for the developmental (18S, B2M, HPRT1, HSP90AB1, RPL13A, UBC) and 10 housekeeping genes for the Ephys (18S, B2M, HPRT1, HSP90AB1, RPL13A, UBC, PAPOLA, ACTB, EIF2B1, TBP) that were used as reference genes. For data analysis, Ctr-Q33 and GEN-Q18 samples were ran and analysed separately. For each openarray, we took one random sample from 37 DIV as reference sample. Relative gene expression was calculated using the Expression Suite Software 1.03 (Life Technology, Barcelona, Spain). Relative quantity (RQ) minimum and maximum values (error bars) were calculated with a confidential level of 95%, using Benjamini-Hochberg false discovery rate to adjust *P* values. Maximum allowed threshold cycle Ct included in calculations is 30 and a quantitation cycle Cq confidence > 0.8. Error bars are presented in all graphs as standard error of the mean (SEM). Gene expression profile data are represented in graphics as RQ to 37 DIV.

### Protein Isolation and Western Blot

Total protein extract was isolated from human tissue using TRI Reagent (T9424, Sigma-Aldrich, Madrid, Spain) according to the manufacturer’s protocol. Total protein extracts were denatured using 1% SDS at 100 °C for 5 min. Sixteen microgram of each denatured sample was subjected to 12% SDS-PAGE and transferred to a nitrocellulose membrane (IPVH00010, Millipore, Barcelona, Spain) for 60 min at 1 mA/cm^2^. Membranes were incubated o.n. with primary antibodies (Ref. and dilution defined in Table 1) in immunoblot buffer (TBS-T-5%M; Tris-buffered saline (TBS) containing 0.05% Tween-20 and 5% no-fat dry milk) at + 4 °C in agitation and 1 h at RT upon a tilt table with HRP secondary antibodies in TBS-T-5%M. For load control, anti-alpha actin (1:20,000) was incubated for 20 min at RT in TBS-T-5%. Membrane development was performed in TBS-T-5% BSA. Membranes were developed using the Luminata Classico or Forte Western HRP Substrate (WBLUC0100 and WBLUF0100, respectively, MERCK Millipore, Darmstadt, Germany) in Fuji Medical X-Ray Films (Super RX-N; Fujifilm Co., Tokyo, Japan).

### Statistical Analysis

For comparisons between 16 DIV Ctr-Q33 and GEN-Q18 NPCs, two-way ANOVA followed by Tukey’s multiple comparison test was applied and values of *P* < 0.05 were considered statistically significant, *P < 0.05, **P < 0.005, ***P < 0.0005. Neuronal progenitor cells were represented as relative to total labelled nuclei or relative to PAX6 NPCs in double labelling. Error bars were presented in all graphs as standard error of the mean (SEM).

For comparisons between Ctr-Q33 and GEN-Q18 neuronal cell types at 23 DIV and 37 DIV, two-way ANOVA followed by Tukey’s multiple comparison test was applied, *P < 0.05, **P < 0.005, ***P < 0.0005. Neuronal cell types were represented as relative to total labelled nuclei by DAPI immunofluorescence or relative to Map 2b. Error bars were presented in all graphs as SEM.

For gene expression comparisons along the differentiation from 0 DIV to 37 DIV, one-way ANOVA followed by Tukey’s multiple comparison test was applied. Relative to 0 DIV, *P < 0.05, **P < 0.01, ***P < 0.001; to DIV 8 + *P* < 0.05, ++ *P* < 0.01, +++ *P* < 0.001; 12 DIV # *P* < 0.05, ## *P* < 0.01, ### *P* < 0.001; to DIV 16 $$ *P* < 0.01, $$$ *P* < 0.001; to 23 DIV & *P* < 0.05, && *P* < 0.01 and &&& *P* < 0.001. Error bars were presented in all graphs as SEM. Gene expression profile data were represented in graphics as RQ to 37 DIV.

For firing and bursting feature comparisons between groups, G1 to G8 at 37 DIV, one-way ANOVA followed by Tukey’s multiple comparison test was applied. *P* < 0.05 *, *P* ≤ 0.01 ** and *P* ≤ 0.001 ***. *P* > 0.05. Data is summarized in Fig. S12 and grey boxes).

Colour matrices represented all possible comparisons on the diagonal (blue box; 0 DIV to 37 DIV) on which the statistical test was carried out for all the multiple comparisons. Non-expressing genes were indicated with grey boxes. Colour boxes for expressing genes with *P* < 0.05. Upregulated (red) and downregulated (green) genes were represented with increasing three colour-scale intensities for *P* ≤ 0.5, *P* ≤ 0.005 and *P* ≤ 0.0005, respectively.

Protein quantification of Ctr-Q33 and GEN-Q18 at DIV 0, DIV 23 and DIV 37 was calculated by relative quantity to alpha-actin band. One-way ANOVA followed by Tukey’s multiple comparison test was applied and values of *P* < 0.05 were considered statistically significant. Relative to 0 DIV, *P < 0.05, **P < 0.005, ***P < 0.0005; to DIV 23 # *P* < 0.05, ## + *P* < 0.01, ### *P* < 0.001. Protein levels were represented in graphics as relative protein to alpha-actin and error bars as SEM.

### Quality System

All procedures were conducted under UNE-EN-ISO9001:2015 and comply with the Guidance Document on Good In Vitro Method Practices (GIVIMP; OECD) recommendations [26].

## Results

Two sources of hPSC cell lines, an induced Ctr-Q33 and an embryonic GEN-Q18 hPSCs, successfully differentiated into forebrain mature neurons in 37 DIV. The in vitro neuronal differentiation comprised 3 successive stages illustrated in Fig. 1. First, the protocol consisted in the neural induction and expansion of progenitor cells during the first 8 DIV. Second, neuroectodermal (NE) progenitors were regionalized into anterior forebrain or telencephalic NPCs at 16 DIV. And, third, the protocol promoted hPSC-derived NPCs cell cycle exit together with terminal neurogenesis and synaptogenesis until 37 DIV.

All gene and protein nomenclatures are detailed in Table S1.

### The Synergetic Dual-SMAD and WNT Inhibition Forces hPSCs to Exit from Pluripotency

At 0 DIV, both hPSC cell lines are arranged in monolayer colonies throughout the culture. HPSC colonies were entirely composed by pluripotent cells positive for OCT-3/4 TF (Fig. S1a and b and panels a’ and b’). HPSCs began to differentiate by using dual-SMAD inhibition along with Wnt/β-catenin inhibitor (SLI media; neural induction Fig. 1). By means of quantitative gene expression analysis, we observed that both hPSC cell lines downregulated the expression of well-known pluripotent TFs POU5F1 (OCT4), NANOG and KRT18 (Fig. S1 panels c and d).

### HPSC-Derived Neuroepithelial Progenitors Homogeneously Neuralize in Bi-dimensional Neural Tube-like Structures After 8 DIV

**Fig. 2:**
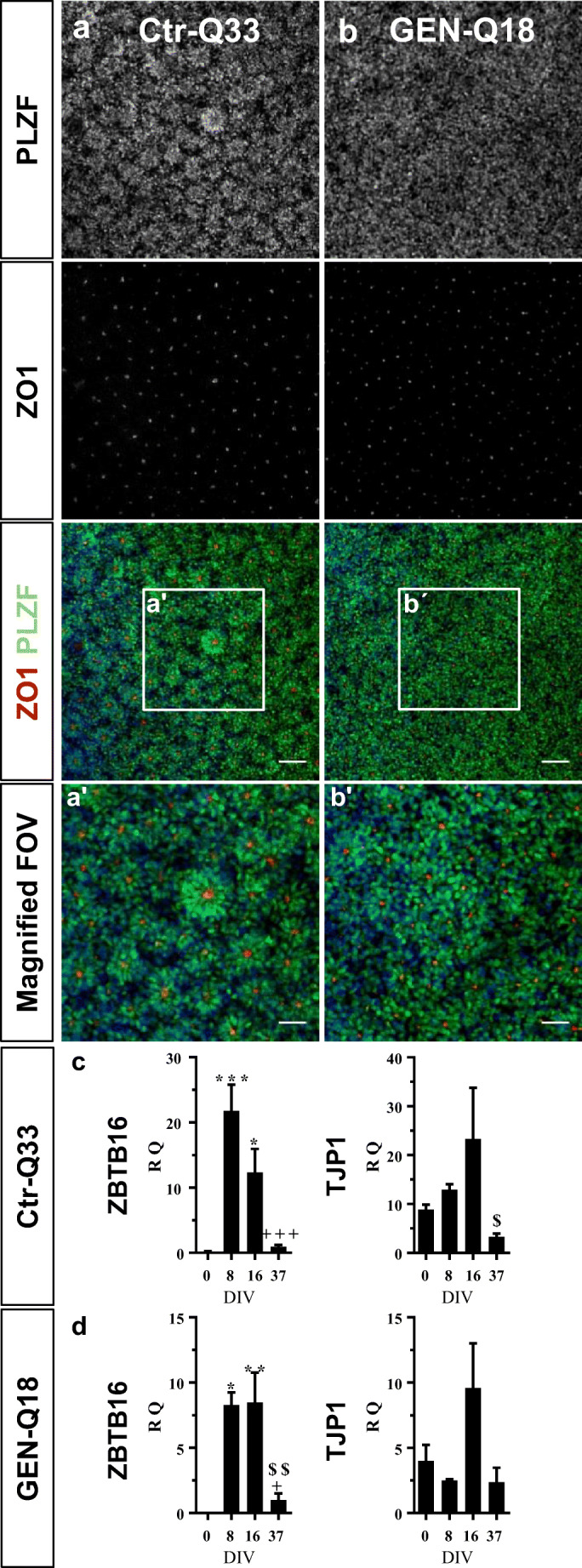
Immunostaining of neural tube-like markers, PLZF (green) and ZO1 (red) in **a** Ctr-Q33 and **b** GEN-Q18 hPSC-derived NE progenitors at DIV 8. The squared boxes (a‘and b‘) show a magnified field of view (FOV) from **a** and **b**. Scale bar 50 μm. Quantitative gene expression of ZBTB16/PLZF and TJP1/ZO1 along **c** Ctr-Q33 and **d** GEN-Q18 neuronal differentiation. (Mean ± SEM; one-way ANOVA followed by Tukey’s multiple comparison test, relative to 0 DIV *P < 0.05, **P < 0.01, ***P < 0.001; to 8 + DIV *P* < 0.05, ++ *P* < 0.01, +++ *P* < 0.001; to 16 DIV $$ *P* < 0.01, $$$ *P* < 0.001)

For neural fate specification and neurulation of hPSC, fourth additional days of BMP, TGFβ and Wnt/β-catenin inhibitors were applied to hPSC-derived cultures from 4 DIV to 8 DIV (Fig. 1; neural induction). HPSCs successfully acquired a neuroepithelial (NE) progenitor identity at 8 DIV. These NE progenitors are self-arranged in bi-dimensional neural tube-like or rosette structures (Fig. 2). Moreover, hPSC-derived NE progenitors expressed neurulation proteins ZO1, tight junction protein 1 (TJP1) and PLZF (promyelocytic leukaemia zinc finger) at protein and mRNA levels. Representative images of Ctr-Q33 and GEN-Q18 hPSC-derived NE cultures are shown in Fig. 2a and Fig. 1b, respectively. As expected, PLZF is expressed within the nucleus of NE progenitors (Fig. 2a and b), and ZO1 is expressed apically at the luminal zone of the rosettes (Fig. 2a and b). The acquisition of neural fate by hPSC-derived NE progenitors was also corroborated by gene expression profile (Fig. 2c and d). The Zing finger and BTB domain containing 16 (ZBTB16/ PLZF) gene were induced at 8 DIV by Ctr-Q33 and GEN-Q18 NE progenitors. In contrast, TJP1/ ZO1 was found throughout the neuronal differentiation but its expression was downregulated at late differentiation stages.

**Fig. 3:**
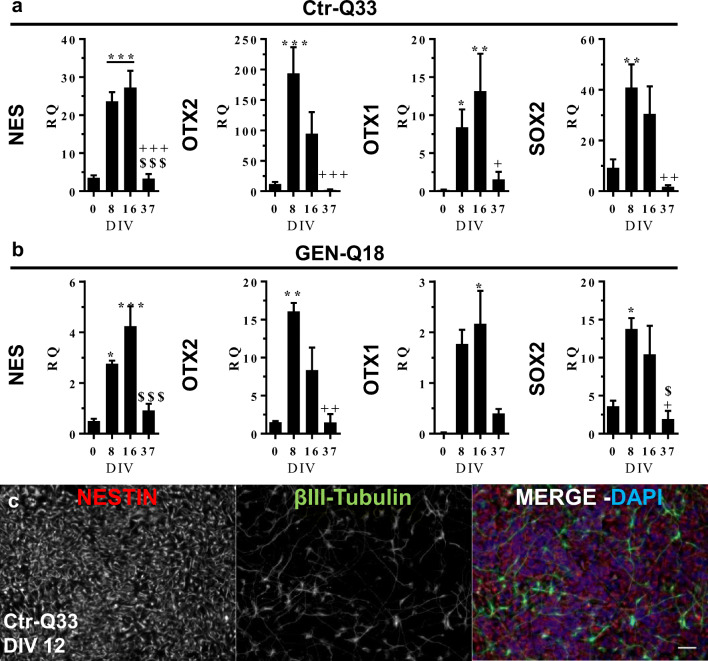
Quantitative gene expression of neural NES, OTX-2/1 and SOX2 genes by **a** Ctr-Q33 and **b** GEN-Q18 hPSC-derived cultures along the neuronal differentiation. (Mean ± SEM; one-way ANOVA followed by Tukey’s multiple comparison test, relative to 0 DIV *P < 0.05, **P < 0.01, ***P < 0.001; to 8 DIV + *P* < 0.05, ++ *P* < 0.01, +++ *P* < 0.001; to 16 DIV $$ *P* < 0.01, $$$ *P* < 0.001). **c** Immunofluorescence for the early NPC marker nestin (red) and the neuronal progenitor marker β-III tubulin (green) in Ctr-Q33 hiPSC-derived NPC cultures at DIV 12. Scale bar 50 μm

Additionally, Ctr-Q33 and GEN-Q18 hPSC-derived NE cultures induced the expression of early NPC-related genes (Fig. 3a and b, respectively). HPSC-derived NE progenitors upregulated the cytoskeleton early neural gene nestin (NES) from 8 DIV until 16 DIV. OTX-1, -2 and SOX2 genes were upregulated from 8 DIV and became downregulated at late differentiation stages (37 DIV). Neural identity of Ctr-Q33 and GEN-Q18 hPSC-derived cultures was corroborated by immunolabelling for NES and β-III tubulin. At 12 DIV, Ctr-Q33 cultures were mainly composed by nestin-positive NPC along with few progenitors positive for the neuronal marker β-III tubulin (Fig. 3c).

### Regionalization of hPSC-Derived NPCs Gives Rise to Mainly Subpallial Telencephalic Progenitor at 16 DIV

We next assessed by quantitative gene expression analysis the regionalization of hPSC-derived NPCs. Figure S2 shows the consolidation of the anterior commitment of Ctr-Q33 and GEN-Q18 hPSC-derived NPCs (S2a and b, respectively). Both cell lines induced the expression of the forebrain FOXG1, DACH, SIX3 and GLI3genes at 16 DIV. We neither detected expression of the midbrain PAX2, hindbrain HOXB 4/9 nor the spinal cord FOXA2A region-specific genes along the neuronal differentiation (data not shown). From 8 DIV to 16 DIV, Ctr-Q33 and GEN-Q18 cultures progressed towards the neuronal differentiation by expressing the neuronal genes DCX, TUBB3 and NCAM1 (doublecortin, β-III tubulin and neural cell adhesion molecule respectively; Fig. S3).

**Fig. 4:**
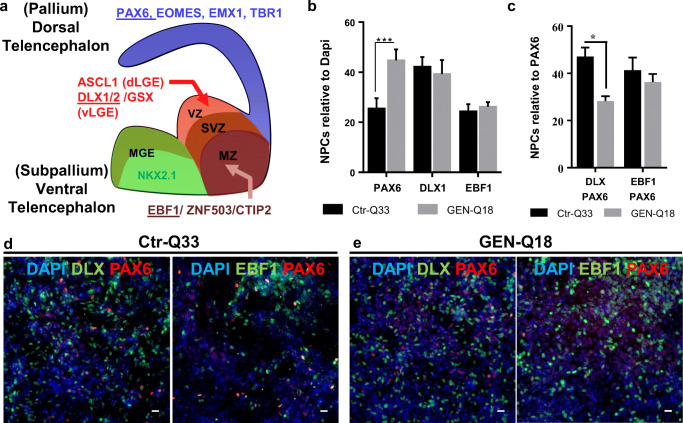
Ctr-Q33 and GEN-Q18 hPSC-derived NPC’s populations at 16 DIV. **a** Schematic representation of pallial and subpallial telencephalic region-specific TFs. **b** Proportion of PAX6+, DLX_pan+ and EBF1+ NPCs relative to total cells in Ctr3-Q33 (black) and GEN-Q18 (grey)-derived NPC cultures. **c** Proportion of ventral DLX_pan+ and EBF1+ NPCs double labelled with dorsal PAX6 progenitors. (Mean ± SEM; two-way ANOVA followed by Tukey’s multiple comparison test, *P < 0.05, **P < 0.005, ***P < 0.0005). Representative images of double-labelled PAX6 (red) with DLX_pan or EBF1 (green) in **d** Q33n1 and **e** GEN-Q18 hPSC-derived cultures at 16 DIV. Scale bar 100 μm

In order to distinguish among the different NPC subtypes, we quantified the proportions of dorsal PAX6^+^ NPCs and ventral DLX_pan^+^ and early B cell factor 1 (EBF1)^+^ NPCs present at 16 DIV (Fig. 4a). Ctr-Q33 and GEN-Q18 cultures contained similar proportions of ventral DLX^+^ and EBF1^+^ NPCs (Fig. 4b). For DLX_pan NPCs, Ctr-Q33-derived cultures contained 42 ± 2% and GEN-Q18-derived cultures 39 ± 3%. For EBF1, the proportions were slightly lower compared with DLX_pan in both cell lines, 24 ± 1% and 26 ± 1% for Ctr-Q33 and GEN-Q18, respectively. For dorsal progenitors, GEN-Q18 cultures contained more PAX6^+^ NPCs compared with Ctr-Q33 cultures at 16 DIV with 45 ± 2% and 26 ± 2%, respectively (Fig. 4b). However, since PAX6 besides specify dorsal NPCs is also expressed by NE progenitors [27], we discriminated between PAX6^+^ early NE progenitors and pallial-specific PAX6^+^ NPCs. To do so, we performed double labelling of dorsal and ventral markers (Fig. 4d and e). From the entire PAX6 population, most PAX6^+^ NPCs co-labelled with ventral markers. In Ctr-Q33-derived NPC cultures, 47% ± 3 PAX6 progenitors co-labelled with DLX_pan and 41 ± 5% with EBF1 (Fig. 4c). In GEN-Q18-derived NPC cultures, 28 ± 2% of PAX6 progenitors co-labelled with DLX_pan and 36 ± 1.3% with EBF1 (Fig. 4c). Representative images from the double-labelled dorsal and ventral NPCs in Ctr-Q33 and GEN-Q18 at DIV 16 are illustrated in Fig. 4d and e, respectively.

**Fig. 5:**
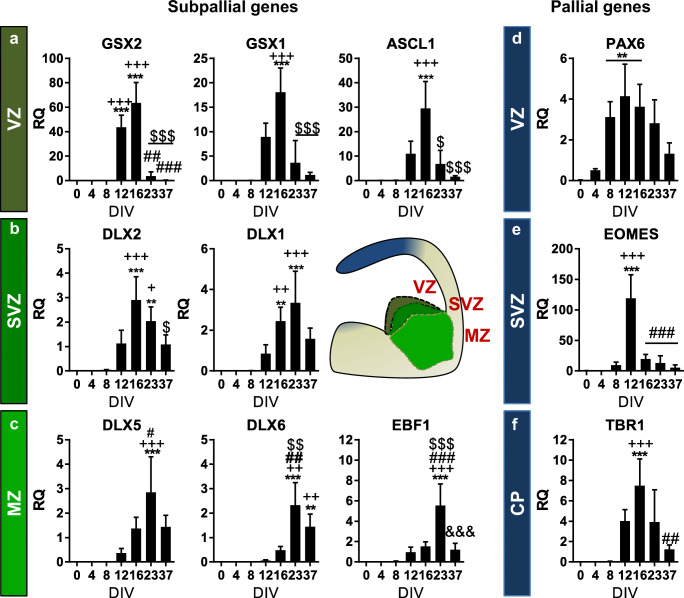
Quantitative gene expression of **a**–**c** telencephalic subpallial and **d**–**f** pallial gene expression throughout Ctr-Q33 neuronal differentiation. (Mean ± SEM; one-way ANOVA followed by Tukey’s multiple comparison test, relative to 0 DIV *P < 0.05, **P < 0.01, ***P < 0.001; to 8 DIV + *P* < 0.05, ++ *P* < 0.01, +++ *P* < 0.001; to 12 DIV # *P* < 0.05, ## *P* < 0.01, ### *P* < 0.001; to 16 DIV $ *P* < 0.05, $$ *P* < 0.01, $$$ *P* < 0.001; to 23 DIV & *P* < 0.05, && *P* < 0.01, &&& *P* < 0.001). *VZ* ventricular zone; *SVZ* subventricular zone; *MZ* mantel zone and *CP* cortical plate

Furthermore, we analysed the most relevant TFs involved in telencephalic specification. The differentiation of hPSC resembled endogenous striatal neural differentiation. From 12 DIV to 16 DIV, cells upregulated the expression of LGE VZ-related GSX2 and GSX1 genes which were afterwards downregulated from 23 DIV onwards (Fig. 5a). The LGE VZ-related ASCL1 gene displayed a single-induction peak at 16 DIV and thereafter, it became downregulated from 23 DIV onwards (Fig. 5a and S4a, for Ctr-Q33 and GEN-Q18, respectively). Interestingly, differentiated hPSC displayed a gradual expression of DLX genes from 16 DIV onwards (Fig. 5b). HPSC-derived NPCs induced the expression of the SVZ-related DLX2 and DLX1 at 16 DIV (Fig. 5b and S4b) followed by the induction of the striatal MZ-related DLX5 and DLX6 genes along with EBF1 from 23 DIV (Fig. 5c and S4c). As expected, the expression of PAX6 was found early from 8 DIV, to specify neural fate, to 16 DIV (Fig. 5d and S4d). Thereafter, the cortical SVZ-related EOMES displayed a single-induction peak at 12 DIV and became downregulated afterwards until the end of the differentiation. Finally, hPSC-derived cultures upregulated the cortical plate (CP)-related TBR1 gene at 16 DIV (Fig. 5f and S4f).

### HPSC-Derived Neurons Reached Synaptic Maturity at 23 DIV

**Fig. 6:**
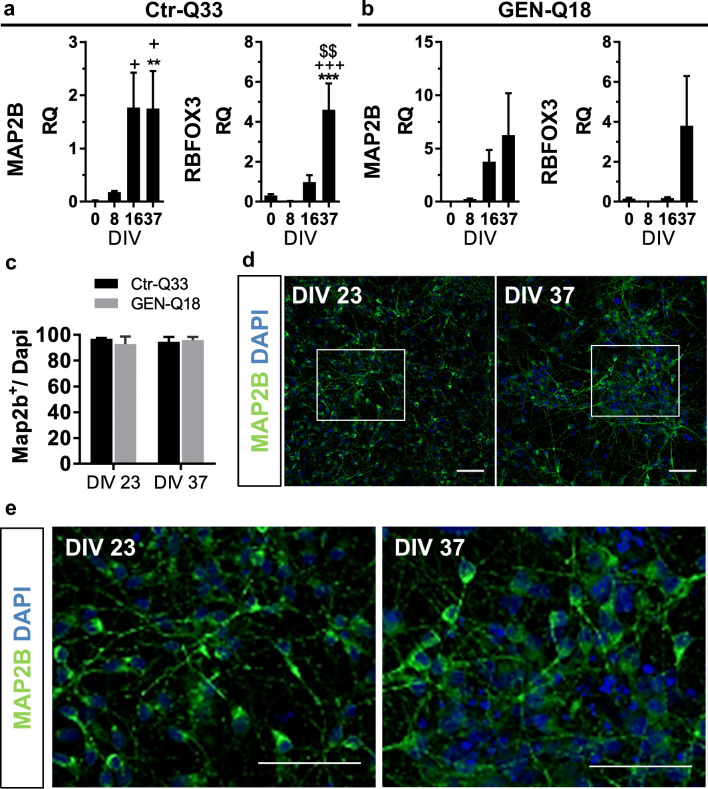
Differentiation of Ctr-Q33 and GEN-Q18 hPSC generates MAP2b neurons from 23 DIV onwards. Gene expression profile of MAP2 and RBFOX3 along **a** Ctr-Q33 and **b** GEN-Q18 neuronal differentiation (Mean ± SEM; one-way ANOVA followed by Tukey’s multiple comparison test, relative to 0 DIV *P < 0.05, **P < 0.01, ***P < 0.001; to 8 DIV + *P* < 0.05, ++ *P* < 0.01, +++ *P* < 0.001; to 16 DIV $ *P* < 0.05, $$ *P* < 0.01, $$$ *P* < 0.001). **c** Proportion of MAP2b + neurons by Ctr-Q33 (black) and GEN-Q18 (grey) at 23 and 37 DIV (Mean ± SEM; two-way ANOVA followed by Tukey’s multiple comparison test; *P* < 0.05). **d** Image of MAP2b expression by Ctr-Q33 hiPSC-derived neurons at DIV 23 and 37. **e** High-magnification images from insets in **d**. Scale bar 10 μm

HPSC-derived NPCs differentiated and generated postmitotic neuronal cultures (Fig. 6). Quantitative gene expression of mature neuronal genes MAP2 and RBFOX3 (NeuN) were induced by both hPSC cell lines starting at 16 DIV (Fig. 6a and b). Ctr-Q33 and GEN-Q18 cell lines gave rise to almost 100% of Map2b neurons from 23 DIV onwards, without differences between cell lines at 23 DIV and 37 DIV (for Ctr-Q33, 97% and 95%, and for GEN-Q18, 99% and 98% at 23 DIV and 37 DIV, respectively; Fig. 6c). Consistently, we observed that hPSC-derived neurons displayed neurites outgrowth and abundant arborisations as differentiation progressed (Fig. 6d and e).

**Fig. 7:**
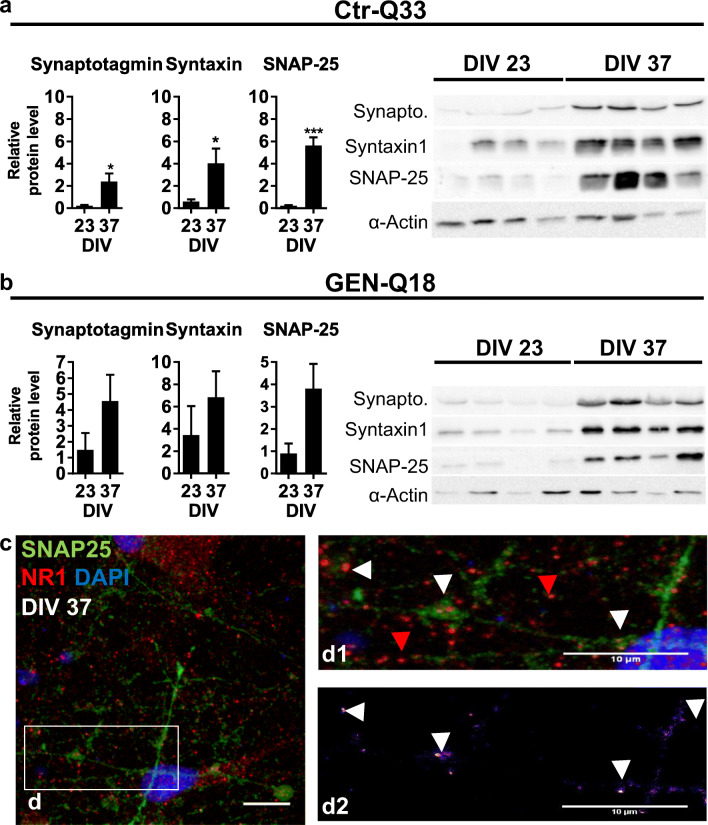
Expression of SNARE proteins by **a** CTR-Q33 and **b** GEN-Q18 from 23 DIV onwards. Relative quantity to alpha-actin. (Unpaired *t* test with Welch’s correction, relative to 23 DIV *P < 0.05, **P < 0.005 and ***P < 0.001; Mean ± SEM). **c** Staining of SNAP-25 (red) and NR1 (green) in Ctr-Q33 hiPSC-derived neurons at DIV 37 with the colocalization punctate in the magnified FOV b’1 and 2. White arrowhead indicates NR1-SNAP-25 colocalization punctate; red arrowhead single indicates SNAP-25 punctate. Scale bar 10 μm

**Fig. 8:**
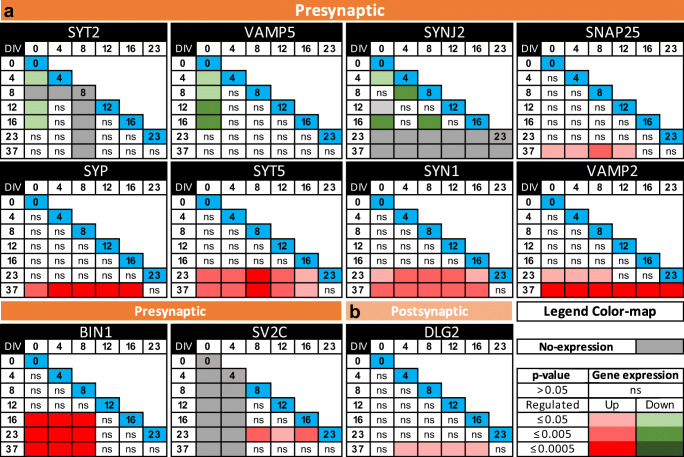
Quantitative gene expression of presynaptic and postsynaptic genes along Ctr-Q33 hiPSC neuronal differentiation. Matrices (DIV vs. DIV) for **a** presynaptic and **b** postsynaptic genes along Ctr-Q33 hiPSC neuronal differentiation. One-way ANOVA followed by Tukey’s multiple comparison test was performed between DIVs (0 to 37). Coloured matrices represent all possible comparisons on the diagonal (blue box; DIV on which the statistical test is done for all the multiple comparisons); non-expressing genes (grey boxes). Colour boxes for expressing genes with *P* < 0.05. Upregulated (red) and downregulated (green) genes are represented with increasing three colour-scale intensities for *P* ≤ 0.5, *P* ≤ 0.005 and *P* ≤ 0.0005 respectively

To confirm whether hPSC-derived neurons achieved synaptic maturation after neuronal differentiation, we analysed the expression of synaptic-related markers at protein (Fig. 7) and their gene expression levels (Fig. 8). HPSC-derived neurons expressed the soluble NSF attachment protein (SNA) REceptor (SNARE) proteins synaptotagmin, syntaxin-1 and synaptosomal-associated protein 25 (SNAP-25) at 23 DIV and 37 DIV. All three SNAREs were largely expressed at 37 DIV compared with 23 DIV (Fig. 7a and b). SNAP25 was also detected by immunofluorescence along with the postsynaptic NMDAR subunit 1 (NR1) at 37 DIV (Fig. 7c). Ctr-Q33-derived neurons displayed abundant punctate SNAP-25 staining throughout the cytosol and projections of which some SNAP-25 punctuates co-localized with NR1, as shown in Fig. 7d.

We next performed a wide screening of a set of presynaptic and postsynaptic genes along the Ctr-Q33 hiPSC neuronal differentiation (Fig. 8). Ctr-Q33-derived neurons upregulated the expression of presynaptic endocytosis-related protein BIN1 from DIV 16. Synaptotagmin V (SYT5), synapsin I (SYN1), vesicle-associated membrane protein 2 (VAMP2) and synaptic vesicle glycoprotein 2C (SV2C) genes were found induced from 23 DIV onwards (Fig. 8a). At 37 DIV, Ctr-Q33 neurons induced the gene expression of SNAP-25 and synaptophysin (SYP). Conversely, the neuronal differentiation resulted in the downregulation of SYT2, VAMP5 and SYNJ2 (Fig. 8a). Regarding postsynaptic genes, Ctr-Q33 neurons showed a significant upregulation DLG2 (PSD-93; Discs large homologues 2/postsynaptic density protein 93) at 37 DIV (Fig. 8b). Other postsynaptic proteins, such as DLG1/SAP97, DLG3/SAP102 and DLG4//PSD95, were expressed along Ctr-Q33 hiPSC differentiation but they did not show significant differences between DIVs (Fig. S17a).

### Neuronal Cultures Comprise GABA, TH and TBR1-Expressing Neurons from 23 DIV

We performed a detailed characterization of the main forebrain neuronal cell types, including GABAergic, glutamatergic, dopaminergic (DAergic) -tyrosine hydroxylase (TH)^+^ neurons and MSN-like neurons, in Ctr-Q33 and GEN-Q18 hPSC-derived neuronal cultures.

**Fig. 9:**
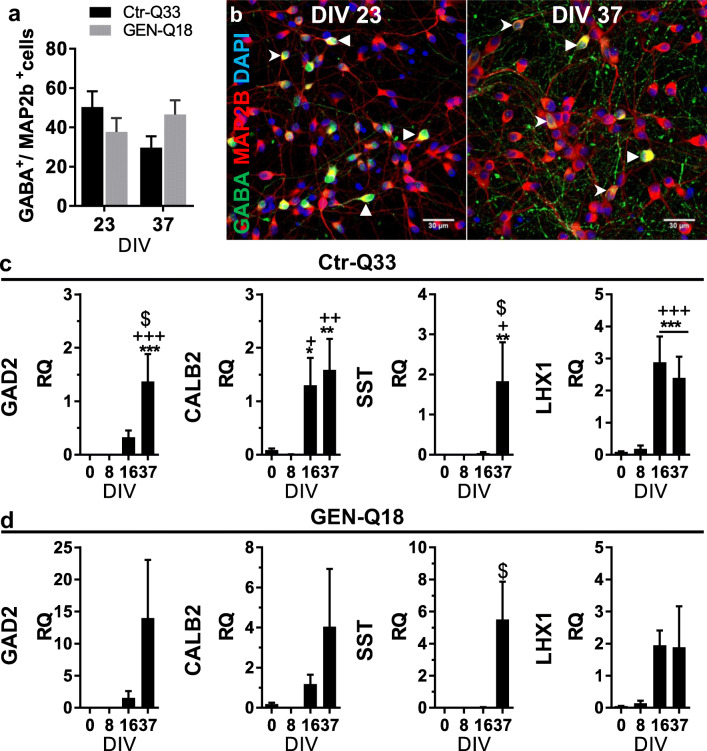
Ctr-Q33 and GEN-Q18 hPSC-derived GABA+ neurons. Proportion of GABA+ cells relative to MAP2b + in Ctr-Q33 (black) and GEN-Q18 (grey) at 23 and 37 DIV (Mean ± SEM; two-way ANOVA followed by Tukey’s multiple comparison test, ns *P* > 0.05). **b** Representative GABA (green) staining along with the neuronal MAP2b (red) and DAPI (blue) at both DIVs by Ctr-Q33 hiPSC-derived neuronal cultures. Triangular arrowheads point intense GABA+ neurons and spiky arrowheads highlight low GABA+ neurons. Scale bar 30 μm. Quantitative GABAergic-related gene expression profile along **c** Ctr-Q33 and **d** GEN-Q18 neuronal differentiation. (Mean ± SEM; one-way ANOVA followed by Tukey’s multiple comparison test, relative to 0 DIV *P < 0.05, **P < 0.01, ***P < 0.001; to 8 DIV + *P* < 0.05, ++ *P* < 0.01, +++ *P* < 0.001; to 16 DIV $ *P* < 0.05, $$ *P* < 0.01, $$$ *P* < 0.001)

Ctr-Q33 and GEN-Q18-derived neuronal cultures contained a high amount of GABA^+^/Map2b^+^ neurons at 23 DIV and 37 DIV (Fig. 9; Tables 2 and 3). Ctr-Q33-derived neuronal cultures showed a tendency to decrease the number of GABA^+^ population from 50 ± 8% at 23 DIV to 30 ± 6% at 37 DIV, whereas GEN-Q18-derived neuronal cultures showed a slight increase from 38 ± 7% at 23 DIV to 47 ± 7% at 37 DIV (Fig. 9a). Representative immunofluorescence images for these time points are shown in Fig. 9b for Ctr-Q33 and Fig. S5 for GEN-Q18, highlighting the increase of GABA^+^ projections, both in number and length, from 23 DIV to 37 DIV. GABAergic neuronal differentiation was also corroborated by quantitative analysis of the expression of a set of GABAergic-related genes (Fig. 9c for Ctr-Q33 and Fig. 9d for GEN-Q18). The cultures had already upregulated the expression of GAD2, CALB2 (calretinin) and SST (somatostatin) and the interneuron-specific LHX1 (LIM homeobox 1) gene at 16 DIV.

**Table 2:** Neuronal subtype cell counts in Ctr-Q33 hiPSC-derived neuronal cultures at 23 and 37 DIV

Ctr Q33	DARPP-32		DARPP-32/Map2b		Ctip2 (25B6)		DARPP-32/Ctip2		Map2b		GABA/Map2b		TH/Map2b		TBR1/Map2b	
DIV	23	37	23	37	23	37	23	37	23	37	23	37	23	37	23	37
Diff	4	4	4	4	4	4	4	4	4	4	4	4	4	4	4	4
CSs	2	2	2	2	2	2	2	2	2	2	2	2	2	2	2	2
Mean	0.3	6.3	0.2	6.0	61.8	54.8	0.1	5.9	97.0	94.5	50.4	29.8	19.3	35.6	3.9	7.1
SD	0.1	1.7	0.1	0.6	24.1	19.1	0.1	1.8	1.4	7.8	15.9	11.5	3.3	10.7	0.6	0.8
SEM	0.1	0.8	0.0	0.3	12.0	9.5	0.1	0.9	0.7	3.9	8.0	5.7	1.6	5.4	0.3	0.4
*P* value	0.0062*		0.0034**		0.75		0.0072*		0.0072*		0.0072*		0.0072*		0.0072*

**Table 3:** Neuronal subtype cell counts in GEN-Q18 hESC-derived neuronal cultures at 23 and 37 DIV

GEN Q18	DARPP-32		DARPP-32/Map2b		Ctip2 (25B6)		DARPP-32/Ctip2		Map2b		GABA/Map2b		TH/Map2b		TBR1/Map2b	
DIV	23	37	23	37	23	37	23	37	23	37	23	37	23	37	23	37
Diff	3	3	3	3	3	3	3	3	3	3	3	3	3	3	3	3
CSs	2	2	2	2	2	2	2	2	2	2	2	2	2	2	2	2
Mean	0.4	4.2	0.1	4.0	55.0	42.7	0.4	4.0	98.7	98.3	37.7	46.6	49.7	51.7	7.4	4.2
SD	0.8	2.6	0.2	2.4	24.0	6.0	0.8	2.4	0.2	0.7	12.1	12.4	2.0	7.6	5.9	1.8
SEM	0.4	1.5	0.1	1.4	13.9	3.5	0.4	1.4	0.2	0.5	7.0	7.2	1.2	4.4	3.4	1.0
*P* value	0.1169		0.1233		0.5298		0.1156		0.4713		0.1356		0.7281		0.3127	

**Fig. 10:**
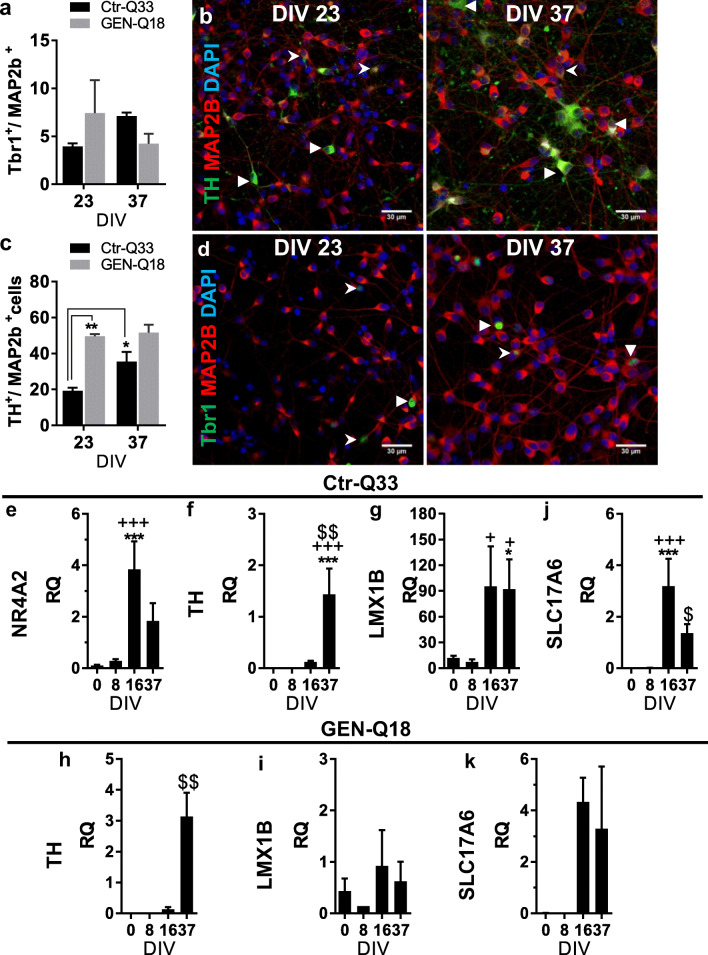
Ctr-Q33 and GEN-Q18 hPSC-derived TH+ and Tbr1+ neurons. Proportion of TH+ cells relative to MAP2b + in Ctr-Q33 (black) and GEN-Q18 (grey) at 23 and 37 DIV and **b** representative TH (green) staining along with MAP2b (red) and DAPI (blue) at both DIVs by Ctr-Q33 hiPSC-derived neuronal cultures. Triangular arrowheads point intense TH+ neurons and spiky arrowheads highlight low TH+ neurons. Scale bar 30 μm. **c** Proportion of Tbr1+ cells relative to MAP2b + in Ctr-Q33 (black) and GEN-Q18 (grey) at 23 and 37 DIV and **d** representative Tbr1 (green) staining along with MAP2b (red) and DAPI (blue) at both DIVs by Ctr-Q33 hiPSC-derived neuronal cultures. Triangular arrowheads point intense Tbr1 + neurons and spiky arrowheads point low Tbr1 + neurons. Scale bar 30 μm. (Mean ± SEM; two-way ANOVA followed by Tukey’s multiple comparison test, ns *P* > 0.05). Quantitative DAergic- and glutamatergic-related gene expression profile along (**e**–**g** and **i**) Ctr-Q33 and (**h** and **j**) GEN-Q18 neuronal differentiation. (Mean ± SEM; one-way ANOVA followed by Tukey’s multiple comparison test, relative to 0 DIV *P < 0.05, **P < 0.01, ***P < 0.001; to 8 DIV + *P* < 0.05, ++ *P* < 0.01, +++ *P* < 0.001; to 16 DIV $ *P* < 0.05, $$ *P* < 0.01, $$$ *P* < 0.001)

Concerning DAergic-TH^+^ neurons, GEN-Q18 cultures at 23 DIV contained 50 ± 2% TH^+^/Map2b^+^ neurons, a much higher population than the 19 ± 2% of Ctr-Q33 cultures (Fig. 10a). As maturation progressed, however, the proportion of TH^+^ neurons among lines reached similar values, with 36 ± 5% for Ctr-Q33 and 52 ± 4% for GEN-Q18 at 37 DIV (Fig. 10a; Tables 2 and 3). Representative immunofluorescence images for TH and MAP2b at DIV 23 and 37 DIV are shown in Fig. 10b for Ctr-Q33 and in Fig. S6a for GEN-Q18. In both cases, the length of TH^+^ projections substantially increased from 23 DIV to 37 DIV. These data were corroborated by the induction of DAergic-related genes, NR4A2 (nuclear receptor subfamily 4 group A member 2), TH and LMX1B (LIM homeobox transcription factor 1 beta) from 16 DIV onwards (Fig. 10e–g for Ctr-Q33 and Fig. 10h and i for GEN-Q18). Many of the TH^+^ neurons were also found to express GABA (Fig. S7), suggesting that a substantial proportion of TH^+^ neurons is striatal interneurons which increase TH expression as neuronal differentiation proceeds.

We also observed that hPSC-derived neuronal cultures contained a low proportion of TBR1^+^ neurons. Both cell lines exhibited less than 7% of TBR1^+^ neurons in the entire Map2b + population (Fig. 10c; Tables 2 and 3). Ctr-Q33 hiPSC-derived neuronal cultures contained 4.0 ± 0.3% of TBR1^+^ neurons at 23 DIV, which increased to 7.0 ± 0.4% at 37 DIV. In GEN-Q18 cultures, the TBR1^+^ neuronal population tended to decrease from 7 ± 3% at 23 DIV to 4 ± 1% at 37 DIV (Fig. 10c). Representative images for nuclear TBR1 staining along with Map2b at 23 DIV and 37 DIV are shown in Fig. 10d for Ctr-Q33 and in Fig. S8b for GEN-Q18. Gene expression analysis in Ctr-Q33 and GEN-Q18 cultures showed an upregulation of the glutamatergic, TBR1 at 16 DIV, shown previously in Fig. 5 and Fig. S4. Additionally, differentiated hPSCs upregulated the sodium-dependent inorganic phosphate cotransporter, SLC17A6 or VGLUT1 gene from 16 DIV onwards (Fig. 10j and k, for Ctr-Q33 and GEN-Q18, respectively).

### Striatal Projection Neurons Are Primarily Observed at 37 DIV

**Fig. 11:**
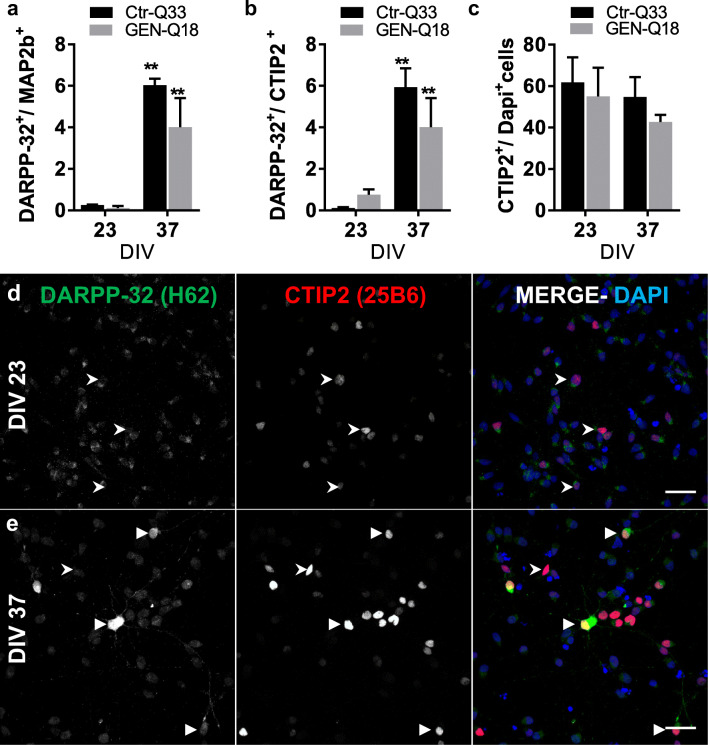
Quantification of **a** DARPP-32+/ MAP2b+, **b** CTIP2+ and **c** double DARPP-32+/CTIP2+ in Ctr-Q33 (black) and GEN-Q18 (grey) hPSC-derived cultures at 23 and 37 DIV (Mean ± SEM; two-way ANOVA followed by Tukey’s multiple comparison test, *P < 0.05, **P < 0.05). Representative images for DARPP-32 (green) and CTIP2 (red) at **d** 23 DIV and **e** 37 DIV (Arrowhead points out double-labelled neurons and arrow single-labelled-CTIP2 nucleus). Scale bar 30 μm

We specifically checked the generation of GABAergic MSNs by immunolabelling for CTIP2 (25B6) and DARPP-32 (Fig. 11 and Tables 2 and 3). Ctr-Q33 and GEN-Q18 neuronal cultures clearly exhibited DARPP-32^+^/Map2b^+^ neurons at 37 DIV, and without strong differences among cell lines (6.0 ± 0.3% and 4.0 ± 1.4%, respectively; Fig. 11a). Conversely, the differentiation of hPSC generated abundant CTIP2 (25B6)^+^/Map2b^+^ neurons at 23 and 37 DIVs. Ctr-Q33 cultures contained 62 ± 12% and 55 ± 10% of CTIP2^+^ cells at 23 DIV and 37 DIV, respectively (Fig. 11c). GEN-Q18 neuronal cultures contained 55 ± 14% at 23 DIV and 43 ± 3% of CTIP2^+^ cells at 37 DIV (Fig. 11b). Both cell lines contained equivalent proportions of double-labelled DARPP-32^+^ and CTIP2^+^ neurons (Fig. 11c). Representative staining at both DIVs in Ctr-Q33 hiPSC-derived neuronal cultures are shown in Fig. 11d and e and Fig. S8a and b in GEN-Q18.

**Fig. 12:**
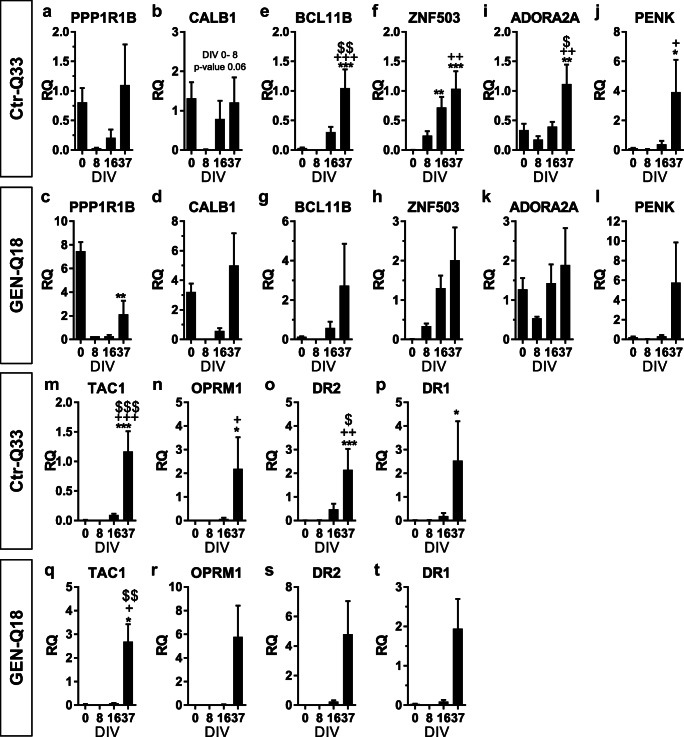
Gene expression profile of striatal MSN-related genes by Ctr-Q33 hiPSC and GEN-Q18. **a**–**d** PPP1R1B/DARPP-32 and CALB1, **e**–**h** BCL11B/CTIP2 and ZNF503, **i**–**l** ADORA2A and PENK and **m**–**t** TAC, OPRM1 and dopamine receptors DR1 and DR2 along. Mean ± SEM; one-way ANOVA followed by Tukey’s multiple comparison test, relative to 0 DIV *P < 0.05, **P < 0.01, ***P < 0.001; to 8 DIV + *P* < 0.05, ++ *P* < 0.01, +++ *P* < 0.001; to 16 DIV $ *P* < 0.05, $$ *P* < 0.01, $$$ *P* < 0.001

Quantitative expression of striatal-related neuronal genes was observed along the neuronal differentiation (Fig. 12). Confirming previous results from our group [19, 28], two well-known MSNs markers, PPP1R1B/DARPP-32 and CALB1, were expressed throughout the differentiation with high levels at DIV 0 and DIV 37 (Fig. 12a–d). Conversely, ZNF503 (zinc finger protein 503) and BCL11B/CTIP2 genes were induced from DIV 16 onwards (Fig. 11e–h). The neuronal differentiation promoted the expression of ADORA2A and the TAC1 (tachykinin precursor) receptors at 37 DIV (Fig. 11i–l). Other striatal-related genes, PENK (ENK), OPRM1 (μ-opioid receptor), DR1 and DR2 receptors, were detected at 37 DIV (Fig. 12m–t).

### Three Large Patterns of Spontaneous Neuronal Activity Are Displayed by Ctr-Q33 hiPSC-Derived Neurons at 37 DIV

To assess whether the physiological maturation of the cultures promoted neuronal activity, we carried out a single-cell, calcium fluorescence imaging assay at 37 DIV (Fig. S9). Since sharp increases in the fluorescence calcium signal are associated with the generation of action potentials, we could examine the capacity of the hiPSC-derived neuronal cultures to spontaneously fire. We observed that both Ctr-Q33 and GEN-Q18 cultures showed spontaneous activity. For sake of clarity, however, here we show the detailed analysis of the data for the Ctr-Q33 line only.

As a first general observation, the analysis revealed a high proportion of spontaneously active regions of interests (ROIs), by 84%. Since the neuronal cultures contained mostly pure Map2b^+^ at 37 DIV, ROIs were ascribed as neuronal somas (Fig. S9a-c). The activity profiles across neurons were asynchronous and heterogeneous (Fig. S9d), although most of the neurons exhibited bursting events, i.e. large amplitude firings that correspond to concatenated action potentials. To characterize the patterns of activity displayed by hPSC-derived neurons at DIV 37, we extracted a set of neuronal features through a customized software (NETCAL) run in MATLAB. We defined the number of spikes (NS), the inter-spike interval (ISI), the number of bursts (B), the inter-burst interval (IBI), spikes inside burst, ISI inside burst and burst length (Fig. S10).

**Fig. 13:**
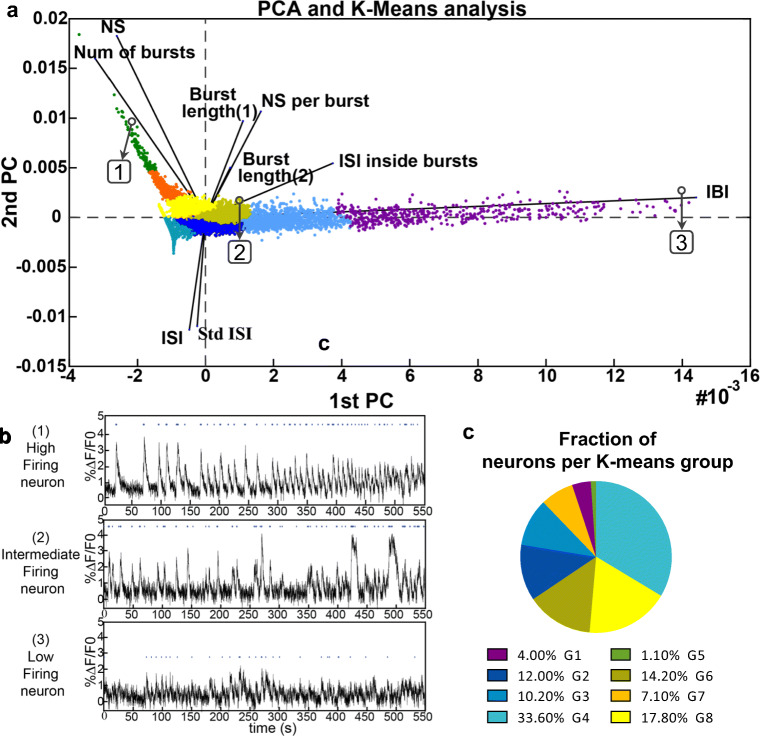
Segregation of Ctr-Q33 hiPSC-derived neurons by its spontaneous activity at 37 DIV. **a** PCA plot from active neuronal population segregated based on the neuronal features and clustered in 8 groups (differentially coloured). **b** Individual fluorescence trace (Δ*F*/*F*_0_) (*t*) from neurons 1–3 from **a** PCA with different activity patterns. **c** Pie plot of the fraction of neurons in each group (G1–G8)

About 12,000 hPSC-derived neurons were analysed. The different patterns of spontaneous activity were segregated by analysing the set of neuronal features through PCA and k-means algorithms (Fig. 13a). Each of the neuronal features displayed different relative contributions to the PCA axes (termed PC1 and PC2) as shown in Fig. S10. As a central result of the classification, PC1 mostly conveyed the IBIs feature, with a relative contribution of 84%, while the PC2 mostly conveyed he NS feature, with a relative contribution of 79% (Fig. 13a). Thus, neurons close to the PC1 axis and distant from the origin (0,0) effectively exhibited high IBI values and low NS. This characteristic ensemble of neurons was classified as “low firing neurons” (Fig. 13a). Conversely, neurons near and above the PC2 axis (Fig. 13a) elicited abundant spikes but portrayed low IBI and were classified as “high firing neurons”. In between these phenotypes, there existed a group of neurons clustered at the centre of the PCA plot and that was classified as “intermediate firing neurons” (Fig. 13a). Illustrative neuronal traces for the different classifications are shown in Fig. 13b.

Figure S11, together with S12, details the neuronal properties of each group, including the firing and bursting features. Neurons from groups G5 and G8 displayed greater firing activity compared with neurons of groups G1–G4. Neurons from group G5 elicited abundant spikes (mean of 320 ± 75 spikes per neurons), a high frequency of 0.5 Hz and a low ISI of 1.91 s. Neurons from G7, G8 and G6 also showed high spontaneous activity with a mean of 182 ± 27, 112 ± 18 and 79 ± 16 spikes, respectively. Neurons included in these groups fired spikes at relative high frequency values in the range 0.1–0.3 Hz and, therefore, low ISI of 5–8 s (Fig. S11a–c). Conversely, neurons from G1 to G4 groups (60% of the neurons) elicited a low number of spikes (< 60 spikes per neurons), at low frequencies < 0.1 Hz and high ISI > 10s (Fig. S11a–c).

Practically all neurons, regardless the group, exhibited bursting features. The only exception was group G4, in which about 50% of the neurons were non-bursting. We thus concluded that differentiation of the Ctr-Q33 line procured intrinsically bursting neurons. However, bursting patterns were not uniform (Fig. S11d–h, i). Groups G5 and G7 (8% of the population) displayed strong bursting activity with about 40–60 bursts along the recording time, which provided a typical IBI of 10–16 s (Fig. S11d and e). Neurons from groups G1 to G3, together with the bursting neurons of G4 (totalling 60% of the population), showed a weak bursting activity with a mean of only 1–7 bursts and intervals greater than 40 s (Fig. S11d and e). The remaining groups G6 and G7 (32% of the population) displayed intermediate bursting values, in the range 7–17 bursts per neurons that provided IBIs in the range 27–60 s.

Concerning burst structure (Fig. S11f–h), Ctr-Q33 neurons in general fired a mean of 2 spikes per neuron within a burst, and with an interval of 500–800 ms. Groups G5 and G7 were an exception, and showed more spikes at lower intervals. These groups also displayed slightly longer bursts with a mean duration of 8.6 and 7.7 s, respectively (Fig. S11h). For statistic comparisons of each feature between groups, see Fig. S13.

Putting together this group classification with the PCA analysis outlined above, we note that the “low firing neurons” correspond to groups G1 to G4 and comprise 60% of the neurons (Fig. S13). The “intermediate firing neurons” correspond to groups G6 and G8 and comprise 32% of neurons (Fig. S14). The “highly firings neurons” are the remaining groups G5 and G7 and comprise 8% of the neurons (Fig. S15). Thus, the differentiation of the Ctr-Q33 line not only led to spontaneously active neurons but also orchestrated a network in which different kinds of activity patterns could be observed.

### Ligand-Gated Receptors Are Expressed by Ctr-Q33-Derived Neurons at 37 DIV

**Fig. 14:**
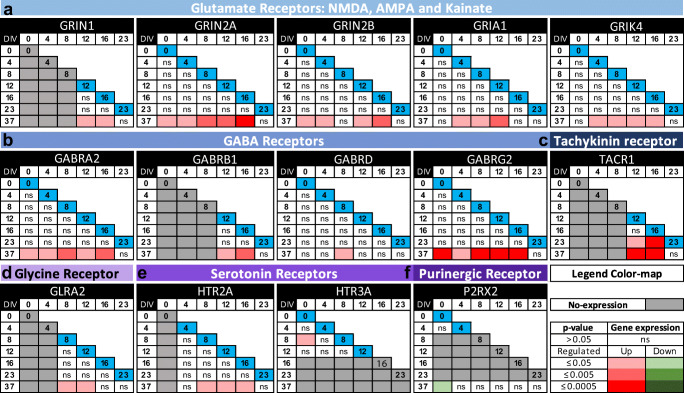
Quantitative gene expression matrices of ligand ion-gated receptors expression by Ctr-Q33-derived cultures along the neuronal differentiation (DIV vs. DIV). **a** Glu, **b** GABA, **c** tachykinin, **d** glycine, **e** serotoninergic and **f** purinergic receptors along Ctr-Q33 hiPSC neuronal differentiation. One-way ANOVA followed by Tukey’s multiple comparison test was performed between DIVs (0 to 37). Coloured matrices represent all possible comparisons on the diagonal (blue box; DIV on which the statistical test is done for all the multiple comparisons); non-expressing genes are represented as grey boxes. Colour boxes are expressing genes with *P* < 0.05. Upregulated and downregulated genes are represented by red and green respectively with increasing three colour-scale intensities for *P* ≤ 0.5, *P* ≤ 0.005 and *P* ≤ 0.0005 respectively

We performed a screening of several NT receptors by quantitative gene expression analysis along Ctr-Q33 hiPSC neuronal differentiation (Fig. 14). Regarding the excitatory Glu receptors, the three main NMDRAs subunits, GRIN1 (NR1), GRIN2A (NR2A) and GRIN2B (NR2B), and the AMPA subunit GRIA1 (GluR11) were induced by Ctr-Q33 hiPSC-derived cultures at DIV 37 (Fig. 14a). GRIN2D (NR2D) and GRIN3B (NR3B) along with other AMPA receptor subunits were expressed through the differentiation but they did not show significant changes in expression (Fig. S17a). GRIA2 subunit was found from DIV 8 onwards but the expression remained similar along all DIVs (Fig. S17b). For kainate receptors, Ctr-Q33 neurons upregulated the expression of GRIK4 (KA-1) at DIV 37 compared with 4–16 DIVs (Fig. 13a). GRIK3/4/5 gene expression was detected along differentiation, but with similar values across DIVs (Fig. S17). GRIK1 was detected from DIV 4 onwards but without changes (Fig. S17b).

Ctr-Q33 neurons upregulated the expression of GABR-A2/B1/D and G2 genes at 37 DIV (Fig. 14b). Also, neurons started to express the GPCRs tachykinin receptor TACR1 (NK1R) at 12 DIV and afterwards it became upregulated at 23 DIV and 37 DIV (Fig. 14c). The Gly receptor subunit GLRA2 was induced at 37 DIV compared with 8 DIV and 12 DIV (Fig. 14d). Ctr-Q33 neurons expressed the excitatory serotonergic 5-HT receptors, Na^+^-K^+^ 5-HT channel HTR2A and Gq/G11-GPCRs HTR3A (Fig. 14e). The HTR2A receptor was detected from 4 DIV onwards and it was induced at 37 DIV. Conversely, HTR3A gene was expressed at the beginning of differentiation up to 12 DIV with an upregulation peak at 8 DIV. The differentiation of Ctr-Q33 hiPSCs resulted in the downregulation of the excitatory ATP-gated P2RX2 gene at 37 DIV compared with 0 DIV (Fig. 14f).

### Neuronal Functional Maturation of hiPSC-Derived Neurons Is Facilitated by the Expression of K+, Na+, Ca2+ and Cl-ion Channels

We also analysed the expression of voltage-gated (VG) and ion-gated channels along Ctr-Q33 hiPSC neuronal differentiation (Fig. S16). The differentiation of Ctr-Q33 hiPSCs promoted the upregulation of the VGNC SCN2B (Navβ2) at 37 DIV. Conversely, the SCN4A (Nav1.4) was downregulated at 8 and 12 DIVs compared with 0 DIV, and the SCN8A was downregulated at 8 DIV compared with 37 DIV (Fig. S16a).

The neuronal differentiation of Ctr-Q33 hiPSCs also downregulated the expression of VG-Cl^−^ channels CLCN1 and the electroneutral K^+^/Cl^−^ cotransporter SLC12A4/KCC1 genes (Fig. S16b and c, respectively). The neuron-specific K^+^-Cl^−^ cotransporter 2 KCC2 (SLC12A5) gene was detected in Ctr-Q33 neurons along all DIVs but it did not experience changes on gene expression (Fig. S17).

In addition, the neuronal differentiation of Ctr-Q33 hiPSCs promoted the expression of several VG-K^+^ channels including (1) the A-type rapid inactivating KCNA4 (Kvα1.4); (2) the slowly/non-inactivated delayed rectifier KCNA1 (Kv1.1) and KCNH1 (Kv10.1); (3) the inward rectifiers KCNH2 (Kv11.1); and (4) the ATP-sensitive KCNJ11 (Kir6.2). All four subunits were induced at 37 DIV (Fig. S16c). Moreover, the resting membrane potential (RMP)-related KCKK2 subunit was upregulated at the progenitor’s stage at 16 DIV compared with 0–12 DIV, and the KCNK5 subunit was downregulated at 16 DIV compared with 0 DIV (Fig. S16c). Concerning VGCC, Ctr-Q33 neurons displayed higher expression of (N)-CACNA1B (Cav2.2) and L-type CACNA1C (Cav1.2) genes at 23 DIV and 37 DIV compared with 0 DIV to 16 DIV. The β subunits CACNB4 and the calcium/calmodulin-dependent protein CAMK2A genes were also induced at 37 DIV (Fig. S16e). Ctr-Q33 neurons also expressed the CAMK1G, CAMK4 and CASK genes but their levels were unaltered along differentiation (Fig. S17a).

### Mouse Striatal Environmental Cues Promote the Differentiation of hNPCs to Striatal Projection Neurons at 3 Months Post-Transplantation

**Fig. 15:**
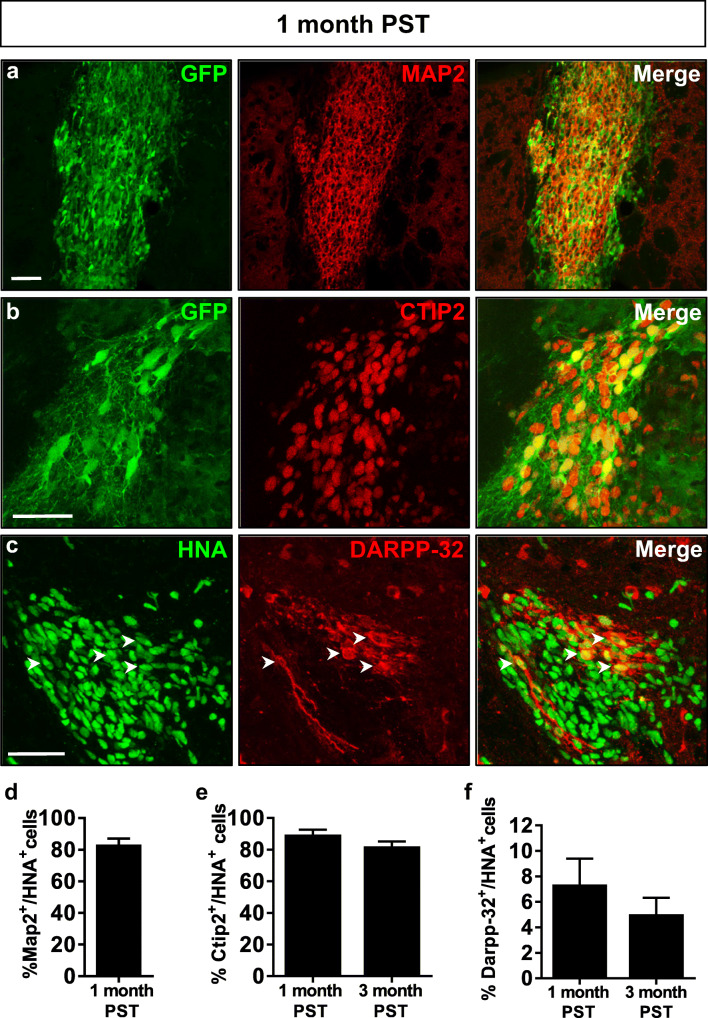
hNPCs transplanted into the mouse neonatal striatum differentiate into striatal neurons. Pictures of Ctr-Q33-GFP hiPSC-derived neurons at 1 month PST labelled with GFP or HNA (green) and **a** MAP2, **b** Ctip2 or **c** DARPP-32 (red). Cell counts for MAP2 **d** at 1 month PST and CTIP2 **e** and DARPP-32 **f** at 1 and 3 months PST. Scale bars 50 μm

**Fig. 16:**
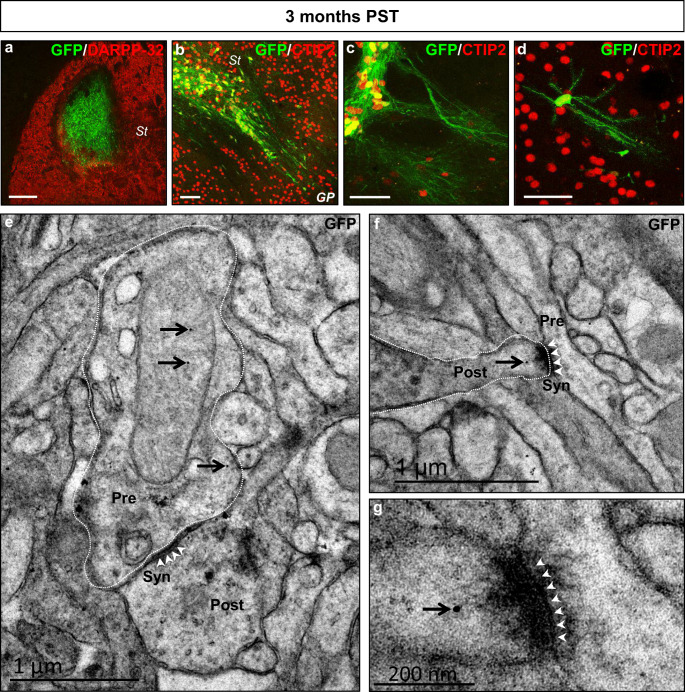
Transplanted hNPC-derived neurons project towards MSN targets and establish synaptic connections at 3 months post-transplantation. **a** Low-magnification picture of a coronal section of the mouse striatum showing grafted Ctr-Q33-GFP human cells at 3 months PST. **b**–**d** Sagittal sections of the mouse striatum showing CTIP2^+^ human cells extending GFP^+^ projections towards the external globus pallidus area. **e**–**g** Ultra-thin sections at the mouse striatum-globus pallidus level showing TEM images of human neurites (dotted lines), identified by means of GFP immunogold labelling (black arrows). **e** Human neurite (pre) establishing a symmetric inhibitory synapse (white arrowheads) with a host mouse cell (post). **f** Human neurite (post) receiving an asymmetric excitatory synapse (white arrowheads) from a host mouse cell (pre). **g** High magnification of the postsynaptic density of the synapse (white arrowheads) illustrated in **f**. *St* striatum; *GP* globus pallidus; *Pre* presynaptic; *Post* postsynaptic; *Syn* synapse. Scale bars 200 μm in **a**; 50 μm in **b**, **c**, **d**

Finally, we investigated the differentiation potential of hPSC-derived NPCs in vivo. We conducted a number of experiments in which GFP-expressing Ctr-Q33 hiPSC-derived NPCs at 16 DIV were transplanted into the neonatal mouse striatum (Fig. S19a). We evaluated the differentiation and integration of grafted human cells at short-term (1 month) and long-term (3 months) post-transplantation (PST) time points. A total of 18 neonatal mice were transplanted with one dying from complications related to surgery, while the other 17 mice reached adulthood without any significant alterations being observed (94% survival). In the 17 surviving mice, we observed no sign of teratoma formation in any animal. At 1 month PST, Ctr-Q33-GFP cells showed good survival (total number of HNA^+^ cells, 3948 ± 195; % surviving cells, 26 ± 1%; *n* = 6). They were mainly located within the core of the graft and differentiated into striatal neurons, as they were labelled for MAP2, CTIP2 and DARPP-32 (Fig. 15a–c). Cell counts (Fig. S19c) revealed a high proportion of MAP2^+^ cells (80 ± 4%) at 1 month PST (Fig. 15d). Remarkably, 89 ± 3% of transplanted hNPCs showed CTIP2 nuclear staining and 7 ± 2% expressed DARPP-32 (Fig. 15e and f, respectively). By 3 months PST, the percentage of Ctr-Q33-GFP-derived neurons positive for these markers was slightly reduced, with 82 ± 5% of CTIP2^+^ cells and 5 ± 1% of DARPP-32^+^ cells (Fig. 15e and f). At this time point, the average graft volume was 91 ± 4 × 10^6^ μm^3^ (Fig. 16a and Fig. S19b) and human cells located outside the core of the graft reflected increased migration (Fig. 16d, Fig. S19b and Fig. S20c). Most importantly, Ctr-Q33-GFP hNPCs-derived neurons were able to project long axons towards the external globus pallidus, a native striatum target (Fig. 16b and c and Fig. S20a and b). Electron microscopy analysis of these projections by means of GFP immunogold labelling revealed numerous synaptic connections between host and grafted neurons, indicating that functional integration of the transplanted cells had occurred (Fig. 16e–g). Human neurons established symmetric (inhibitory) synapses (Fig. 16e) and received asymmetric (excitatory) synaptic inputs (Fig. 16f and g) from host cells, mimicking MSN circuitry within the basal ganglia. It is of note that of the population of HNA^+^ cells found in the mouse striatum, 76 ± 5% maintained GFP expression at 3 months PST.

### Robustness of the Protocol

At the time of revision of this manuscript, this neuronal differentiation protocol has been performed a total of 399 times, 165 times with hESC lines (41%) and 234 times with hiPSC lines (59%). The protocol has been conducted by different staff members of our centre, including PhD students, technicians and post-doctoral researchers. Although differentiation was not deeply characterized on all occasions as described here, on 349 occasions (87%), a mature neuronal phenotype was achieved, while failure only occurred on 50 occasions (13%). These results demonstrate the robustness of the protocol.

## Discussion

The neurogenesis of hPSCs is a complex process comprised by several phases and signals. This includes the neural fate specification of hPSCs, the proliferation and specification of NPCs to region-specific progenitors and their terminal differentiation to subtype-specific neurons. The neuronal differentiation is accompanied by neurites and axon outgrowth, synapse formation and maturation that finally shape the neuronal circuit. Here we modelled the in vitro differentiation of two hPSC lines from pluripotency to forebrain mature neurons. The neuronal differentiation generated enriched-ventral telencephalic neuronal progenitors which showed a successful in vitro functional maturation and in vivo integration.

### Patterning of hPSCs to Telencephalic Neural Progenitors

The initial stages of the differentiation protocol rapidly and efficiently induced a neuroectodermal identity and by 8 DIV neuroepithelial rosettes, the in vitro two-dimensional equivalent of the neural tube had formed. This is faster than with other hPSC neural differentiation protocols where 10–14 days are required for rosettes to form from hPSCs. For example, Delli Carri and colleagues showed neural rosettes 15 days after induction [29], and Aubry and colleagues after 21–23 DIV [30].

The neuroepithelial cells were successfully specified to a forebrain NPC identity and not a more posterior identity as shown by expression of the forebrain marker FOXG1 and the absence of expression of midbrain, hindbrain and spinal cord markers. A ventral forebrain NPC fate was then acquired by the majority of cells based upon the expression of the Dlx family of transcription factors and the transcription factor EBF1 which play key roles in the specification of distinct populations of ventral telencephalic progenitors. This ventral specification was achieved solely by inhibiting dorsal BMP and Wnt signals [31, 32]. The ventralising morphogen SHH was omitted in an attempt to avoid the specification of ventral MGE-interneuron progenitors at the expense of striatal neuron progenitors [33–35]. FOXG1 expression may have contributed to the observed efficient ventral NPC specification as it is known to inhibit canonical Wnt signalling which specifies a dorsal fate. A small population of Pax6-expressing NPCs was also observed which may be suggestive of a dorsal forebrain identity [36]. Increasing slightly the concentration of the BMP and Wnt signalling inhibitors could avoid the formation of these dorsal NPCs and potentially produce a pure population of ventral NPCs. However, Pax6 is also expressed in neuroepithelial progenitors [27]. Given that these Pax6-expressing cells also expressed ventral markers and that a low number of Tbr1-expressing neurons were observed, this suggests that most of the Pax6^+^ cells are neuroepithelial progenitors.

The DLX family of transcription factors and EBF1 are involved in the specification of distinct populations of ventral NPCs and neuroblasts. The DLX family is involved in specifying MSNs of the indirect pathway MSNs while EBF1 plays a role in specifying direct pathway MSNs [37–39]. At 16 DIV, more DLX-expressing NPCs were observed than EBF1-expressing NPCs and this difference may be attributable to the different expression time windows of these transcription factors during striatal neurogenesis. Whereas DLX-1 and 2 are expressed by proliferative NPCs in the VZ and SVZ, EBF1 is expressed later by NPCs as they differentiate to postmitotic neuroblasts in the MZ [40–42]. Thus, the in vitro expression patterns of transcription factors involved in striatal development mirror the patterns observed in vivo indicating that the differentiation protocol recapitulates key aspects of neurodevelopment.

### Specification of hPSC-Derived NPCs to Postmitotic Telencephalic Neurons

The main affected area of the brain in HD is the striatum due to the degeneration of the GABAergic MSNs. A cell therapy approach to replace the degenerated MSNs is a viable strategy to treat HD and for this reason, we have developed the ventral forebrain neuron differentiation protocol described here in addition to using it as a platform to study neurodevelopment in both healthy and disease contexts.

This protocol efficiently generates neurons with almost all cells acquiring a neuronal identity by the end of the protocol. This compares favourably to other striatal neuron differentiation neurons where a mixture of cell types including neurons is often obtained [29, 30, 43]. These neurons express a wide range of ion channels, NMDA receptors and synaptic-associated genes indicating that they are functional at both the individual neuron and network level. Furthermore, these neurons display a large amount of sprouting and arborisation, as well as containing spine-like structures as has been shown previously [18]. This protocol produces neurons from hPSCs in just 37 days which is faster than other striatal differentiation protocols where longer periods of time are required (see Fig. 18). The protocol developed by Arber and colleagues [35] requires a similar period of time to acquire a neuronal phenotype (37–43 DIV). However, whereas Arber et al. demonstrate firing in a low number of neurons after 93 DIV [35], here we showed activity in 84% of the analysed neurons at 37 DIV.

MSNs are commonly defined by the co-expression of CTIP2 and DARPP-32 [29, 44]. Although a high number of CTIP2-expressing neurons were observed with this protocol, the number of neurons co-expressing the two proteins was 6%. Published MSN differentiation protocols vary both in their reproducibility and in the amount of MSNs that they produce, ranging from 4 to 60% (see Fig. S18 and references therein). Factors that may contribute to this variability include the PSC lines that are used, how those PSC lines are maintained prior to differentiation and differences between the protocols including how and when signalling pathway activity is modified. Other sources of variability include the antibodies that are used to detect DARPP-32, their sensitivity and also the isoforms of DARPP-32 that these antibodies detect. No consensus exists on which DARPP-32 antibody is the best for detecting MSNs in vitro and as such different groups use different antibodies which recognise different epitopes [28]. Different human DARPP-32 isoforms are known to exist with the isoforms that are expressed varying according to the neurodevelopmental stage. Furthermore, DARPP-32 has the potential to undergo several post-translational modifications (PTMs) depending on the state of the cell and the signals which are being received [28] which further complicates antibody-mediated detection. Taking these factors together illustrates the difficulties associated with using DARPP-32 as a robust marker for in vitro differentiated MSNs.

Given the large number of cells that express either CTIP2 or GABA, and the doubts associated with using DARPP-32 as MSN marker, we are of the opinion that there are more than 6% MSNs in the cultures. We propose that these additional MSNs either express isoforms or contain PTMs that are not detectable with the DARPP-32 antibody used here. Alternatively, DARPP-32 may be expressed at a low level that is below the detection threshold of the antibody. The hypothesis that there are more MSNs present in the cultures than are detected by the antibody is strengthened by the observed expression of several other genes that label MSNs at the later stages of the differentiation protocol.

A small percentage of glutamatergic cortical neurons was detected based on the expression of known cortical markers. This low abundance correlates with the small amount of Pax6-expressing dorsal NPCs that was observed at an earlier stage of the differentiation. We also consider the possibility that a fraction of the neurons expressing CTIP2 but not DARPP-32 is also cortical neurons as in addition to being expressed in the striatum, it is also expressed in layer 5 of the cortex [45, 46].

An abundant amount of tyrosine hydroxylase (TH)-expressing neurons was also detected. Although TH is a marker of midbrain dopaminergic neurons, in this case, it is unlikely that these neurons have this identity as during differentiation, we did not detect expression of Pax2 which is necessary for midbrain neurogenesis [47]. Furthermore, midbrain dopaminergic neuron differentiation requires SHH, FGF8 and Wnt/β-catenin signalling which are either inhibited or absent in this protocol [48]. These TH-expressing neurons are likely to be a subpopulation of striatal interneurons as during neurodevelopment, these interneurons are generated by the medial ganglionic eminence [49, 50] which is adjacent to the lateral ganglionic eminence that generates the MSNs. Gradients of the same signalling molecules are involved in generating these regions, and many of the same transcription factors are involved in the development of both of these regions [51]. From an in vitro perspective, slight variations during the patterning stage of the differentiation protocol in the concentrations of the small molecules received by individual cells and the processing of these signals could drive them to become MGE NPCs instead of LGE NPCs. The striatal interneuron identity hypothesis was confirmed when we observed co-expression of TH and GABA as has been described previously for a subpopulation of striatal interneurons [52]. This GABAergic striatal interneuron identity was further supported by the observed expression of various interneuron-related genes at the transcriptomic level at the same differentiation stage.

### Synaptic and Functional Maturation of hPSC-Derived Telencephalic Neurons

Effective in vitro neuronal differentiation protocols require the production of functionally mature neurons where gene expression profiles and functional properties mirror those of the corresponding endogenous neuronal types. Functional maturation requires the expression of a range of ion channels, receptors, intracellular signalling components and synaptic function-related genes whose expression levels may alter as maturation proceeds. The expression of ion channels, receptors and intracellular signalling components contributes to the intrinsic functional properties of individual neurons while functional neuronal network development relies upon expression of the synaptic machinery.

The expression of ligand-gated channels and VGCs contributes to the acquisition of passive and active neuronal properties, including excitability and the ability to elicit and transmit action potentials [53]. We observed upregulation of the RMP-related channel subunit, which is likely to be at least partly responsible for the negative resting membrane potential of hPSC-derived neurons that we have reported previously [18]. Furthermore, expression of voltage-gated sodium channels (VGNCs) was also observed which correlates with the ability of hPSC-derived neurons to display VGNC currents [23]. At the later stages of neuronal differentiation, we observed expression of several voltage-gated potassium channels (VGKCs) and voltage-gated calcium channels (VGCCs) that are responsible for driving functional maturation. Previous work showed that the blockage of VGCCs results in a dramatic suppression of action potentials by hPSC-derived neurons and a decline in the RMP [45]. Taken together, the expression of these ion channel-related genes contributes to neuronal electrophysiological maturation and indicates that this protocol produces functionally maturing neurons.

Neuronal differentiation and maturation also require intracellular calcium transients (iCa^2+^) and calcium-mediated signalling events. iCa^2+^ can arise from two main sources, from extracellular (eCa^2+^) influx which is triggered by growth factors or NT-mediated depolarization, or from the release of iCa^2+^ stores [54]. Increased iCa^2+^ concentration initiates downstream signalling cascades that result in modulation of gene expression [55], receptor trafficking [56] and synapse remodelling [55], among other functions. Thus, it is essential that in vitro differentiated neurons develop a functional calcium sensing and signalling system.

In most CNS neurons, Ca^2+^ influx is mediated by the opening of surface L-Type VGCCs, and also AMPA and NMDA receptors. The upregulation of such genes was observed at the later stages of our differentiation protocol. Other VGCCs, specifically the N- and P/Q-types, are highly expressed at the presynapse where they mediate Ca^2+^-dependent presynaptic NT-vesicle release to the synaptic cleft via direct interaction with SNAREs and vesicle-adapter proteins [57, 58]. In response to an incoming action potential, presynaptic VGCCs open and allow iCa^2+^ levels at the terminal buttons to rise from basal nanomolar concentrations to over 50 μM [59]. Expression of N-type VGCCs and several SNARE proteins was observed in our neuronal cultures suggesting that these neurons have functional Ca^2+^-mediated signalling pathways and are developing functional synapses. Further evidence of Ca^2+^-mediated neuronal maturation was inferred from the observed expression of the auxiliary β4-L-type VGCC. This subunit modulates the surface expression and gating of the α1-VGCC subunit, and promotes neuronal maturation by repressing the action of the heterochromatin protein 1 gamma (HP1γ) [60, 61]. β4-VGCC subunit expression at the later stages of the differentiation protocol might mediate Ca^2+^-dependent HP1γ repression and facilitate neuronal maturation. Taken together, we conclude that these neurons have functional Ca^2+^ signalling and homeostatic systems that have the potential to produce iCa^2+^ transients which are essential for neuronal maturation.

Synaptic maturation requires the expression of presynaptic-related proteins, including vesicle-associated proteins, and postsynaptic-related proteins, such as NMDARs and AMPA subunits. Such gene expression patterns were observed at the later stages of the differentiation protocol, indicating that synapse formation and maturation were occurring in our neuronal cultures which in turn would result in the formation of neuronal networks. Previously we showed that hPSC-derived neurons responded to excitatory (Glu) and inhibitory (GABA) NTs and that they evoked miniature postsynaptic excitatory and inhibitory currents in respond to Glu and GABA, respectively [18]. In addition, we showed that the hPSC-derived neurons were sufficiently mature to perform the GABA switch, where GABA evokes miniature inhibitory currents instead of excitatory currents which is characteristic of immature neurons [18]. This prior work complements the observations of synaptic maturation described here. In the present work, however, dynamic expression of the genes responsible for the GABA switch was not detected. Neither upregulation of the neuron-specific cotransporter KCC2 nor downregulation of the immature neuron-specific NKCC1 cotransporter, which is responsible for the reduction of intracellular iCl^−^ ions, was observed [62–64]. This discrepancy may be due to the fact that in this work, gene expression was studied at the neuronal population level where different neuronal types at different maturation stages are present. A population level analysis may mask the changes of gene expression associated with the GABA switch if it only occurred in a subset of the total population.

To validate the apparent functionally mature state of the neurons indicated by the gene expression studies, we performed high-speed somatic Ca^2+^ imaging at the endpoint of the protocol. We found that a high proportion of neurons displayed spontaneous iCa^2+^ transients. These transients were characterized by a fast Ca^2+^ rise followed by a slow decay, a hallmark of elicited action potentials [65]. Neuronal activity typically appeared in the form of bursts, which corresponds to trains of action potentials, although the degree of activity varied between neurons. Analysis of the spontaneous firing neuronal activity identified three main groups which varied according to their activity and were termed “high firing” (HF), “intermediate firing” (IF) and “low firing” (LF) neurons. HFNs comprised 8% of the neurons which fired strong bursting events every 10 s. In contrast, LFNs comprised 60% of the neurons, exhibited weak bursting every 40 s or more, and in general tended to elicit single spike events at low rate. The IF neurons, which comprise the remaining 32%, displayed an intermediate behaviour firing between 10 s and 40 s. LFNs are likely to be functionally immature neurons as they very rarely generated bursts of spikes which is a feature of more mature neurons.

Various reasons are likely to contribute to the variety of neuronal activity patterns observed here. As described above, different neuronal types are present within the same culture and each neuronal type is likely to have a different activity pattern and maturation rate. Furthermore, the rate of neuronal maturation is unlikely to be uniform, and thus it is probable that neurons of the same type are at different maturation stages both at the individual cell and network level which would contribute to the heterogeneous activity state. Also, given that both glutamatergic and GABAergic neurons are present in the same culture, similar cells within a culture may experience different local conditions in terms of excitatory and inhibitory inputs which would further contribute to variable activity. This protocol produces neurons in a highly efficient manner, but the absence of astrocytes could also contribute to the abundance of weakly firing neurons. Astrocytes have been shown to increase functional maturation, potentiate the activity of developing neurons and contribute to synapse formation [66–69].

At the neuronal circuitry level, we must bear in mind that endogenous striatal neurons receive excitatory Glu inputs from the cortex and thalamus and DA inputs from the substantia nigra pars compacta. It has been shown that the inhibition of cortico-striatal projections during synaptogenesis alters MSNs maturation by reducing dendritic spine density [67]. The addition of such inputs could have a positive impact on in vitro striatal neuron differentiation and network formation. Thus, the development of a differentiation protocol with integrated cortical and dopaminergic inputs could have a positive impact on in vitro striatal neuron differentiation and network formation.

Based on the relevant gene expression and functional data, in combination with previously published data [18, 19], we conclude that the differentiation protocol produces functional neurons with gene expression patterns similar to those observed during functional neuronal maturation in vivo.

### Striatal Neuronal Differentiation of hPSC-Derived NPCs Within the Mouse Striatum and Integration into Host Circuitry

The suitability of this protocol for use as part of cell therapy-based treatments was evaluated by the transplantation of hPSC-derived NPCs into the neonatal mouse striatum. In the ideal scenario, which is fully compatible with a cell therapy-based approach, transplanted NPCs should differentiate to the desired neuronal type without excessive proliferation. These neurons should then survive for a prolonged period and integrate into the host circuitry.

The transplanted NPCs differentiated to neurons within 1 month of grafting, with no increased number of transplanted cells being observed by 3 months post-transplantation. This demonstrates that the transplanted NPCs integrate local signals to exit the cell cycle and commit to a neuronal identity with a minimal risk of graft overgrowth. This compares favourably to previous work where excessive graft overgrowth was observed following the transplantation of hPSC-derived NPCs into mouse striatum [29, 30]. The majority of the NPC-derived neurons acquired a striatal identity based on the expression of known striatal neuron markers, which correlates well with the in vitro neuronal gene expression data. These neurons also showed higher survival and terminal differentiation in the mouse striatum compared with other studies [68].

A subset of the neurons displayed polarised innervation towards the external globus pallidus (GP) as occurs with endogenous MSNs, indicating that they are integrating into the relevant host circuitry. During development, axon outgrowth and projection are highly regulated by cell-autonomous mechanisms and also by extracellular cues [69, 70]. Several TFs, including CTIP2, ISL1 and EBF1, have been implicated in striatonigral MSN axon outgrowth [37, 71, 72]. Expression of these TFs was observed in hPSC-derived neurons both in vitro and post-transplantation, indicating that NPCs are correctly patterned prior to grafting. The combination of NPC intrinsic properties acquired during in vitro differentiation, together with the integration of signals from the host environment, led transplanted cells to behave in a similar manner to host striatal neurons. Furthermore, hNPC-derived neurons established inhibitory synapses (likely GABAergic) and received excitatory synaptic inputs (likely glutamatergic) from host neurons. This mirrors the input-output circuitry of MSNs and suggests that hNPC-derived neurons are also functional within the host circuitry. Remarkably, little evidence of architecturally appropriate neuronal integration, much less circuit reconstruction, has been reported after transplantation of hESC or hiPSC-derived MSNs into the striatum [73]. Based on the behaviour of the transplanted NPCs, we conclude that our differentiation protocol successfully meets all the criteria for use in cell therapy-based strategies.

## Conclusion

Here we perform an in-depth characterisation of an in vitro differentiation protocol that produces functional ventral forebrain neurons, including medium spiny neurons, from hPSCs in just 37 days. We observe that the protocol is robust and reproducible, and that the dynamic changes in gene expression that occur during the protocol mirror those observed during human neurodevelopment. The neurons that are produced in vitro are functionally mature while the protocol is also compatible with cell therapy-based approaches following the successful transplantation of hPSC-derived NPCS which undergo neuronal differentiation and integrate into the host brain circuitry.

Given all of these characteristics, we conclude that this protocol is an ideal platform for the in vitro study of both neurodevelopment and neuronal diseases, including those neurodegenerative disorders that cause alterations during neurogenesis. Due to its robust, reproducible and rapid nature, it is also ideal for use as a drug screening and developmental toxicology platform. Finally, we believe that this protocol has great potential as the basis of cell therapy-based strategies to treat neurodegenerative diseases where there is currently no cure.

## Electronic Supplementary Material

ESM1 (PDF 8.37 mb)
