# Supplementary material for: Human Pluripotent Stem Cell-Derived Neurons Are Functionally Mature In Vitro and Integrate into the Mouse Striatum Following Transplantation
Source: Mol Neurobiol. 2020 Apr 30;57(6):2766–98. doi: 10.1007/s12035-020-01907-4 (PMC7253531; doi:10.1007/s12035-020-01907-4)
Supplement: Supplementary file 1 — (PDF 8.37 mb) [file 12035_2020_1907_MOESM1_ESM.pdf]

Supplementary material

Human Pluripotent Stem Cell-derived Neurons are Functionally Mature *in vitro* and Integrate into the Mouse Striatum *in vivo*

Andrea Comella-Bolla[1,2,3,4,5] · Javier G. Orlandi[6,7,8] · Andrés Miguez[1,2,3,4,5] · Marco Straccia[1,2,3,4,5] · María García-Bravo[9,10] · Georgina Bombau[1,2,3,4,5] · Mireia Galofré[1,2,3,4,5] · Phil Sanders[1,2,3,4,5] · Jordi Carrere[1,2,3,4,5] · José Carlos Segovia[9,10] · Joan Blasi[11] · Nicholas D. Allen[12] · Jordi Alberch[2,3,4,5,6] · Jordi Soriano[7,13] · Josep M. Canals [1,2,3,4,5].

[1]Laboratory of Stem Cells and Regenerative Medicine, Department of Biomedicine, Faculty of Medicine and Health Science, University of Barcelona, Barcelona, Spain.
[2]Production and validation center of advanced therapies (Creatio), Faculty of Medicine and Health Science, University of Barcelona, Spain.
[3]Institute of Neurosciences, University of Barcelona, Barcelona, Spain.
[4]Networked Biomedical Research Centre for Neurodegenerative Disorders (CIBERNED), Spain.
[5]August Pi i Sunyer Biomedical Research Institute (IDIBAPS), Barcelona, Spain
[6]Pathophysiology of Neurodegenerative Disease. Laboratory, Department of Biomedicine, Faculty of Medicine and Health Science, University of Barcelona, Barcelona, Spain.
[7]Departament de Física de la Matèria Condensada, University of Barcelona, Barcelona, Spain.
[8]Department of Physics and Astronomy, University of Calgary, Calgary, Canada.
[9]Differentiation and Cytometry Unit. Division of Hematopoietic Innovative Therapies, Centro de Investigaciones Energéticas Medioambientales y Tecnológicas and Centro de Investigación Biomédica en Red de Enfermedades Raras (CIEMAT/CIBERER). Madrid. Spain.
[10]Advanced Therapies Unit, Instituto de Investigación Sanitaria Fundación Jiménez Díaz (IIS-FJD, UAM), Madrid, Spain.
[11]Laboratory of Cellular and Molecular Neurobiology, Department Pathology and Experimental Therapeutics, Faculty of Medicine and Health Science, Biomedical Research Institute of Bellvitge (IDIBELL); University of Barcelona, Barcelona, Spain.
[12]Cardiff Repair Group, School of Biosciences and medicine, Cardiff University, Cardiff, Wales, UK.
[13]Institute of Complex Systems (UBICS), University of Barcelona, Barcelona, Spain.

Table S1 Table of correspondence of human gene names with human protein names

Gene	Protein name	Protein symbol
ACTB	Actin, cytoplasmic 1/ Beta-actin	Actine
ADAM10	Disintegrin and metalloproteinase domain-containing protein 10	ADAM 10
AMPH	Amphiphysin	AMPH
BDNF	Brain-derived neurotrophic factor	Bdnf
BIN1	Myc box-dependent-interacting protein 1	BIN1
CACNA1A	Voltage-dependent P/Q-type calcium channel subunit alpha-1B	Cav2.1
CACNA1B	Voltage-dependent P/Q-type calcium channel subunit alpha-1A	Cav2.2
CACNA1C	Voltage-dependent L-type calcium channel subunit alpha-1C	CaV1.2
CACNA1H	Voltage-dependent T-type calcium channel subunit alpha-1H	Cav3.2
CACNA1S	Voltage-dependent L-type calcium channel subunit alpha-1S	Cav1.1
CACNB1	Voltage-dependent L-type calcium channel subunit beta-1	CAB1
CACNB2	Voltage-dependent L-type calcium channel subunit beta-2	CAB2
CACNB3	Voltage-dependent L-type calcium channel subunit beta-3	CAB3
CACNB4	Voltage-dependent L-type calcium channel subunit beta-4	CAB4
CACNG2	Voltage-dependent calcium channel gamma-2 subunit/ Transmembrane AMPAR regulatory protein gamma-2	TARP gamma-2
CAMK1G	calcium/calmodulin-dependent protein kinase IG	CaMKIG
CAMK2A	calcium/calmodulin-dependent protein kinase II alpha	CAMKA
CAMK4	calcium/calmodulin-dependent protein kinase IV	CAMK4
CASK	Peripheral plasma membrane protein CASK	CASK
CLCA1	Chloride channel accessory 1	hCaCC-1
CLCN1	Voltage-sensitive Chloride channel protein 1	ClC-1
CLCN2	Voltage-sensitive Chloride channel protein 2	ClC-2
CLCN5	Voltage-sensitive Chloride channel protein 5	ClC-5
CLCN6	Voltage-sensitive Chloride channel protein 6	ClC-6
CLCN7	Voltage-sensitive Chloride channel protein 7	ClC-7
CLIC1	Chloride intracellular channel 1	NCC27
CLIC2	Chloride intracellular channel 2	XAP121
CREB1	cAMP responsive element binding protein 1	CREB
DLG1	Discs large homolog 1	SAP-97
DLG2	Discs large homolog 2	PSD-93
DLG3	Discs large homolog 3	SAP102
DLG4	Discs large homolog 4	PSD95
DRD1	Dopamine receptor D1	DRD1A
DRD2	Dopamine receptor D2	D2DR
DRD3	Dopamine receptor D3	D3DR
EIF2B1	Translation initiation factor eIF-2B subunit alpha/ Eukaryotic translation initiation factor 2B subunit 1 alpha	EIF2BA
GABRA1	gamma-aminobutyric acid (GABA) A receptor alpha 1	GABRalfa1
GABRA2	GABA (A) receptor alpha 2	GABRalfa2
GABRB1	GABA (A) receptor beta 1	GABR beta1
GABRB2	GABA (A) receptor beta 2	GABR beta2
GABRD	GABA (A) receptor delta	GABR delta
GABRE	GABA (A) receptor epsilon	GABR epsilon
GABRG1	GABA (A) receptor gamma 1	GABR gamma1
GABRG2	GABA (A) receptor gamma 2	GABR gamma2
GABRP	GABA (A) receptor pi	GABR pi
GABRQ	GABA (A) receptor theta	GABR theta
GAP43	Neuromodulin	GAP43
GLRA1	glycine receptor alpha 1	GLR alfa1
GLRA2	glycine receptor alpha 2	GLR alfa2
GLRA3	glycine receptor alpha 3	GLR alfa3

Gene	Protein name	Protein symbol
GRIA1	Glutamate receptor ionotropic, AMPA 1	GluR1/GluA1
GRIA2	Glutamate receptor ionotropic, AMPA 2	GluR2/GluA2
GRIA3	Glutamate receptor ionotropic, AMPA 3	GluR3/GluA3
GRIA4	Glutamate receptor ionotropic, AMPA 4	GluR4/GluA4
GRIK1	Glutamate receptor ionotropic, kainate 1	EAA3/GLUR5/GluK1
GRIK3	Glutamate receptor ionotropic, kainate 3	EAA5/GLUR7/GluK3
GRIK4	Glutamate receptor ionotropic, kainate 4	EAA1/GRIK/GluK4
GRIK5	Glutamate receptor ionotropic, kainate 5	EAA2/GRIK2/GluK5
GRIN1	Glutamate receptor ionotropic, N-methyl D-aspartate (NMDA) 1	GluN1/NMDAR1/NR1
GRIN2A	Glutamate receptor ionotropic, NMDA 2A	GluN2A/NMDAR2A/NR2A
GRIN2B	Glutamate receptor ionotropic, NMDA 2B	GluN2B/NMDAR2B/NR2B
GRIN2C	Glutamate receptor ionotropic, NMDA 2D	GluN2C/NMDAR2C/NR2C
GRIN2D	Glutamate receptor ionotropic, NMDA 2C	GluN2D/NMDAR2D/NR2D
GRIN3A	Glutamate receptor ionotropic, NMDA 3A	GluN3A/NMDAR3A/NR3A
GRIN3B	Glutamate receptor ionotropic, NMDA 3A	GluN3B/NMDAR3B/NR3B
GRINA	Glutamate receptor ionotropic, NMDA -associated protein 1	NMDARA1/TMBIM3
HPRT1	hypoxanthine phosphoribosyltransferase 1;hCG15412 Celera Annotation	HPRT
HTR1B	5-hydroxytryptamine (serotonin) receptor 1B	5-HT1B
HTR2A	5-hydroxytryptamine (serotonin) receptor 2A	5-HT2A
HTR3A	5-hydroxytryptamine (serotonin) receptor 3A	5-HT3A
HTR3B	5-hydroxytryptamine (serotonin) receptor 3B	5-HT3B
KCNA1	Potassium voltage-gated channel (shaker-related) subfamily A member 1	Kv1.1
KCNA10	Potassium voltage-gated channel (shaker-related) subfamily A member 10	Kv1.8
KCNA2	Potassium voltage-gated channel (shaker-related) subfamily A member 2	KV1.2
KCNA3	Potassium voltage-gated channel (shaker-related) subfamily A member 3	KV1.3
KCNA4	Potassium voltage-gated channel (shaker-related) subfamily A member 4	KV1.4
KCNA5	Potassium voltage-gated channel (shaker-related) subfamily A member 5	KV1.5
KCNAB1	Potassium voltage-gated channel subunit beta-1	Kvb1.3
KCNB1	Potassium voltage-gated channel (Shab-related) subfamily B member 1	KV2.1
KCNC1	Potassium voltage-gated channel (Shaw-related) subfamily C member 1	KV3.1
KCNC4	Potassium voltage-gated channel (Shaw-related) subfamily C member 4	KV3.4
KCND1	Potassium voltage-gated channeL (Shal-related) subfamily D member 1	KV4.1
KCND2	Potassium voltage-gated channeL (Shal-related) subfamily D member 2	KV4.2
KCNE1	Potassium voltage-gated channeL (Isk-related family) subfamily E member 1	MinK
KCNF1	Potassium voltage-gated channeL subfamily F member 1	KV5.1/kH1
KCNG1	Potassium voltage-gated channeL subfamily G member 1	KV6.1/kH2
KCNH1	Potassium voltage-gated channeL (eag-related) subfamily H member 1	Kv10.1
KCNH2	Potassium voltage-gated channeL (eag-related) subfamily H member 2	Kv11.1
KCNJ1	ATP-sensitive inward rectifier potassium channel 1/Potassium inwardly-rectifying channel subfamily J member 1	KIR1.1/ROMK1
KCNJ11	ATP-sensitive inward rectifier potassium channel 11/ Potassium inwardly-rectifying channel subfamily J member 11	KIR6.2
KCNJ4	Inward rectifier potassium channel 4	Kir2.3/IRK-3
KCNJ5	G protein-activated inward rectifier potassium channel 4	Kir3.4/IRK-4
KCNJ6	G protein-activated inward rectifier potassium channel 2	Kir3.2/KATP-2
KCNK10	Potassium channel subfamily K member 10/ Outward rectifying potassium channel protein TREK-2	K2p10.1/TREK2
KCNK2	Potassium channel subfamily K member 2/ Outward rectifying potassium channel protein TREK-1	K2p2.1/TREK1
KCNK3	Potassium channel subfamily K member 3/ Acid-sensitive potassium channel protein TASK-1	K2p3.1/TASK-1
KCNK4	Potassium channel subfamily K member 4/ TWIK-related arachidonic acid-stimulated potassium channel protei	K2p4.1/TRAAK
KCNK5	Potassium channel subfamily K member 5 / Acid-sensitive potassium channel protein TASK-2	K2p5.1/TASK-2
KCNK9	Potassium channel subfamily K member 9/ Acid-sensitive potassium channel protein TASK-3	K2p9.1/TASK-3

Gene	Protein name	Protein symbol
KCNMB1	Potassium large conductance calcium-activated channel subfamily M beta-1	K(VCA)beta-1
KCNN1	Potassium intermediate/small conductance calcium-activated channel subfamily N member 1	KCa2.1/SK1
KCNN2	Potassium small conductance calcium-activated channel subfamily N member 2	KCa2.2/SK2
KCNN3	Potassium small conductance calcium-activated channel subfamily N member 3	KCa2.3;/SK3
KCNN4	Potassium small conductance calcium-activated channel subfamily N member 4	KCa3.1/SK4
KCNQ1	Potassium voltage-gated channel subfamily KQT member 1	KvLQT1/Kv7.1
KCNQ2	Potassium voltage-gated channel subfamily KQT member 2	KvLQT2/KV7.2
KCNQ3	Potassium voltage-gated channel subfamily KQT member 3	KvLQT3/KV7.3
KCNQ4	Potassium voltage-gated channel subfamily KQT member 4	KvLQT4/KV7.4
KCNS1	Potassium voltage-gated channel delayed-rectifier subfamily S member 1	KV9.1
KCNS2	Potassium voltage-gated channel delayed-rectifier subfamily S member 2	KV9.2
KCNS3	Potassium voltage-gated channel delayed-rectifier subfamily S member 3	KV9.3
NEUROD1	Neuronal differentiation 1/ Class A basic helix-loop-helix protein 3	NEUROD/bHLHa3
NTF3	Neurotrophin 3/ Nerve growth factor 2	NT3/NGF2
NTRK1	Neurotrophic tyrosine kinase receptor type 1	TRK1
NTRK2	Neurotrophic tyrosine kinase receptor type 2	TRKB
NTRK3	Neurotrophic tyrosine kinase receptor type 3	TRKC
P2RX2	P2X purinoceptor 2	P2X2
P2RX4	P2X purinoceptor 4	P2X4
P2RX7	P2X Purinergic receptor ligand-gated ion channel	P2X7
P2RY1	P2Y Purinergic receptor G-protein coupled	P2Y1
PDE1B	Calcium/calmodulin-dependent 3',5'-cyclic nucleotide phosphodiesterase 1B	PDES1B
PPP1R9A	Neurabin-I/ Protein phosphatase 1 regulatory subunit 9A	Neurabin-I/ NRB1
PPP1R9B	Neurabin-2/ Spinophilin/ Protein phosphatase 1 regulatory subunit 9B	Neurabin-II/ Spinophilin
SCN1A	Sodium voltage-gated channel type I alpha subunit	Nav1.1
SCN2B	Sodium voltage-gated channel type II beta subunit	SCN2B
SCN3A	Sodium voltage-gated channel type III alpha subunit	Nav1.3
SCN4A	Sodium voltage-gate channel type IV alpha subunit	Nav1.4
SCN5A;-	Sodium voltage-gate channel type V alpha subunit	Nav1.5
SCN7A	Sodium voltage-gate channel type VII alpha subunit	Nav2.1
SCN8A	Sodium voltage-gate channel type VIII alpha subunit	Nav1.6
SCN9A	Sodium voltage-gate channel type IX alpha subunit	Nav1.7
SLC12A1	Solute carrier family 12 member 1/Na-K-Cl cotransporter 2	SLC12A1/NKCC2
SLC12A2	Solute carrier family 12 member 2/ Na-K-Cl cotransporter 1	SLC12A2/NKCC1
SLC12A4	Solute carrier family 12 member 4/ Electroneutral potassium-chloride cotransporter 1	SLC12A4/KCC1
SLC12A5	Solute carrier family 12 member 5/ Electroneutral potassium-chloride cotransporter 2	SLC12A5/KCC2
SLC17A8	Solute carrier family 17 member 8 /vesicular glutamate transporter 3	VGLUT3
SLC32A1	Solute carrier family 32 member 1/ GABA vesicular transporter	VGAT
SLC37A1	Glucose-6-phosphate exchanger/ Solute carrier family 37 member 1	G3PP
SLC5A11	Sodium/myo-inositol cotransporter 2/Solute carrier family 5 member 11	SMIT2
SNAP25	Synaptosomal-associated protein 25kDa	SNAP25
SV2C	Synaptic vesicle glycoprotein 2C	SV2C
SYN1	Synapsin I	SYN1
SYNGR2	Synaptogyrin 2	SYNGR2
SYNJ2	Synaptojanin 2	SYNJ2
SYNPO	Synaptopodin	SYNPO
SYP	Synaptophysin	SYP
SYT12	Synaptotagmin XII	SytXII
SYT2	Synaptotagmin II	SytII
SYT5	Synaptotagmin V	SytV
SYT6	synaptotagmin VI	SytVI
TACR1	Tachykinin receptor 1/ Substance-P receptor	SPR/NK-1R
TACR2	Tachykinin receptor 2/ Substance-K receptor/ Neurokinin A receptor	SKR/NK-2R
VAMP2	Vesicle-associated membrane protein 2/ Synaptobrevin 2	SYB2/VAMP-2
VAMP5	Vesicle-associated membrane protein 5/ Myobrevin	VAMP-5

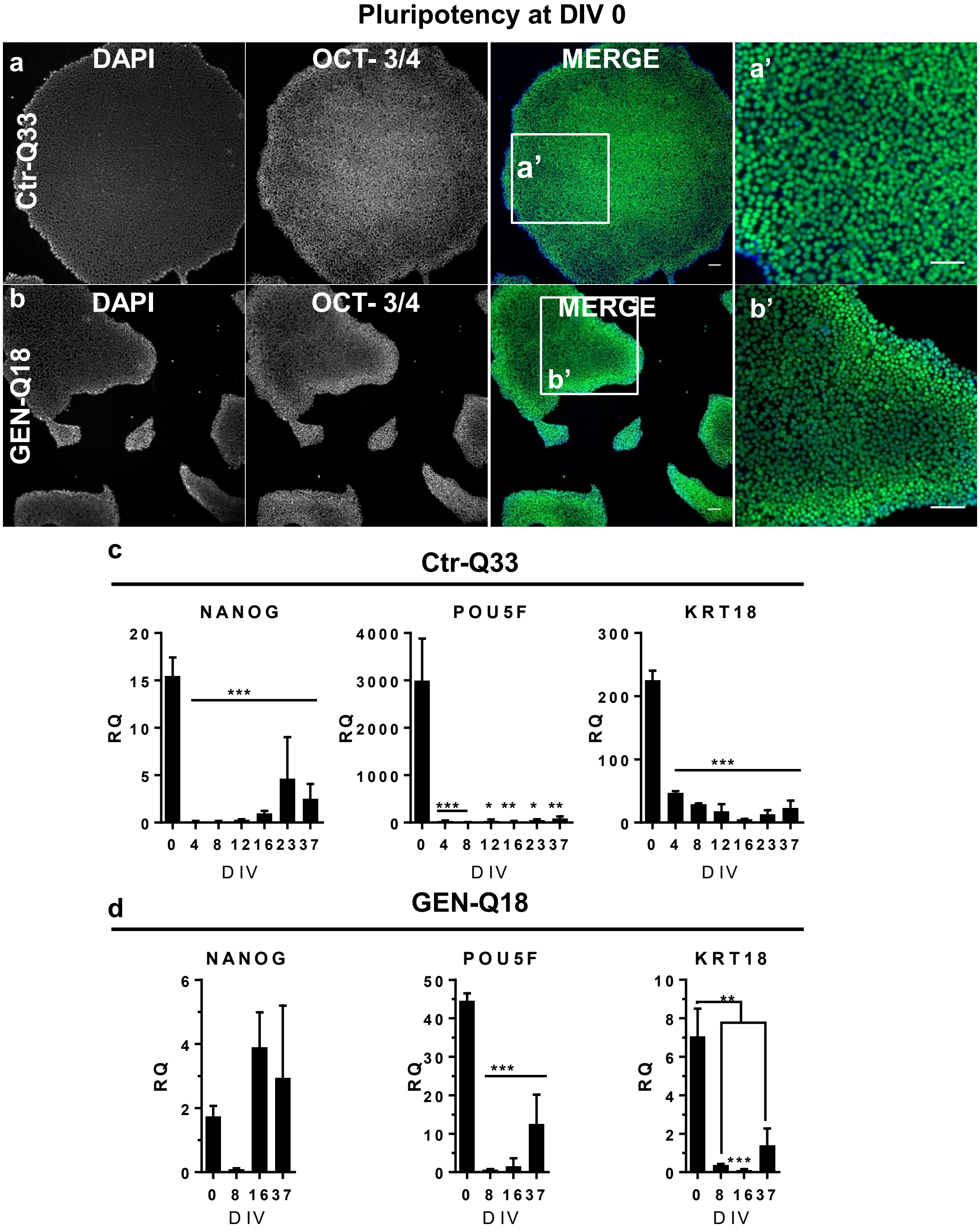

Fig. S1 Representative OCT- 3/4 immuno-fluorescence in (a) Ctr-Q33 hiPSC and (b) GEN-Q18 hESC cultures at DIV 0. The square boxes (a' and b') show a detail of the images. Scale bar is 100 µm. (c)-(d) Quantitative gene expression profiles of NANOG, POU5F1 and KRT18 mRNA levels along Ctr-Q33 (c) and GEN-Q18 (d) neuronal differentiation. Data is shown as mean ± SEM. Statistics: One-way ANOVA followed by Tukey's multiple comparison test, relative to DIV 0. *$P < 0.05$; **$P < 0.001$; ***$P < 0.0001$.

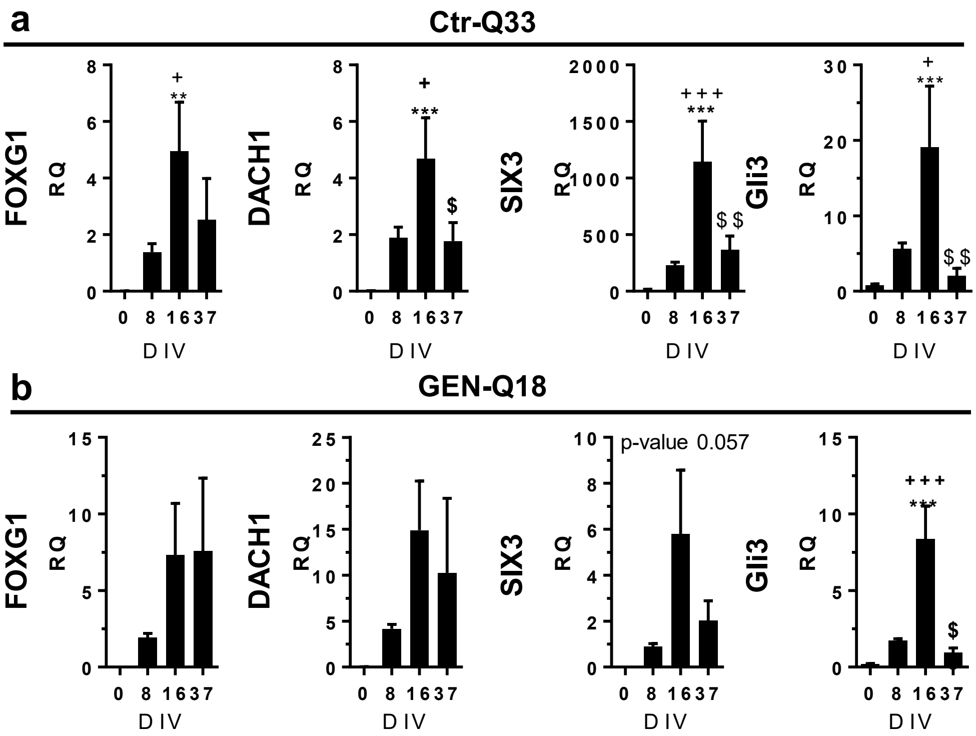

Fig. S2 Quantitative gene expression profiles of anterior telencephalic genes FOXG1, DACH1, SIX3 and GLI3 throughout the neuronal differentiation of (a) Ctr-Q33 and (b) GEN-Q18. Data is shown as mean ± SEM; one-way ANOVA followed by Tukey's multiple comparison test, relative to DIV 0, for * $P < 0.05$, ** $P < 0.01$, ***$P < 0.001$; relative to DIV 8 for + $P < 0.05$, ++ $P < 0.01$, +++ $P < 0.001$; and relative to DIV for is $$ $P < 0.01$, $$$ $P < 0.001$.

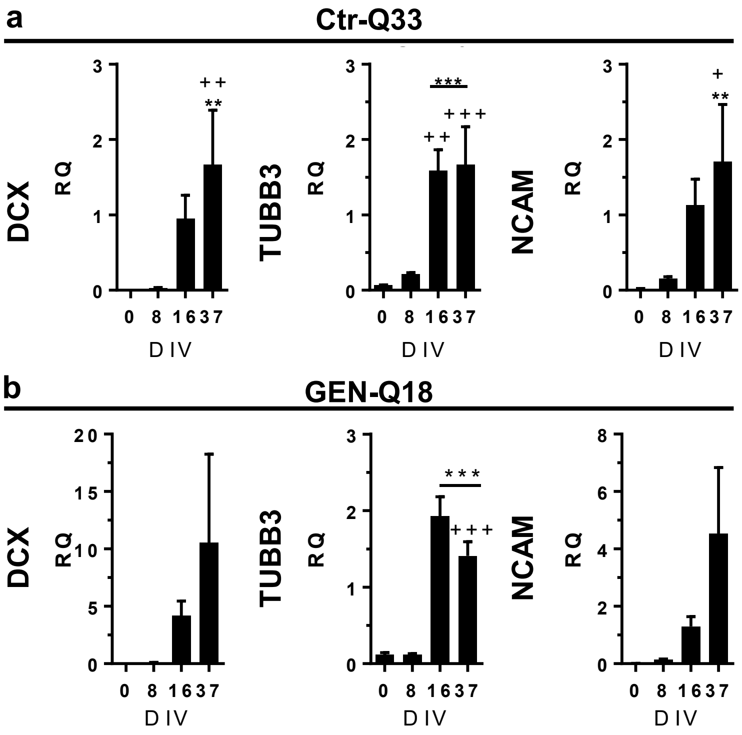

Fig. S3 Quantitative expression profile of DCX, TUBB3 and NMCA1 along (a) Ctr-Q33 and (b) GEN-Q18 neuronal differentiation. Data is shown as mean ± SEM; One-way ANOVA followed by Tukey's multiple comparison test, to DIV 0 for * $P < 0.05$, ** $P < 0.01$, ***$P < 0.001$, and to DIV 8 for + $P < 0.05$, ++ $P < 0.01$, +++ $P < 0.001$.

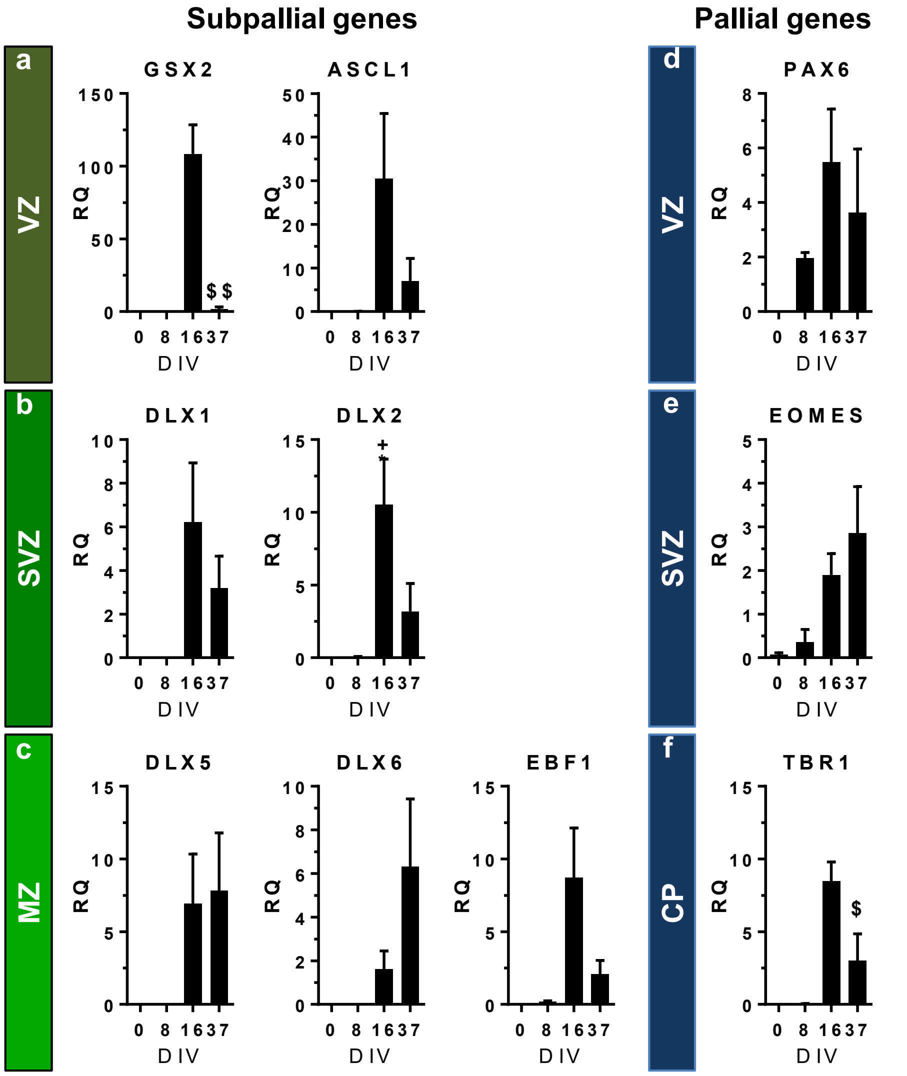

Fig. S4 Anterior telencephalic genes expression by GEN-Q18 hESC derived cultures. Quantitative gene expression of subpallial (a-c) and pallial (d-f) gene expression throughout GEN-Q18 neuronal differentiation. Data is shown as mean ± SEM; One-way ANOVA followed by Tukey's multiple comparison test, relative to DIV 0 for * P < 0.05; to DIV 8 for + P < 0.05; and to DIV 16 for \$ P < 0.05, \$\$ P < 0.01. Abbreviations: VZ: Ventricular zone; SVZ: Subventricular zone; MZ: Mantel zone and CP: Cortical plate.

GEN-Q18 hESC-derived neurons

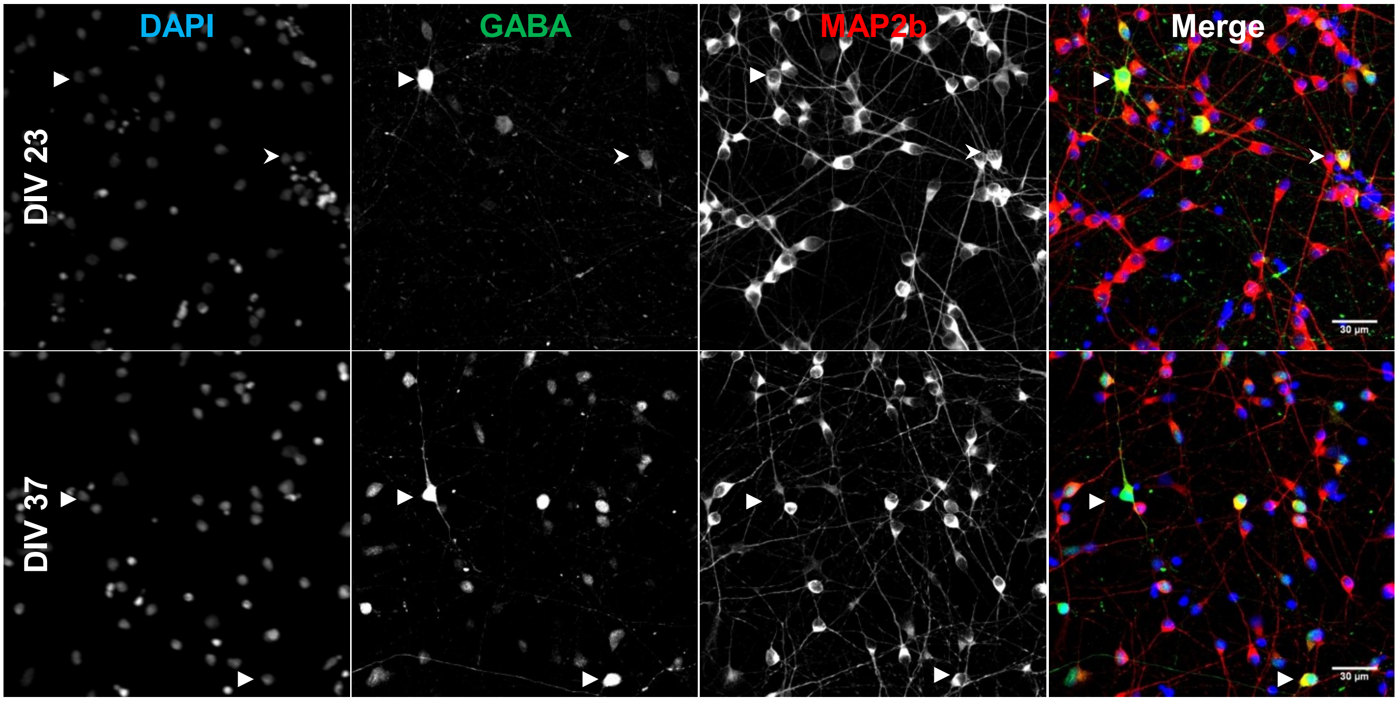

Fig. S5 GEN-Q18 hiPSC- derived GABAergic neurons at DIV 23 (top) and DIV 37 (bottom). Images show representative GABA (green) immunofluorescence along with the neuronal MAP2b (red) and DAPI (blue) at both DIV. Triangular arrowheads highlight intense GABA+ neurons; spiky arrowheads highlight low GABA+ neurons. Scale bar is 30 μm.

GEN-Q18 hESC-derived neurons

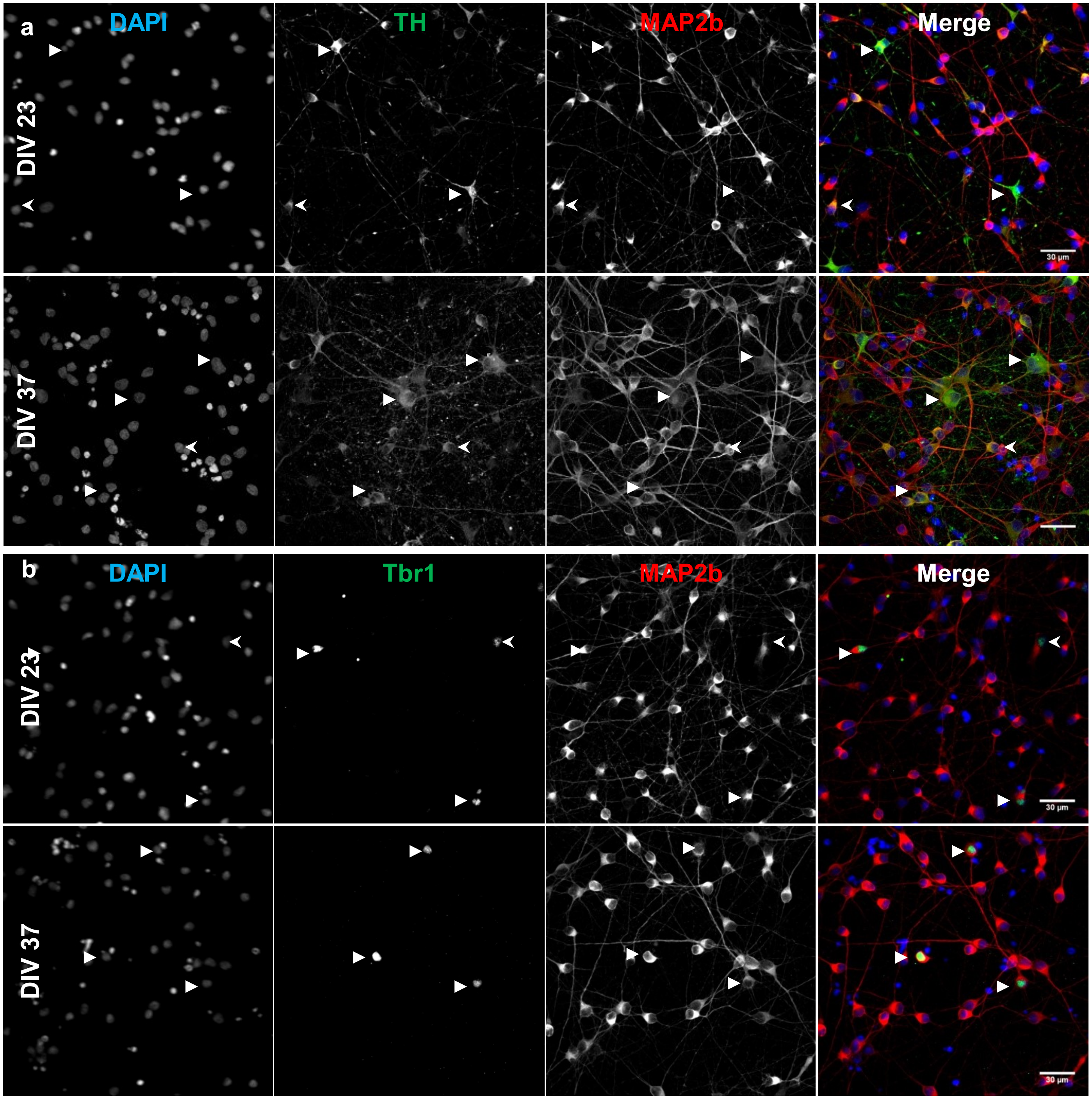

Fig. S6 GEN-Q18 hiPSC- derived TH (a) and Tbr1-expressing neurons (b) at DIV 23 and DIV 37. Images show representative TH or Tbr1 (green) staining along with MAP2b (red) and DAPI (blue). Triangular arrowheads highlight intense stained-neurons; spiky arrowheads highlight low-stained neurons. Scale bar is 30 µm.

Ctr-Q33 iPSC-derived neurons

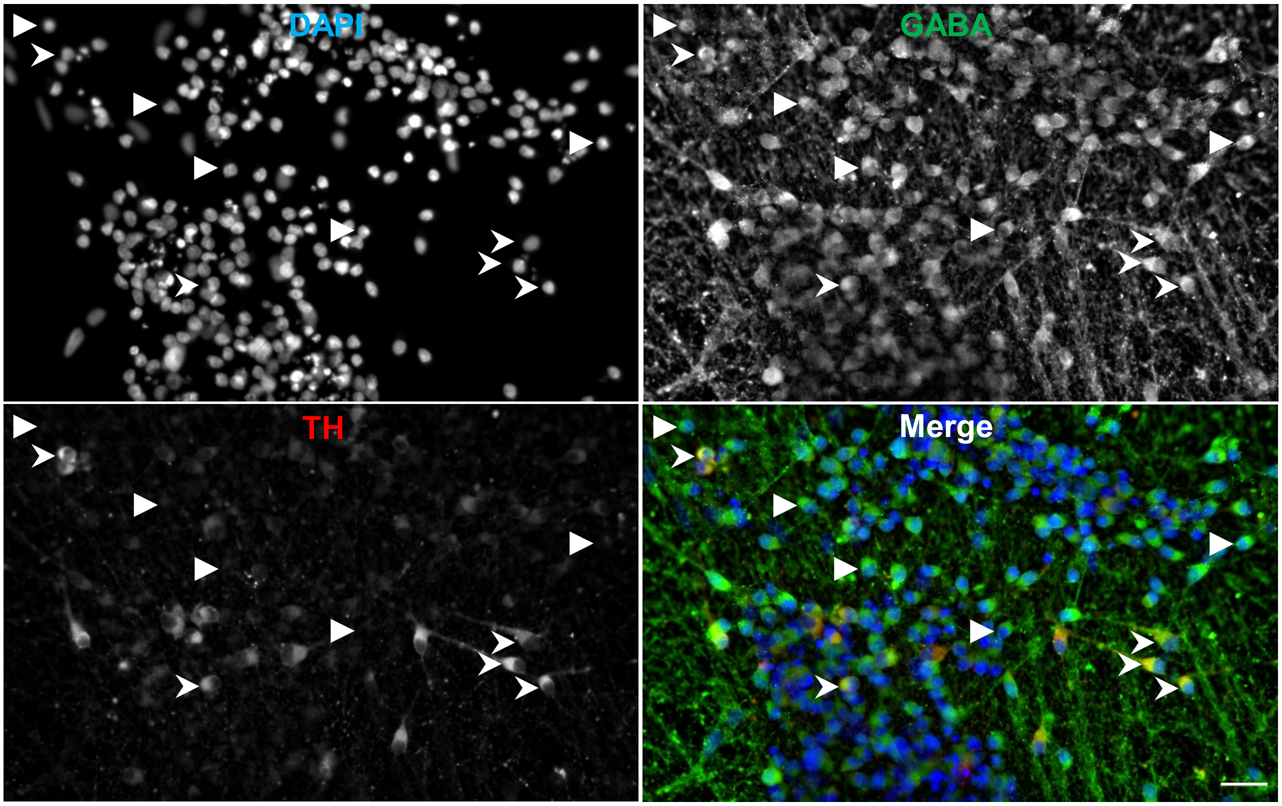

GEN-Q18 hESC-derived neurons

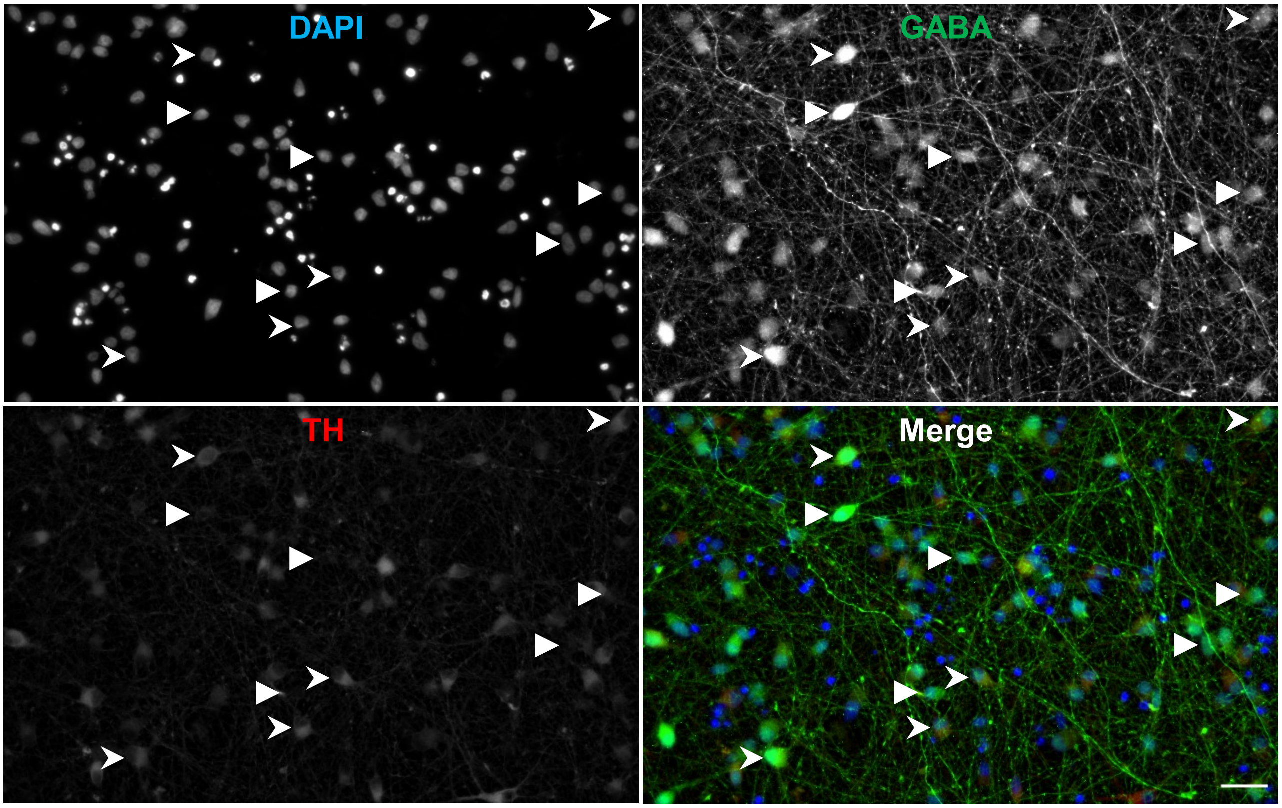

Fig. S7. Ctr-Q33 iPSC-derived (top) and GEN-Q18 hESC-derived (bottom) striatal GABAergic interneurons expressing TH at 37 DIV. Images show representative GABA (green) and TH (red) immunofluorescence with the nuclei stained with DAPI (blue). Triangular arrowheads indicate neurons expressing only GABA. Spiky arrowheads indicate neurons co-expressing GABA and TH. Scale bar is 30 µm.

GEN-Q18 hESC-derived neurons

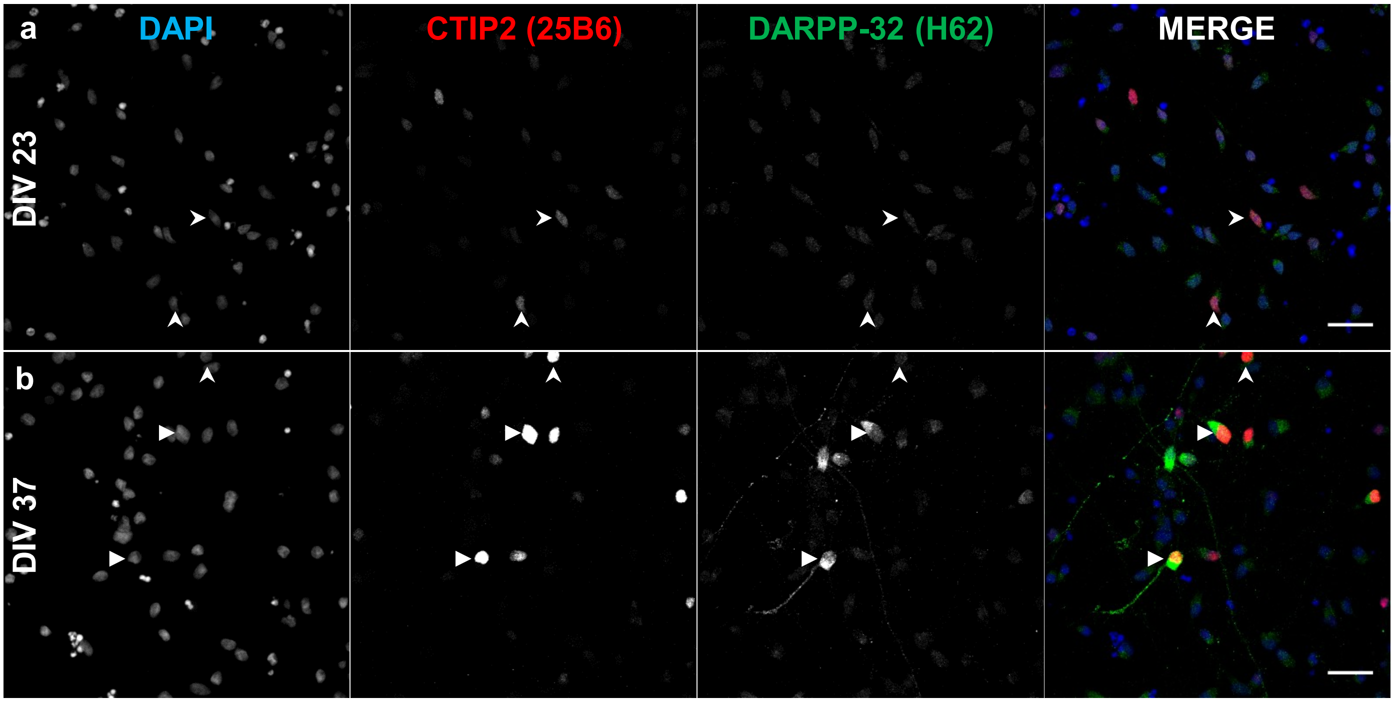

Fig. S8 Representative images for DARPP-32 (green) and CTIP2 (red) by GEN-Q18 hESC-derived cultures at (a) DIV 23 and (b) DIV 37. Triangular arrowheads highlight double labelled neurons; spiky arrowheads highlight single labelled-Ctip2 nucleus. Scale bar is 30 μm.

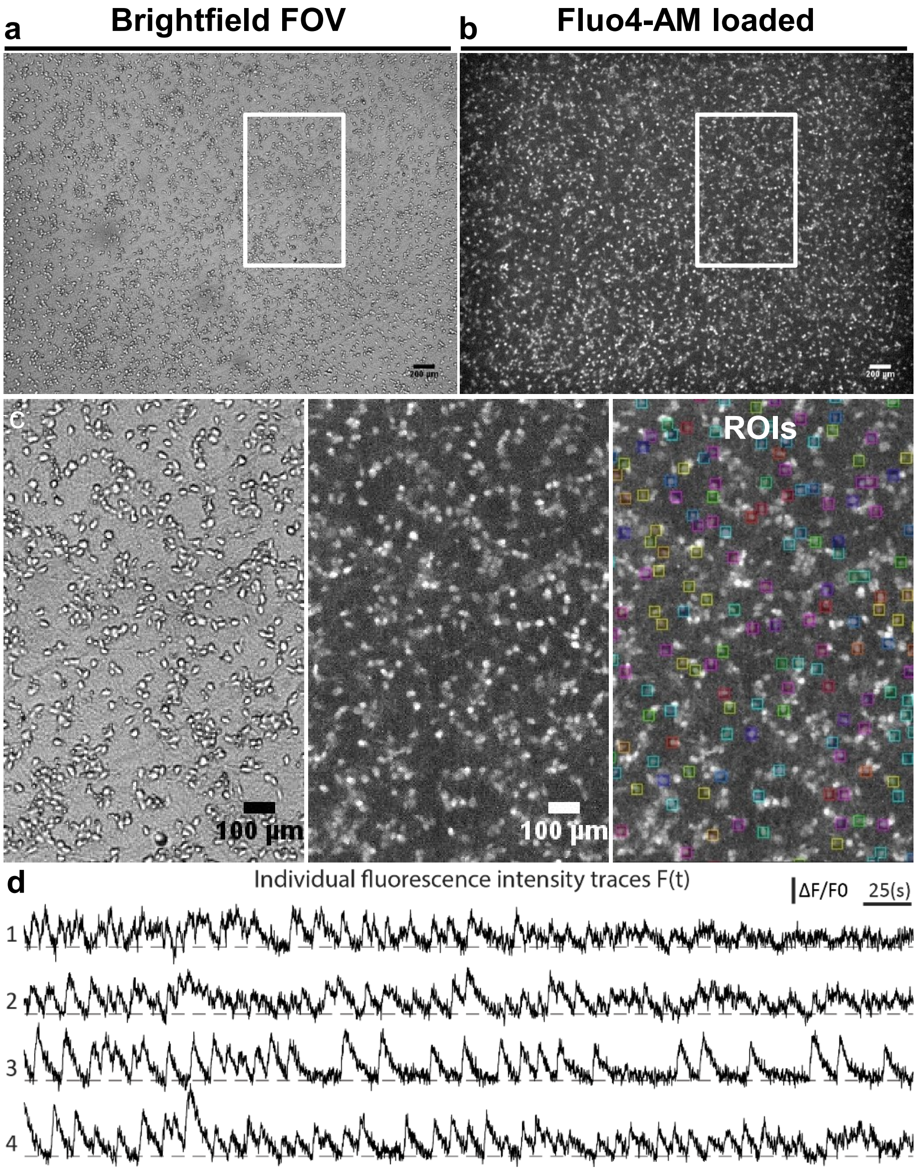

Fig. S9 Calcium imaging and single-cell fluorescence traces. (a) Brightfield and (b) Fluo4-AM loaded fluorescence images of the recorded Field of View, and for a Ctr-Q33 culture at DIV 37. (c) Magnified square regions from (a-b) to show the ability to identify single cells and ascribe them as ROIs. (d) Examples of spontaneous activity fluorescence data for single neurons as a function of time. Fluorescence is expressed as F(t)=100 (F*-F0)/F0 ≡ ΔF/F0 (%). The dashed line provides a reference for the signal baseline.

a

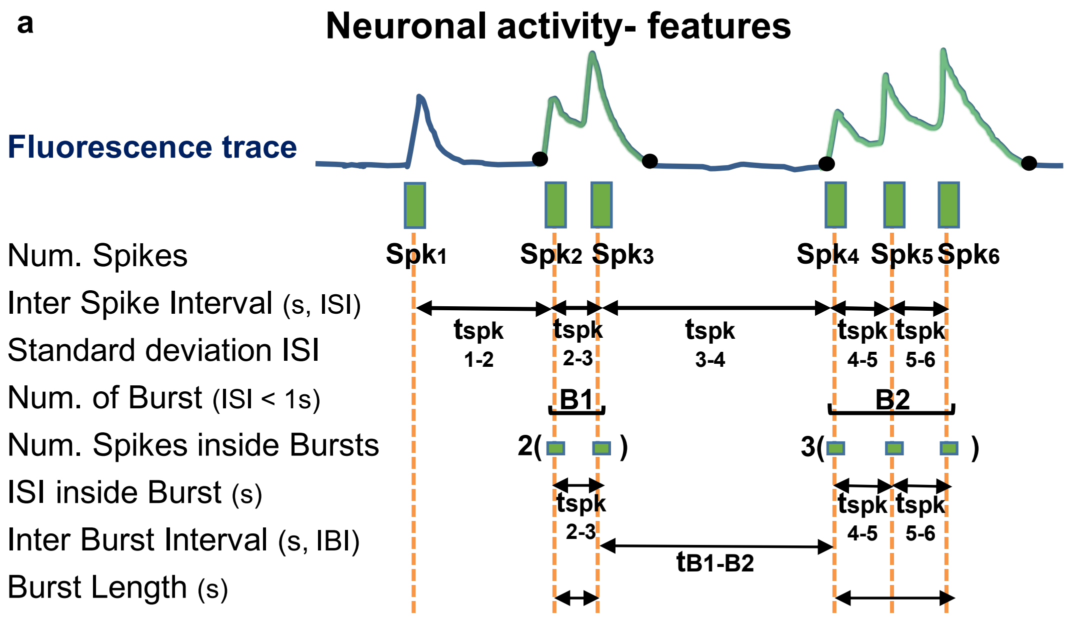

b

	Feature	PCA coefficients: PC1	PCA coefficients: PC2	Relative contribution: % PC1	Relative contribution: % PC2
1	Num of spikes	-0.14	0.95	11.5	79.4
3	Avg ISI (s)	-0.01	-0.13	0.5	10.7
4	Std ISI (s)	0.00	-0.14	0.3	11.4
5	Num of bursts	-0.04	0.20	3.4	16.6
6	Avg Num spikes per burst	0.00	0.01	0.2	1.0
7	Avg ISI inside bursts (s)	0.00	0.00	0.1	0.1
8	Avg IBI (s)	0.99	0.14	83.8	11.5
9a	Avg Burst length (s) (1)	0.00	0.00	0.0	0.4
9b	Avg Burst length (s) (2)	0.00	0.03	0.3	2.1

Fig. S10 Set of neuronal firing features, extracted for each single neuron, and that describe the activity of the hiPSC-derived neurons. (a) The sketch illustrates the definition of each feature, and corresponds to a hand-drawn fluorescence trace with six spikes-like Ca^{2+} fluctuations represented as green boxes. Lines with arrowheads indicate intervals in seconds. Spk: spike; B: bursts; t: time. (b) Features contribution to PCA relative to each feature and first and second axis.

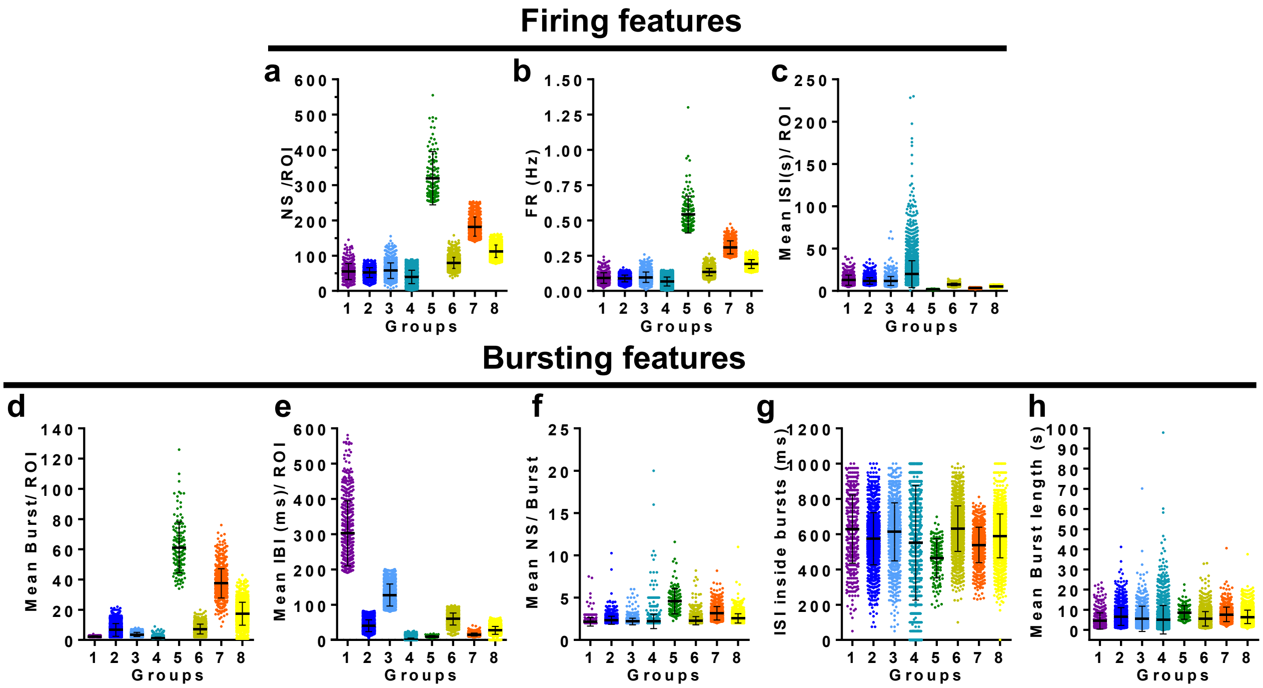

i

Number Spikes	G1	G2	G3	G4	G5	G6	G7	G8
Mean	54.8	51.9	57.5	39.6	319.6	79.5	182.4	112.6
SD	22.7	14.0	22.2	19.0	75.2	15.9	27.2	17.9
SEM	1.0	0.4	0.6	0.3	6.5	0.4	0.9	0.4

Mean Inter Burst Interval (IBI) (s)	G1	G2	G3	G4	G5	G6	G7	G8
Mean	302.4	40.6	127.4	1.3	10.1	59.5	15.5	27.2
SD	92.0	16.8	31.2	4.2	2.5	16.9	4.2	11.1
SEM	4.2	0.4	0.9	0.1	0.2	0.4	0.1	0.3

Mean Inter Spike Interval (ISI) (s)	G1	G2	G3	G4	G5	G6	G7	G8
Mean	12.7	11.7	11.7	19.8	1.9	7.6	3.3	5.3
SD	6.0	3.8	5.3	15.9	0.3	1.5	0.5	0.8
SEM	0.3	0.1	0.2	0.3	0.0	0.0	0.0	0.0

Mean Spikes inside a Burst	G1	G2	G3	G4	G5	G6	G7	G8
Mean	2.1	2.3	2.2	2.2	4.6	2.3	3.1	2.5
SD	0.5	0.5	0.4	0.8	1.5	0.5	0.8	0.5
SEM	0.0	0.0	0.0	0.0	0.1	0.0	0.0	0.0

Firing Rate (Hz)	G1	G2	G3	G4	G5	G6	G7	G8
Mean	0.09	0.09	0.10	0.07	0.54	0.13	0.31	0.19
SD	0.04	0.02	0.04	0.03	0.13	0.03	0.05	0.03
SEM	0.00	0.00	0.00	0.00	0.01	0.00	0.00	0.00

Mean ISI inside a Burst (ms)	G1	G2	G3	G4	G5	G6	G7	G8
Mean	628.9	574.0	612.9	636.0	464.3	631.4	539.1	590.2
SD	193.1	148.0	164.6	260.5	111.7	128.9	100.8	124.7
SEM	8.9	3.9	4.8	6.2	9.7	3.2	3.5	2.8

Number Burst	G1	G2	G3	G4	G5	G6	G7	G8
Mean	2.3	6.7	3.7	1.2	61.3	7.2	37.5	17.4
SD	0.5	4.2	1.3	0.7	16.8	3.3	9.7	7.9
SEM	0.0	0.1	0.0	0.0	1.5	0.1	0.3	0.2

Burst length	G1	G2	G3	G4	G5	G6	G7	G8
Mean	4.4	6.6	5.5	5.0	8.6	5.5	7.7	6.5
SD	4.0	4.4	6.2	7.1	3.4	3.6	3.6	3.4
SEM	0.2	0.1	0.2	0.2	0.3	0.1	0.1	0.1

Fig. S11 Firing and bursting features of Ctr-Q33 hiPSC-derived neurons clustered in eight characteristic groups at DIV 37. The box plots show: (a) the number of spikes (NS); (b) the frequency (FR, Hz); (c) the Inter-spike-Interval (ISI, s); (d) the number of Bursts; (e) the Inter-Burst interval (IBI, ms); (f) the number of spikes inside a burst; (g) the ISI inside a burst (ms); and (f) the burst length (s). Groups are represented with different colors. The horizontal line within the boxplot shows the mean; the vertical lines show ± SD. (i) Tables with the Mean, SD and SEM for each feature.

Num Spikes

	G1	G2	G3	G4	G5	G6	G7	G8
**G1**		ns	ns	***	***	***	***	***
**G2**	0.1507		***	***	***	***	***	***
**G3**	0.2328	< 0,0001		***	***	***	***	***
**G4**	< 0,0001	< 0,0001	< 0,0001		***	***	***	***
**G5**	< 0,0001	< 0,0001	< 0,0001	< 0,0001		***	***	***
**G6**	< 0,0001	< 0,0001	< 0,0001	< 0,0001	< 0,0001		***	***
**G7**	< 0,0001	< 0,0001	< 0,0001	< 0,0001	< 0,0001	< 0,0001		***
**G8**	< 0,0001	< 0,0001	< 0,0001	< 0,0001	< 0,0001	< 0,0001	< 0,0001	

ISI inside Burst

	G1	G2	G3	G4	G5	G6	G7	G8
**G1**		***	ns	ns	***	ns	***	***
**G2**	< 0,0001		***	***	***	***	***	ns
**G3**	0.6561	< 0,0001		**	***	ns	***	**
**G4**	0.9925	< 0,0002	0.0061		***	ns	***	***
**G5**	< 0,0001	< 0,0003	< 0,0001	< 0,0001		***	***	***
**G6**	> 0,9999	< 0,0004	0.0742	0.9929	< 0,0001		***	***
**G7**	< 0,0001	< 0,0005	< 0,0001	< 0,0001	< 0,0001	< 0,0001		***
**G8**	0.0002	0.0998	0.0055	< 0,0001	< 0,0001	< 0,0001	< 0,0001	

ISI

	G1	G2	G3	G4	G5	G6	G7	G8
**G1**		ns	ns	***	***	***	***	***
**G2**	0.4766		ns	***	***	***	***	***
**G3**	0.4229	> 0,9999		***	***	***	***	***
**G4**	< 0,0001	< 0,0001	< 0,0001		***	***	***	***
**G5**	< 0,0001	< 0,0001	< 0,0001	< 0,0001		***	***	**
**G6**	< 0,0001	< 0,0001	< 0,0001	< 0,0001	< 0,0001		***	***
**G7**	< 0,0001	< 0,0001	< 0,0001	< 0,0001	0,7927	< 0,0001		***
**G8**	< 0,0001	< 0,0001	< 0,0001	< 0,0001	0,0019	< 0,0001	< 0,0001	

Num Spikes inside Burst

	G1	G2	G3	G4	G5	G6	G7	G8
**G1**		***	ns	ns	***	**	***	***
**G2**	< 0,0001		***	***	***	ns	***	***
**G3**	0.9041	< 0,0001		ns	***	**	***	***
**G4**	0.9421	< 0,0001	> 0,9999		***	***	***	***
**G5**	< 0,0001	< 0,0001	< 0,0001	< 0,0001		***	***	***
**G6**	0.0014	0.3713	0.0052	0.0003	< 0,0001		***	***
**G7**	< 0,0001	< 0,0001	< 0,0001	< 0,0001	< 0,0001	< 0,0001		***
**G8**	< 0,0001	< 0,0001	< 0,0001	< 0,0001	< 0,0001	< 0,0001	< 0,0001	

Num Burst

	G1	G2	G3	G4	G5	G6	G7	G8
**G1**		***	***	**	***	***	***	***
**G2**	< 0,0001		***	***	***	ns	***	***
**G3**	< 0,0001	< 0,0001		***	***	***	***	***
**G4**	0,0032	< 0,0001	< 0,0001		***	***	***	***
**G5**	< 0,0001	< 0,0001	< 0,0001	< 0,0001		***	***	***
**G6**	< 0,0001	0,0773	< 0,0001	< 0,0001	< 0,0001		***	***
**G7**	< 0,0001	< 0,0001	< 0,0001	< 0,0001	< 0,0001	< 0,0001		***
**G8**	< 0,0001	< 0,0001	< 0,0001	< 0,0001	< 0,0001	< 0,0001	< 0,0001	

Burst Length

	G1	G2	G3	G4	G5	G6	G7	G8
**G1**		***	**	ns	***	**	***	***
**G2**	< 0,0001		***	***	***	**	***	ns
**G3**	0.0024	< 0,0001		ns	***	ns	***	***
**G4**	0.3459	< 0,0001	0.1472		***	ns	***	***
**G5**	< 0,0001	0.0002	< 0,0001	< 0,0001		***	ns	***
**G6**	0.0014	< 0,0001	> 0,9999	0.0914	< 0,0001		***	***
**G7**	< 0,0001	< 0,0001	< 0,0001	< 0,0001	0.4502	< 0,0001		***
**G8**	< 0,0001	0.9749	< 0,0001	< 0,0001	< 0,0001	< 0,0001	< 0,0001	

IBI

	G1	G2	G3	G4	G5	G6	G7	G8
**G1**		***	***	***	***	***	***	***
**G2**	< 0,0001		***	***	***	***	***	***
**G3**	< 0,0001	< 0,0001		***	***	***	***	***
**G4**	< 0,0001	< 0,0001	< 0,0001		***	***	***	***
**G5**	< 0,0001	< 0,0001	< 0,0001	< 0,0001		***	ns	***
**G6**	< 0,0001	< 0,0001	< 0,0001	< 0,0001	< 0,0001		***	***
**G7**	< 0,0001	< 0,0001	< 0,0001	< 0,0001	0.3061	< 0,0001		***
**G8**	< 0,0001	< 0,0001	< 0,0001	< 0,0001	< 0,0001	< 0,0001	< 0,0001	

p-value	
> 0.05	ns
≤ 0.05	*
≤ 0.01	**
≤ 0.001	***

Fig. S12 Statistic tables of firing and bursting features of the eight characteristic groups of Ctr-Q33 hiPSC-derived neurons clustered at DIV 37. Coloured matrices represent all possible comparisons on the diagonal (Blue box; Group on which the statistical test is done for all the multiple comparisons); One-ways ANOVA followed by Tukey's multiple comparison test; P < 0.05 *, P≤ 0.01 ** and P ≤ 0.001 ***. P > 0.05 (Grey boxes).

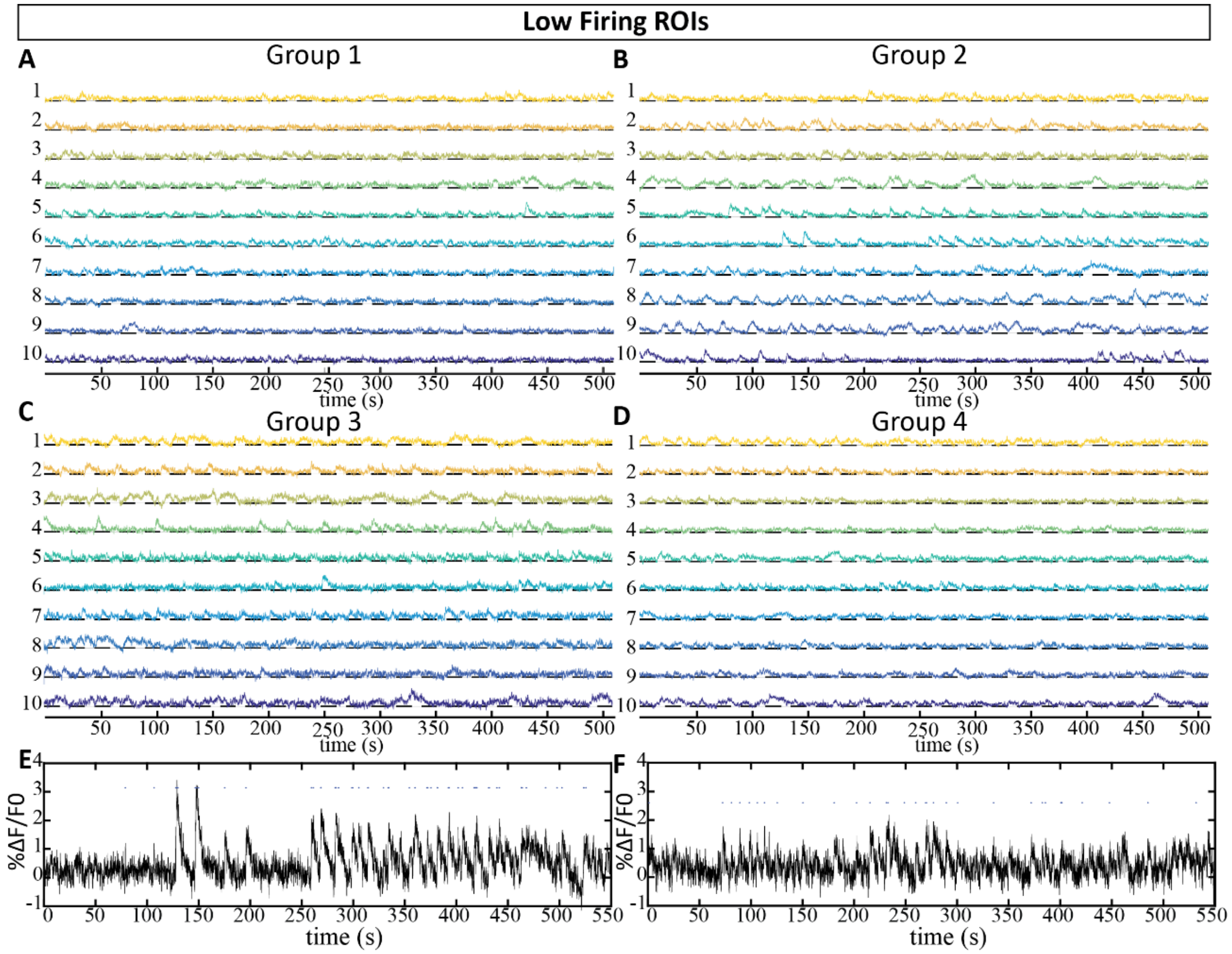

Fig. S13. Illustrative Ctr-Q33 hiPSC-derived neurons classified as ‘low firing neurons’ (LFN) at DIV 37. (a) Representative individual fluorescence traces from group G1 to G4 (a to d). All traces are equally normalized along time. (e) Magnified fluorescence trace of neuron 6 from group G2. (f) Equivalent magnification of neuron 5 from group G4. In general, neurons in these groups elicit single-spikes and short-lasting bursts. Inferred spikes in panels (e)-(f) are represented with the upper blue-dots. All fluorescence traces are normalized as ΔF/ F0.

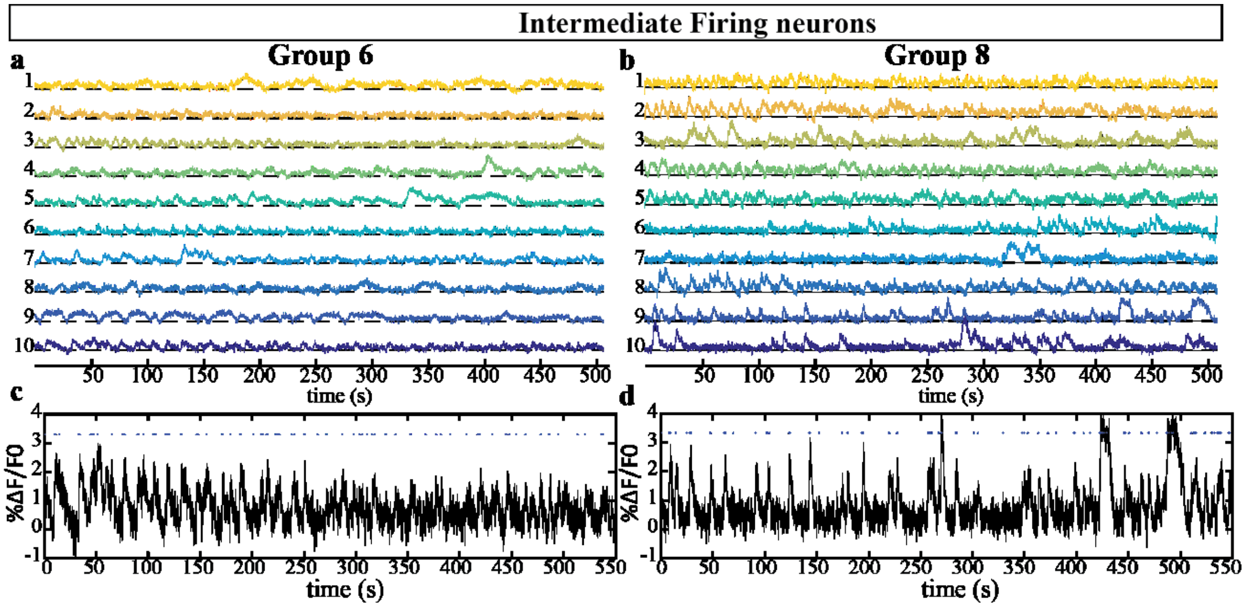

Fig. S14 Illustrative Ctr-Q33 hiPSC-derived neurons classified as ‘intermediate firing neurons’ (IFN) at DIV 37. (a)-(b) Representative individual fluorescence traces from group G6 and G8. (c) Magnified fluorescence trace of neuron 10 from group G6. (d) Equivalent magnification of neuron 9 from group G8. Inferred spikes are represented with the upper blue-dots. All fluorescence traces are normalized as ΔF/ F0.

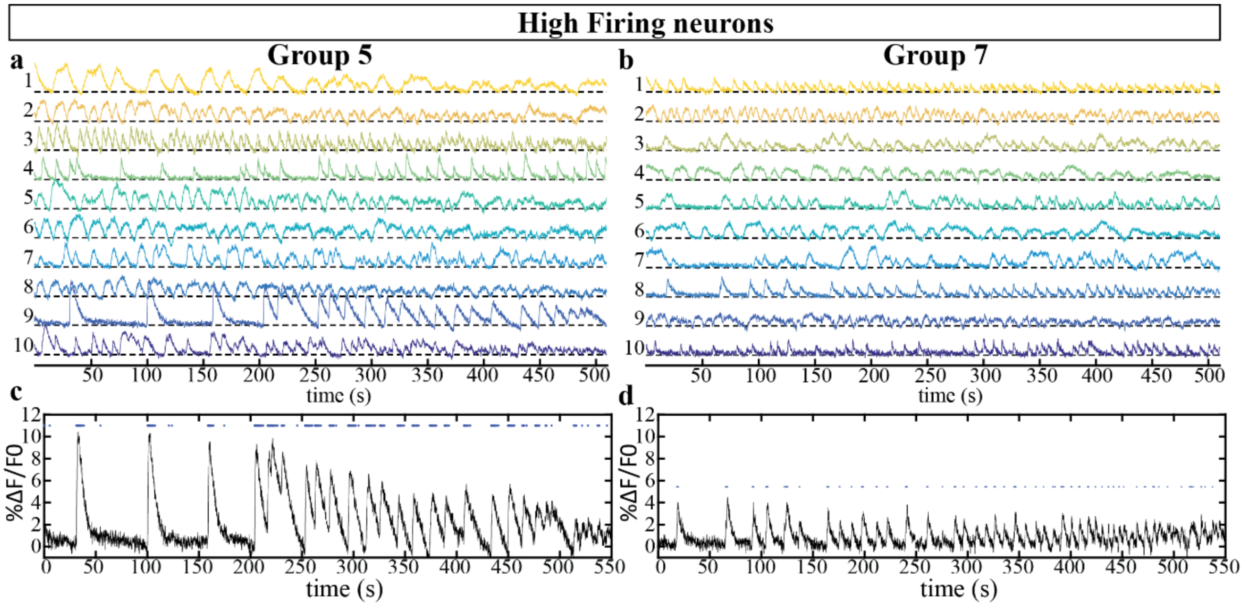

Fig. S15 Illustrative Ctr-Q33 hiPSC-derived neurons classified as ‘high firing neurons’ (HFN) at DIV 37. (a)-(b) Representative individual fluorescence traces from group G5 and G7. (c) Magnified fluorescence trace of neuron 9 from group G5. (d) Equivalent magnification of neuron 8 from group G7. Both neurons exhibit a large number and long-lasting bursts. Inferred spikes are represented with the upper blue-dots. All fluorescence traces are normalized as ΔF/ F0.

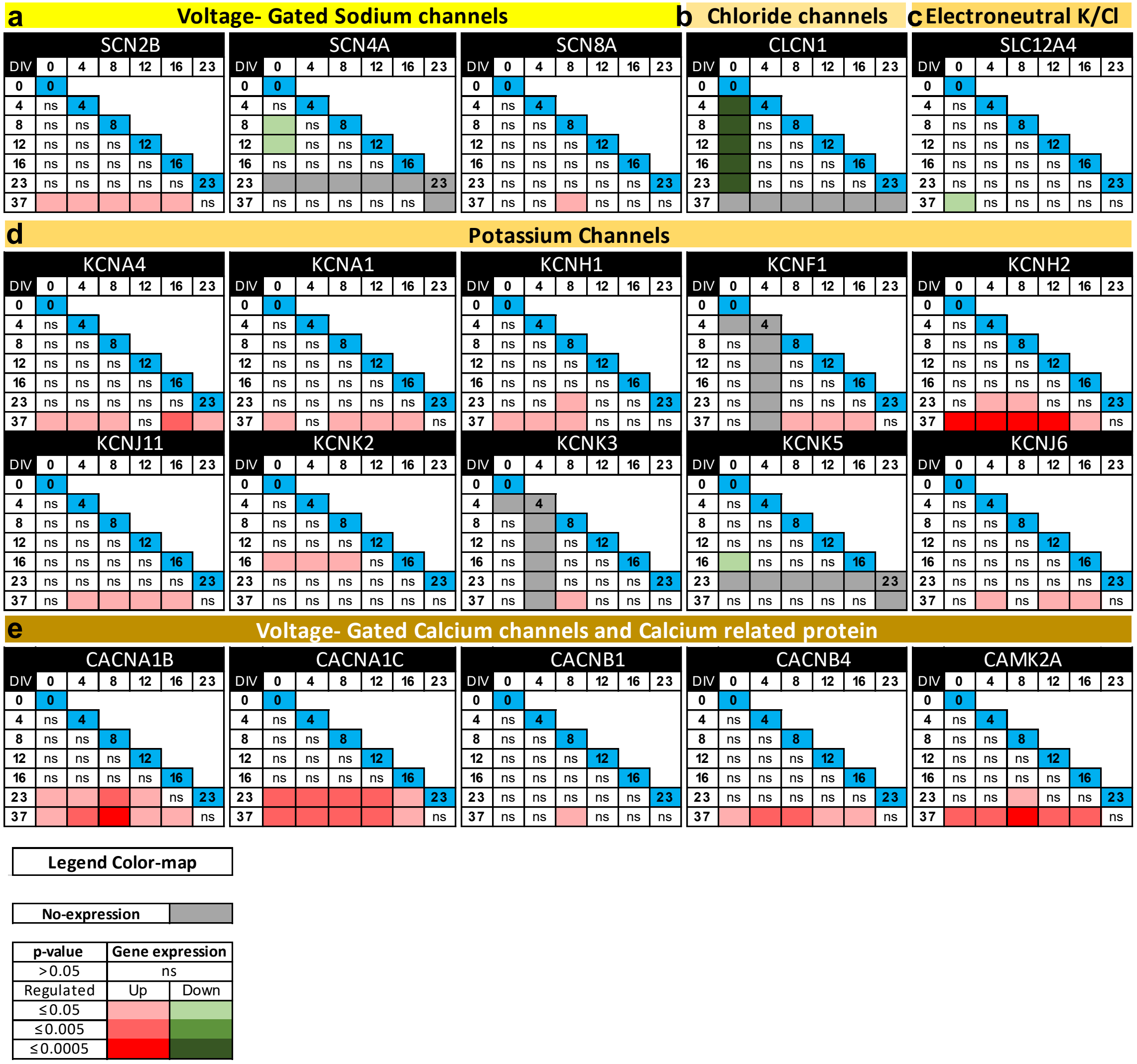

Fig. S16 Expression of Voltage-Gated ion channels by Ctr-Q33 hiPSC along the neuronal differentiation. Quantitative gene expression of (a) VGNC (b) VGCC (c) the K/Cl co-transporter, (d) VGKC and (e) VGCC by Ctr-Q33 hiPSC along the differentiation. Represented in colour-matrices, the gene expression comparison for each DIV. One-way ANOVA followed by Tukey's multiple comparison test was performed between DIV (0 to 37). Colored matrices represent all possible comparisons on the diagonal (Blue box; DIV on which the statistical test is done for all the multiple comparisons); Non-expressing genes are represented as grey boxes. Color-boxes are expressing genes with $p < 0.05$. Up-regulated and down-regulated genes are represented by red and green respectively with increasing three color-scale intensities for $P \leq 0.5$, $P \leq 0.005$ and $P \leq 0.0005$ respectively.

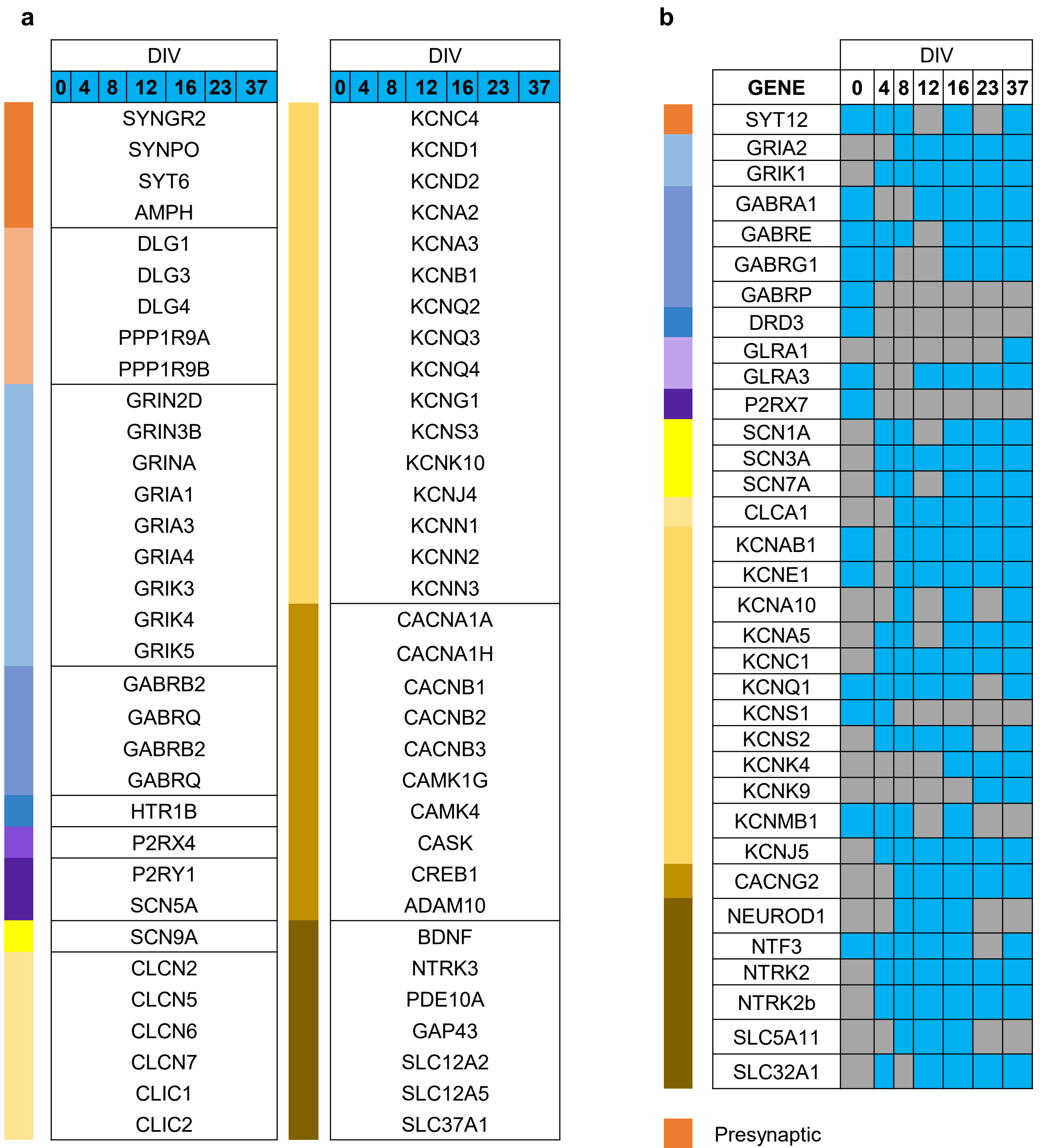

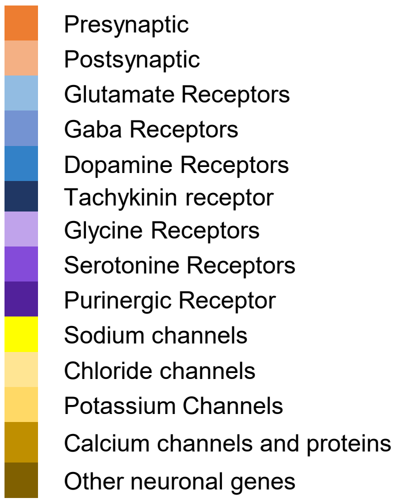

Fig. S17 List of mature genes that do no show significant changes along the neuronal differentiation of Ctr-Q33. (a) Genes that are expressed all DIVs and (b) genes that are not expressed at all DIVs. Gene are represented from DIV 0 to DIV 37 and clustered according to its cellular function as indicated in the right-bottom colour-panel. Gray boxes represents no gene-expression and blue box expression but without significance compared to the other days. One-way ANOVA followed by Turkey multiple comparison test, significance value p-value ≥ 0,05.

Protocol	hSC	Components	DIV	TUJ	MAP2	GABA	DARPP32	CTIP2
**Aubry et al. 2008**	PSC	Feeder MS5 cells (Day 0-21/23). MO-GO: BDNF, SHH, DKK-1, dbcAMP and VPA	62 days	About 30%	22,2%	36%* (7,8%+)	53%* (11,8%+)	-
**The HD iPSC Consortium (2012)**	iPSC	Feeder layer+ FGF2 (Aggregated to generate NPC). MO-GO: BDNF, rhSHH, DKK-1, dbcAMP, VPA	86 days	-	10%	-	5%* (0,5%+)	-
**Delli Carri et al 2012**	iPSC ESC	MO-GO: Dorsomorphin, Noggin, SB431542, SHHC-25II and DKK-1 (Day 0-15); B27 and BDNF (Day 15-onwards)	80 days	-	51%	78,4%* (40%+)	20%* (10,2%+)	60,3%* (30,7%+)
**Ma et al 2013**	ESC	MO: SHH and RA. Day 26-47; MO-GO-NT-O: BDNF, GDNF, IGF, AA and cAMP	47 days	43,2%	-	90,2%# (40%+)	89,7%# (38,7%+)	-
**Victor et al. 2014**	Postnatal fibroblast	miR-9/9*-124; miR-BCL11, DLX1/2, MYT1L	35 days	87%	90%	90%* (81%+)	70%* (63%+)	-
**Telezhkin et al 2015 + This work**	iPSC	Day 0-16; MO: SB, LDN, IWR1. Day 16-37; MO-GO-NT-O: PD, DAPT, CH, B27-RA, AA GABA, FK, BDNF and Ca2+	37 days	-	94,5%	29,8%* (28,1%+)	6,3%* (5,9%+)	54,8* (51,8%+)
	ESC			-	98,3%	46,6* (45,8%+)	4,2%* (4,1%+)	42,7 (41,9%+)
**Lin et al. 2015**	NPC	MO-GF: BDNF, SHH, DKK-1, Y-27632	60 days	-	63%	96%* (60,5%+)	21%* (13,2%+)	-
	iPSC-derived NPC			-	69%	965* (66,2%+)	86%* (60%+)	-
**Arber et al. 2015**	iPSC ESC	Day 0-5/9; MO: SB, LDN. Day 9-22; MO: Activine (Act). Day 25-36; MO: Act, GDNF, BDFN	43 days	-	-	-	20-50%	10-80%

Fig. S18 List of MSNs differentiation protocols from hSC. Relative to # BIII-Tubulin, to * MAP2 and to + Dapi. Abbreviations: TUJ: βIII-Tubulin, MO: morphogen, GO: Growth Factors, NT: Neurotransmitter.

a

Cell line	Methods	Time of analysis			Total
		1 month	3 months		
Ctr-Q33-GFP	Tissue processing	IHC	IHC	TEM	17
	Number of mice	6	8	3	

b

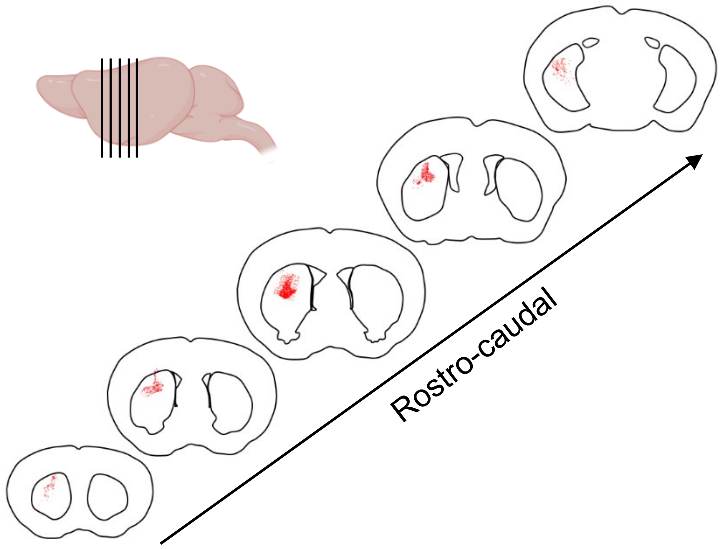

Transplanted mice (3 months)	Graft volume ($x10^6$ μm^3)
Mouse #1	87
Mouse #2	103
Mouse #3	79
Mouse #4	90
Mouse #5	81
Mouse #6	96
Mouse #7	112
Mouse #8	78
**Mean ± SEM**	91 ± 4.3

c

Cell marker	Time of analysis	Total number of cells (Mean ± SEM)
**MAP2**	1 month	3188.7 ± 153.6
**CTIP2**	1 month	3462.0 ± 158.1
	3 months	3754.7 ± 690.0
**DARPP-32**	1 month	266.1 ± 49.4
	3 months	230.5 ± 76.9
**HNA**	1 month	3948.0 ± 194.7
	3 months	4394.0 ± 705.9

Fig. S19 Assessment of graft size and neuronal fate in cell transplantation experiments. (a) Table indicating the number of transplanted mice employed for immunohistochemical (IHC) or transmission electron microscopy (TEM) analysis at 1 and 3 months PST. (b) Representative schema of rostro-caudal spreading of transplanted cells and quantification of graft volume at 3 months PST (*n*=8). (c) Quantification of the number of grafted human cells (HNA^+) expressing the neuronal marker MAP2 and the striatal neuronal markers CTIP2 and DARPP-32 at 1 and 3 months PST (*n*=6).

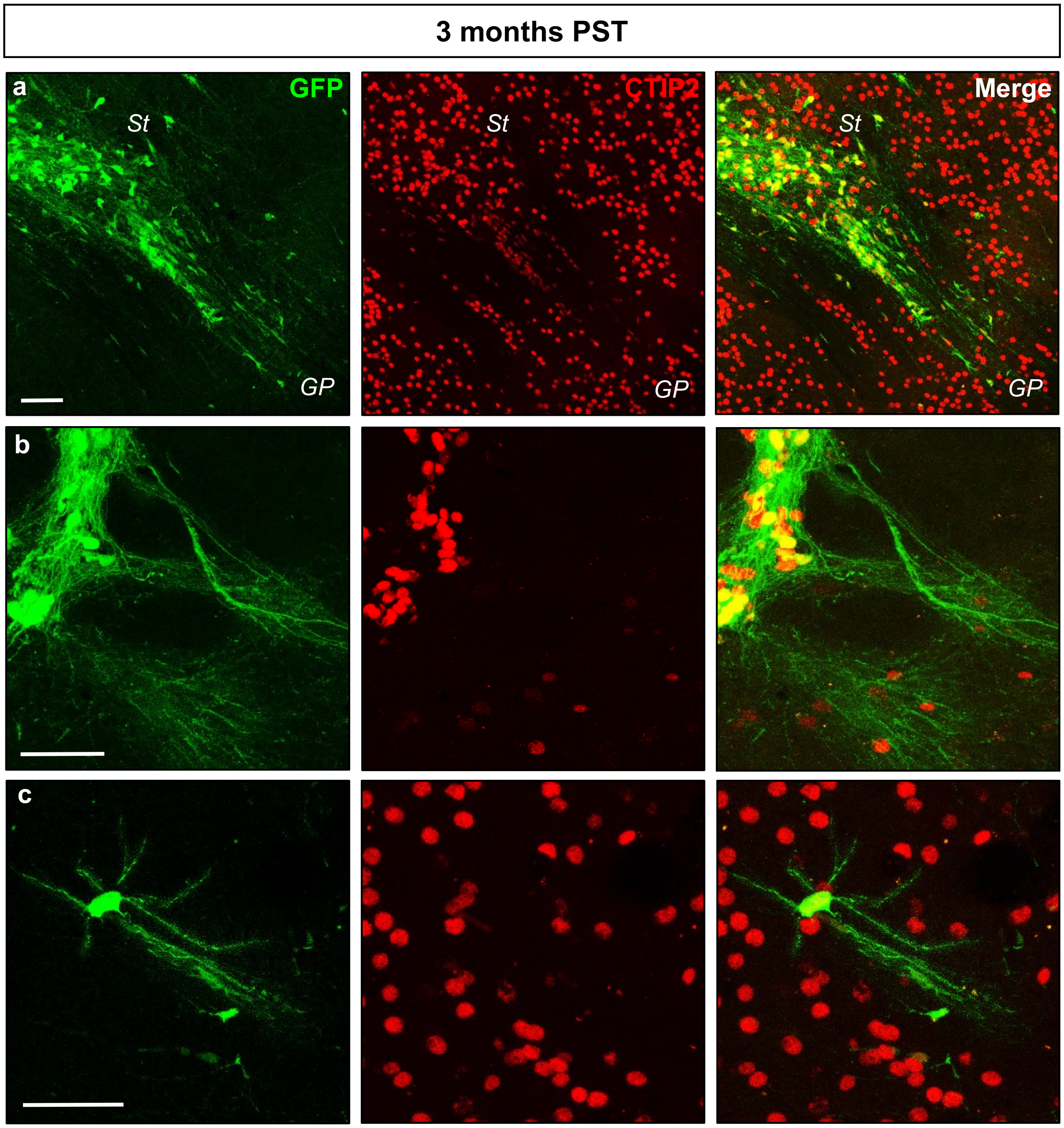

Fig. S20 (refers to Fig. 16, showing the three color channels independently). Transplanted hNPC-derived neurons project towards MSN targets. Sagittal sections of the mouse striatum showing CTIP2$^+$ human cells extending GFP$^+$ projections towards the external globus pallidus area. *St*, Striatum; *GP*, Globus pallidus. Scale bars: 50 µm
